# CD2AP promotes the progression of glioblastoma multiforme via TRIM5-mediated NF-kB signaling

**DOI:** 10.1038/s41419-024-07094-7

**Published:** 2024-10-01

**Authors:** Liang Zhang, Jiawei He, Wentao Zhao, Yuhang Zhou, Jin Li, Shaobo Li, Wenpeng Zhao, Lingliang Zhang, Ziqian Tang, Guowei Tan, Sifang Chen, Bingchang Zhang, Yun-wu Zhang, Zhanxiang Wang

**Affiliations:** 1 Department of Neurosurgery and Department of Neuroscience, the First Affiliated Hospital of Xiamen University, School of Medicine, Xiamen University, Xiamen, China; 2 Fujian Provincial Key Laboratory of Neurodegenerative Disease and Aging Research, Institute of Neuroscience, School of Medicine, Xiamen University, Xiamen, Fujian China; 3 Xiamen Neurosurgical Quality Control Center, Xiamen, China; 4 Department of Preventive Medicine, School of Public Health, Fujian Medical University, Fuzhou, Fujian Province China; 5 Department of Neurosurgery, Xiamen Humanity Hospital Fujian Medical University, Xiamen, China

**Keywords:** CNS cancer, Tumour biomarkers, Apoptosis, Cell invasion

## Abstract

CD2-associated protein (CD2AP) is a scaffolding/adaptive protein that regulates intercellular adhesion and multiple signaling pathways. Although emerging evidence suggests that CD2AP is associated with several malignant tumors, there is no study investigating the expression and biological significance of CD2AP in glioblastoma multiforme (GBM). Here by studying public datasets, we found that CD2AP expression was significantly elevated in GBM and that glioma patients with increased CD2AP expression had a worse prognosis. We also confirmed the increase of CD2AP expression in clinical GBM samples and GBM cell lines. CD2AP overexpression in GBM cells promoted their proliferation, colony formation, migration, and invasion in vitro and their tumorigenesis in vivo, and reduced cell apoptosis both at basal levels and in response to temozolomide. While CD2AP knockdown had the opposite effects. Mechanistically, we revealed that CD2AP interacted with TRIM5, an NF-κB modulator. CD2AP overexpression and knockdown increased and decreased TRIM5 levels as well as the NF-κB activity, respectively. Moreover, downregulation of TRIM5 reversed elevated NF-κB activity in GBM cells with CD2AP overexpression; and inhibition of the NF-κB activity attenuated malignant features of GBM cells with CD2AP overexpression. Our findings demonstrate that CD2AP promotes GBM progression through activating TRIM5-mediated NF-κB signaling and that downregulation of CD2AP can attenuate GBM malignancy, suggesting that CD2AP may become a biomarker and the CD2AP-TRIM5-NF-κB axis may become a therapeutic target for GBM.

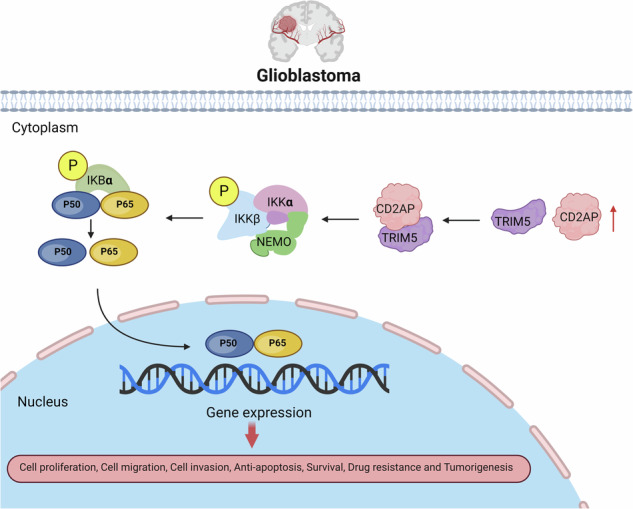

## Introduction

Glioblastoma multiforme (GBM) is the most common and aggressive malignant primary brain tumor in adults and GBM patients have a poor median survival of less than 2 years [1–5]. As GBM diffusely and strongly infiltrates the brain parenchyma, it is hard to avoid recurrence in patients under the current standard therapy consisting of a gross total tumor resection followed by radiation and treatment with the alkylating chemotherapeutic agent temozolomide [6]. Although a variety of novel GBM therapies such as immunotherapy, chimeric antigen receptor-modified T therapy, and dendritic cell vaccine are emerging, none of them have been proven effective in late-stage clinical trials [7–9]. Therefore, there is an urgent requirement for elucidating the molecular mechanisms underlying GBM progression for effective therapy development.

CD2-associated protein (CD2AP) is a scaffolding/adaptive protein and participates in multiple signaling pathways through interacting with various signaling molecules via its SH3 domains and proline-rich regions [10]. It has been reported that CD2AP is involved in dynamic actin remodeling and membrane trafficking during endocytosis and cytokinesis [11]. CD2AP is also an important adhesion-related adapter protein mediating the formation and maintenance of cell-cell contacts [12, 13]. Moreover, several studies have suggested that CD2AP is involved in certain malignant tumors [10, 14, 15]. For example, CD2AP was found to display a specific expression pattern in human urogenital organs but distinct expression patterns in several types of kidney tumors [15]. In addition, CD2AP was found to form a complex with the TKS4 protein to regulate migration and epithelial-mesenchymal transition pathways in colon cancer cells [10]. Another study showed that CD2AP expression was decreased in diffuse gastric cancer and CD2AP could inhibit gastric cancer metastasis by promoting cellular adhesion and cytoskeleton assembly [14]. Nevertheless, to our knowledge there is no study on the expression and biological significance of CD2AP in GBM to date.

NF-κB is a family of transcription factors comprising of NF-κB1/p105/p50, NF-κB2/p100/p52, RelA/p65, RelB, and c-Rel, which can form various heterodimers or homodimers and bind to consensus DNA sequences at promoter regions of responsive genes. NF-κB proteins such as NF-κB1/p105/p50 and RelA/p65 are sequestered in the cytoplasm by the IκB (inhibitor of NF-κB, such as IκBα and IκBβ) proteins in resting cells [16]. Upon stimulation, IκB kinases (IKKα, IKKβ, and NEMO) are activated and then phosphorylate inhibitory IκBs, leading to their ubiquitination and subsequent degradation [16, 17]. Thus, the transcriptionally active p50/p65 heterodimers translocate into the nucleus and regulate the expression of downstream genes. In addition to these core factors, some other proteins, such as TRIM5, also modulate the NF-κB signaling. TRIM5 was initially identified as a host antiviral protein that can block human HIV-1 infection [18, 19]. Later studies found that human TRIM5 but not its mouse homolog could promote NF-kB activity [20]. Aberrant NF-κB activation has been well-known in GBM and other malignant tumors; and activation of NF-κB can promote proliferation, survival, angiogenesis, metastasis, and invasion of tumor cells, contributing to tumor promotion and progression [21–23]. However, whether and how CD2AP regulates the NF-κB signaling, especially in GBM, remains unclear.

In this study, we found that CD2AP expression was upregulated in GBM patients and that high expression of CD2AP was associated with worse prognosis. Moreover, CD2AP overexpression promoted the malignancy of GBM cells and their tumorigenicity in the intracranial xenograft tumor model, whereas CD2AP knockdown had the opposite effects. Finally, we demonstrated that CD2AP interacted with TRIM5 to activate the NF-κB signaling for promoting GBM malignancy. Therefore, our study indicates that CD2AP may become a novel biomarker and therapeutic target for GBM treatment.

## Materials and methods

### Glioma databases and data analysis

RNA-seq expression data, clinical and molecular information of glioma patient samples were obtained from the public cancer/glioma datasets, including the Chinese Glioma Genome Atlas (CGGA, http://www.cgga.org.cn/), TCGA in UCSC Xena platform (https://xena.ucsc.edu/), Rembrandt in GEO database (https://www.ncbi.nlm.nih.gov/gds/), Gene Expression Profiling Interactive Analysis (GEPIA, http://gepia.cancer-pku.cn/), and Gravendeel in GlioVis database (http://gliovis.bioinfo.cnio.es/). Analysis of the differences in overall survival between two groups (CD2AP Low and CD2AP High) was performed using Kaplan–Meier curves and the log-rank test. The median of CD2AP expression was used as the cutoff point. Correlation was performed with Pearson analysis. Gene set enrichment analysis (GSEA) was performed using the Broad Institute GSEA version 4.1.0 software.

### Clinical samples

Glioma (WHO I-IV) biopsies from glioma patients and non-tumor brain biopsies from traumatic brain injury or epilepsy patients were collected and freshly frozen in the Department of Neurosurgery, The First Affiliated Hospital of Xiamen University. Informed consent was obtained from all patients. The study protocol was approved by the Ethics Committee of the First Affiliated Hospital of Xiamen University (XMYY-2022KY076).

### Lentivirus production

CD2AP shRNA sequences below were designed by the Thermo Scientific™ TRC Lentiviral™ shRNA Library and synthesized by TsingKe Biotech. shRNAs were inserted into the pLKO.1 vector (a kind gift from Dr. Qinxi Li).

shCD2AP#1 sense: 5′-GGAGCTGAAAGTGGGAGATAT-3′;

shCD2AP#1 antisense: 5′-ATATCTCCCACTTTCAGCTCC-3′;

shCD2AP#2 sense: 5′-GCTTCATCTCACTGCAAATAG-3′;

shCD2AP#2 antisense: 5′-CTATTTGCAGTGAGATGAAGC-3′;

Full-length human CD2AP cDNA was inserted into the lentivirus vector pBoBi (a kind gift from Dr. Qinxi Li).

For lentivirus packaging, HEK293T cells were co-transfected with constructed lentivirus vectors and packaging plasmid mixes (PSPAX and PMD2G plasmids) using the Hieff Trans Liposomal Transfection Reagent (YEA-SEN, Cat# 40802ES03). Lentiviral particles secreted into the media were harvested 48 h later.

### Cell culture and stable cell line construction

Human GBM cell lines (U87MG, U251, and SHG44), HEK293T and normal human astrocyte (HEB) cell lines were originally from the American Type Culture Collection (ATCC, USA) and maintained in our laboratory. These cells were cultured in high-glucose DMEM (Thermo Fisher) containing 10% FBS (Excell, Shanghai, China) and antibiotics (1% penicillin and streptomycin, Gibco) at 37 °C in a humidified atmosphere of 5% CO2. U87MG and U251 cells were transduced with proper amounts of lentiviruses expressing CD2AP shRNAs or CD2AP cDNA and respective controls in the presence of 10 μg/mL polybrene (YEA-SEN, Cat# 40804ES76). After 24 h of transduction, cells were selected with puromycin (Sangon Biotech, Shanghai, China, Cat# A610593-0025) to obtain stable cell lines for the following experiments.

### Quantitative real time-PCR (qRT-PCR)

Total RNA was extracted using the TRIzol reagent (YEA-SEN; Cat# 10606ES60) and cDNA was synthesized using the HiScript II Q RT SuperMix for qPCR Kit (Vazyme, Cat# R222-01), following the manufacturers’ protocols. *CD2AP* mRNA levels were measured by qRT-PCR by using 2X Universal SYBR Green Fast qPCR Mix (Abclonal, Cat# RK21203) on a StepOnePlus real-time PCR system (LightCycle). β-actin mRNA levels were utilized as an internal control. Relative mRNA expression was determined using the 2−ΔΔct method. The primer sequences are listed as the following:

CD2AP-F: 5′-GGCATGGGAATGTAGCAAGTC-3′,

CD2AP-R: 5′-CCACCAGCCTTCTTCTACCTC-3′;

CFLAR-F: 5′-TCAAGGAGCAGGGACAAGTTA-3′,

CFLAR-R: 5′-GACAATGGGCATAGGGTGTTATC-3′;

TRAF2-F: 5′-TCCCTGGAGTTGCTACAGC-3′,

TRAF2-R: 5′-AGGCGGAGCACAGGTACTT-3′;

IEX-1L-F: 5′-CAGCCGCAGGGTTCTCTAC-3′,

IEX-1L-R: 5′-GATCTGGCAGAAGACGATGGT-3′;

MNSOD-F:5′-GCTCCGGTTTTGGGGTATCTG-3′,

MNSOD-R: 5′-GCGTTGATGTGAGGTTCCAG -3′;

β-actin-F: 5′-ATCAAGATCATTGCTCCTCCTGAG-3′,

β-actin-R: 5′-CTGCTTGCTGATCCACATCTG-3′.

### Cell proliferation assay

In total, 1500 cells/well were seeded in 96-well plates. Cell proliferation, represented by the OD value measured using a microplate reader at 450 nm, was studied every 24 h using a CCK8 Kit (US EVERBRIGHT, China), following the manufacturer’s protocol.

### Colony formation assay

In total, 1000 cells/well were seeded in 6-well plates. After 14 d of cultivation, cells were fixed with 4% paraformaldehyde and stained with 0.1% crystal violet. Clones were observed under a light microscope and those with a size of 2 mm or greater were counted.

### Cell migration assay

Cells (2 × 10^4^) were seeded in the upper Transwell chamber (polycarbonate filter with 8-mm pores, Corning) containing 200 μL serum-free medium. The bottom chamber was supplemented with 600 μL complete medium as a chemo-attractant. After 48 h incubation, non-migrated cells on the upper surface of the filters were removed by a cotton swab. Migrated cells on the lower surface of the filters were fixed with 4% formaldehyde for 30 min and then stained with 0.1% crystal violet at room temperature for another 30 min. After being washed with 1× PBS, the cells were photographed under inverted microscope.

### Cell invasion assay

Cells (5 × 10^4^) were seeded in the upper Transwell chamber (polycarbonate filter with 8-mm pores, coated with 200 mg/mL Matrigel [Corning, Cat# 356234]) containing 200 μL serum-free medium. The bottom chamber was supplemented with 600 μL complete medium as a chemo-attractant. After 24 h incubation, noninvasive cells on the upper surface of the filters were removed by a cotton swab. Invasive cells on the lower surface of the filters were fixed with 4% formaldehyde for 30 min and then stained with 0.1% crystal violet at room temperature for another 30 min. After being washed with 1× PBS 3 times, the cells were photographed under inverted microscope.

### Cell apoptosis analysis by flow cytometry

Treated cells were harvested and incubated with an Annexin V-fluorescein isothiocyanate (FITC)/propidium iodide (PI) apoptosis detection kit (BD Biosciences, Franklin Lakes, NJ, USA, Cat# 556547), following the manufacturer’s protocol. Stained cells were analyzed with a flow cytometer.

### Western blot and co-immunoprecipitation (co-IP)

Brain tissue samples or cells were lysed with 1% TNEN buffer, and centrifuged at 4 °C, 12,000 × *g* for 15 min. Supernatants were collected and the protein concentration was quantified using the bicinchoninic acid assay method. Equal amounts of proteins (20 μg) were separated by SDS-PAGE and transferred to a PVDF membrane. After blocking with 5% skim milk at room temperature for 1.5 h, the membranes were incubated with primary antibodies at 4 °C overnight. After washing with Tris-buffered saline/1% Tween-20 (TBS-T) for 0.5 h, the membranes were incubated with appropriate HRP-conjugated secondary antibody for 1.5 h. After washing with TBS-T buffer again, the membranes were scanned with the Azure C300 Imaging System (USA).

For Co-IP assay, cell lysates were incubated with primary antibodies and protein A/G MagBeads at 4 °C overnight, Immunoprecipitated proteins were then subjected to western blot analysis.

Antibodies used include: rabbit anti-CD2AP (Cat# 51046-1-AP, 1:2000), rabbit anti-HA (Cat# 51064-2-AP, 1:2000), and rabbit anti-GFP (Cat# 16825-1-AP, 1:2000) from Proteintech; rabbit anti-NF-κB p65 (Cat# AF5243, 1:1000), rabbit anti-p-NF-κB p65 (site 536, Cat# AF5881, 1:1000), and rabbit anti-p-IκBα (Cat# AF1870, 1:1000) from Beyotime; rabbit anti-IKKβ (Cat# 8943S, 1:1000), rabbit anti-IκBα (Cat# 4812S, 1:1000), rabbit anti-caspase-3 (Cat# 9662S, 1:1000), rabbit anti-cleaved caspase-3 (Cat# 9664S, 1:1000), rabbit anti-PARP (Cat# 9532S, 1:1000), rabbit anti-cleaved PARP (Cat# 5625S, 1:1000), rabbit anti-caspase-8 (Cat# 9746S, 1:1000), and rabbit anti-cleaved caspase-8 (Cat# 9496S, 1:1000) from Cell Signaling Technology; mouse anti-TRIM5 (Cat# SC-373864, 1:100) from Santa Cruz Biotechnology; and rabbit anti-GAPDH (Cat# AB0036, 1:10000) from Abways.

### In vivo intracranial xenograft tumor model

6-week-old female BALB/c nude mice were used for tumor cell xenograft as previously reported [24–27]. Mice were randomly separated into indicated groups (*n* = 8 per group for CD2AP overexpression study and *n* = 9 per group for CD2AP knockdown study). After anesthesia, 1 × 10^5^ cells were transplanted into the right frontal lobe of each mouse by a microinjector. Mouse body weight and living status were recorded. 4 weeks after implantation, mice were anaesthetized and then subjected to cerebral magnetic resonance imaging (MRI). T1-weighted serial coronal images of brain were acquired at 1 mm intervals with a 25 × 25 mm field and a 256 × 256 pixel resolution. Tumor volumes were calculated as (length × width^2^)/2 using RadiAnt DICOM Viewer software (Version 2020.2.3). At the end of the experiment, survival mice were sacrificed and brains were dissected for histopathologic staining. All animal experiments were performed in a non-blinded manner. Animal procedures were in accordance with the guidelines of the National Institutional Animal Health Guide for the Care and Used of Laboratory Animals and approved by the Animal Ethics Committee of Xiamen University (XMULAC20230235).

### H&E staining

Tumor tissues were fixed with 4% paraformaldehyde, embedded in paraffin and sectioned (4 μm). Brain slices were stained with hematoxylin for 5 min and 1% hydrochloric alcohol for 30 s. After washing with distilled water, brain slices were stained by eosin for 3 min. Finally, the slices were sealed with neutral resin and photographed under a light microscope (Olympus, Tokyo, Japan).

### Bimolecular fluorescence complementation (BiFC) assays

HEK 293 T cells were transfected with plasmids expressing TRIM5 fused with the N-terminal fragment of Venus and/or CD2AP fused with the C-terminal fragment of Venus. Fluorescence was observed under a Revolve Microscope (Zeiss).

### Transmission electron microscope

Treated cells were harvested, fixed in 2.5% glutaraldehyde for 3 h at 4 °C, and postfixed in 1% osmium tetroxide for 1 h at 4 °C. The cell masses were further dehydrated with graded alcohol, embedded in resin, and cut using an ultramicrotome (Leica, German). The ultrathin sections (60–80 nm) were mounted on copper grids, stained with uranyl acetate and lead citrate, and then observed under a transmission electron microscope (HT-7800, Hitachi, Japan).

### Cytoplasmic and nuclear protein extraction

Cytoplasmic and nuclear protein extraction was conducted with a commercial kit, (Beyotime, Cat# P0028), following the manufacturer’s instructions. Briefly, cells were harvested and suspended with appropriate volume of solution A containing 1% PMSF and placed on ice for 15 min. After mixing with 10 μL solution B, samples were vortexed fiercely and then placed on ice for another 1 min. After 12,000 × *g* centrifugation for 5 min, the supernatant containing cytoplasmic proteins was transferred to another tube for future use. The sediment was resuspended with 50 μL solution C and vortexed fiercely for 30 s every 2 min for 15 times, and nuclear proteins in the supernatant were obtained after centrifugation.

### Protein mass spectrometry

To identify CD2AP-interacting proteins, we used an CD2AP antibody and IgG to immunoprecipitate proteins in U87MG cells. Pulled down proteins were first subjected to SDS–PAGE. After staining with Coomassie Brilliant Blue R-250 dye, gels were decoloured and the excised gel segments were subjected to in-gel trypsin digestion and dried. Samples were then analyzed on a nanoElute (Bruker) coupled to a timsTOF Pro (Bruker) equipped with a CaptiveSpray source. Peptides were dissolved in 10 μL 0.1% formic acid (v/v) and loaded onto a homemade C18 column (35 cm × 75 μm, ID of 1.9 μm, 100 Å). Samples were then eluted for 60 min with linear gradients of 3–35% acetonitrile (v/v, in 0.1% formic acid) at a flow rate of 0.3 μl min^–1^. MS data were acquired with a timsTOF Pro mass spectrometer (Bruker) operated in PASEF mode, and analyzed using Peaks Studio software (X+, Bioinformatics Solutions). The human UniProt Reference Proteome database was used during data analysis.

### Graphical abstract creation

The graphical abstract is created in BioRender. Zhang, L. (2024) BioRender.com/d30h566.

### Statistical analyses

Statistical analyses were performed using Graphpad Prism 8.0 software. No samples were excluded from analysis. All quantitative data were presented as the mean ± SEM. Variances were similar between groups for comparisons. Two-tailed unpaired *t*-test was used to compare the differences between two groups. One-way ANOVA and two-way ANOVA with post hoc tests were used for comparisons between multiple groups. Kaplan–Meier survival analysis was performed using log-rank analysis. *P* < 0.05 was considered as statistically significant.

## Results

### The expression of CD2AP is significantly upregulated in GBM patients

**Fig. 1:**
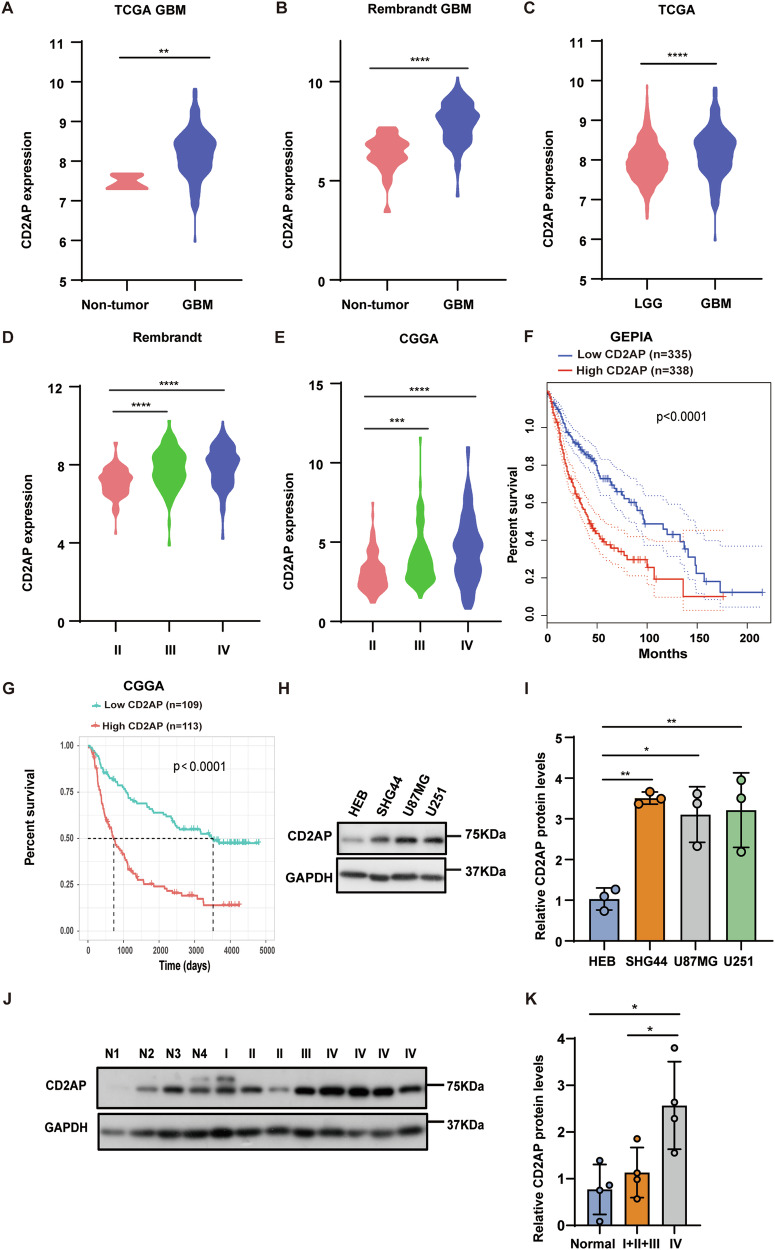
CD2AP expression is significantly upregulated in GBM patients. **A** CD2AP expression comparison between GBM and non-tumor samples using the TCGA database. Unpaired *t-*test, *n* = 5 for non-tumor and *n* = 167 for GBM. **B** CD2AP expression comparison between GBM and non-tumor samples using the Rembrandt database. Unpaired *t-*test, *n* = 28 for non-tumor and *n* = 221 for GBM. **C** CD2AP expression comparison between GBM and low-grade glioma (LGG) using the TCGA database. Unpaired t-test, *n* = 529 for LGG and *n* = 167 for GBM. **D** CD2AP expression comparison between different WHO grades (II, III, and IV) of glioma using the Rembrandt database. One-way ANOVA with Tukey’s post hoc test, *n* = 99 for grade II, *n* = 85 for grade III, and *n* = 221 for grade IV. **E** CD2AP expression comparison between different WHO grades of glioma using the CGGA database. One-way ANOVA with Tukey’s post hoc test, *n* = 103 for grade II, *n* = 79 for grade III, and *n* = 139 for grade IV. **F** Kaplan–Meier analysis of progression-free survival using data from the GEPIA database. Log-rank test. **G** Kaplan–Meier analysis of progression-free survival using data from the CGGA database. Log-rank test. Equal amounts of cell lysates of HEB and various GBM cell lines were subjected to western blotting (**H**) and quantitative comparison (**I**) for CD2AP. GAPDH was used as an internal loading control. One-way ANOVA with Tukey’s post hoc test. *n* = 3 per group. Equal amounts of protein lysates of 4 non-tumor brain biopsies from traumatic brain injury or epilepsy patients (N1-N4), 4 glioma samples (grades I, II, and III), and 4 GBM samples (grades IV) were subjected to western blotting (**J**) and quantitative comparison (**K**) for CD2AP. One-way ANOVA with Tukey’s post hoc test. Data represent mean ± SEM, *P < 0.05, **P < 0.01, ***P < 0.001, ****P < 0.0001.

To study whether CD2AP is involved in the malignant progression of GBM, we first analyzed datasets from the TCGA, Rembrandt, and CGGA databases. We found that CD2AP expression was significantly elevated in GBM samples compared to non-tumor tissues (Fig. 1A, B) and low-grade glioma samples (Fig. 1C). In addition, CD2AP expression was increased considerably along with the deteriorated stages of GBM (the WHO grades defined by histopathologic criteria, Fig. 1D, E). Moreover, CD2AP expression was negatively correlated with the overall survival of glioma patients in both the GEPIA (Fig. 1F) and the CGGA datasets (Fig. 1G). Isocitrate dehydrogenase (IDH) mutations and chromosomal arms 1p and 19q (1p/19q) codeletions favor longer survival of GBM patients and are important molecular biomarkers for GBM diagnosis and treatment [28, 29]. We also noticed that CD2AP expression was lower in GBM samples with IDH mutations and 1p/19q codeletions than in respective controls (Supplementary Fig. S1A, B). To further verify the involvement of CD2AP in GBM, we compared CD2AP levels between HEB cells and various glioma cell lines (SHG44, U87MG, and U251) and found that CD2AP levels were higher in glioma cells than in HEB cells (Fig. 1H, I). Finally, we acquired GBM tissues and brain tissues from non-tumor patients and confirmed that CD2AP levels were significantly increased in GBM (grades IV) tissues than in non-tumor tissues and low-grade tumor tissues (I, II and III) (Fig. 1J, K). Together, these results indicate that CD2AP expression is increased in GBM and that high CD2AP expression is associated with poor survival of GBM patients.

### CD2AP overexpression accelerates GBM progression both in vitro and in vivo

**Fig. 2:**
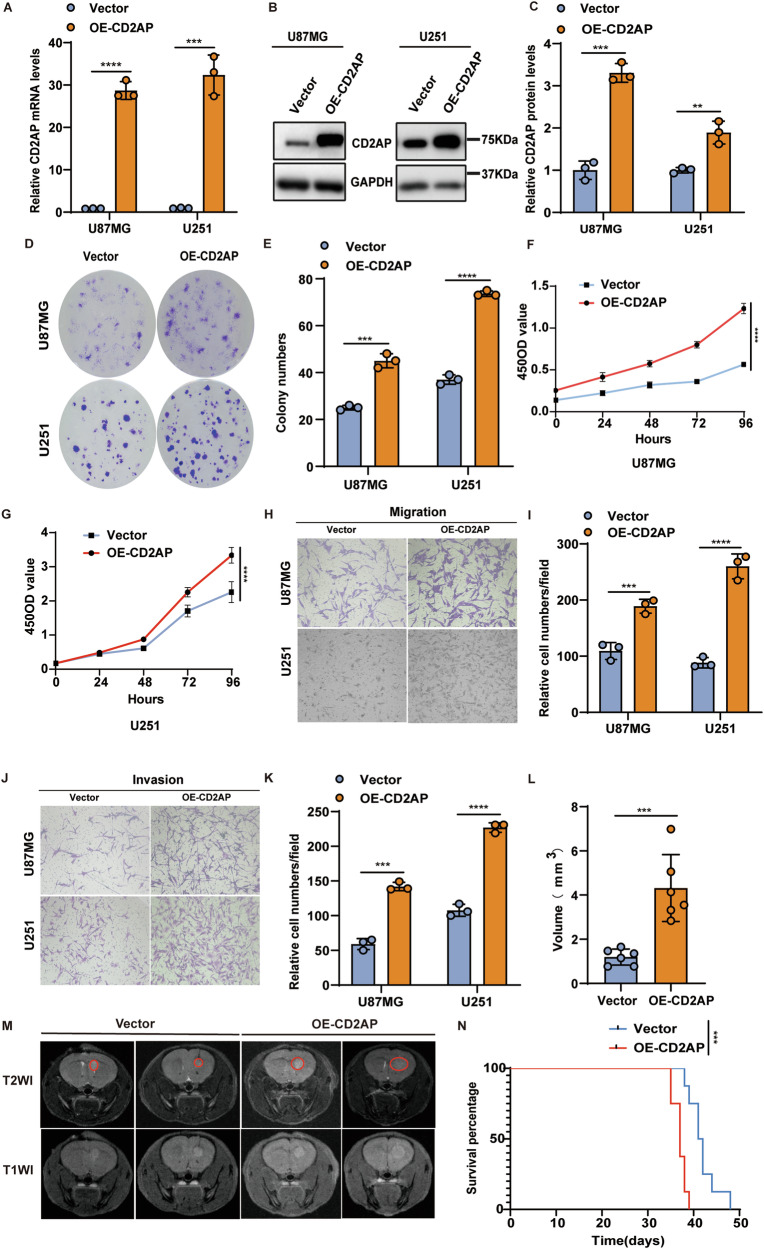
CD2AP overexpression accelerates GBM progression both in vitro and in vivo. (**A**) U87MG and U251 cells were stably transduced with lentiviruses expressing CD2AP (OE-CD2AP) or vector control and the mRNA levels of CD2AP were compared. Unpaired *t*-test, *n* = 3 per group. CD2AP protein in U87MG and U251 cells stably overexpressing CD2AP or vector control were analyzed by western blotting (**B**) and densitometry quantification for comparison (**C**). Unpaired *t*-test, *n* = 3 per group. U87MG and U251 cells with stable CD2AP expression or vector control were assayed for colony formation (**D**) and the colony numbers were compared (**E**). Unpaired *t-*test, *n* = 3 per group. U87MG (**F**) and U251 (**G**) cells with stable CD2AP expression or vector control were assayed for their proliferation. Two-way ANOVA with Sidak’s post hoc test, *n* = 6 per group for U87MG and *n* = 10 per group for U251. Cells with stable CD2AP overexpression or vector control were studied for their migration ability (**H**, **I**) and invasion ability (**J**, **K**). Unpaired *t-*test, *n* = 3 per group. U87MG cells with stable CD2AP expression or vector control were xenografted into the mouse brain. Tumor formation was observed by MRI brain images (**M**) and tumor volumes were compared (**L**). Red cycles indicate tumor areas. Unpaired *t-*test, *n* = 6 per group. **N** Kaplan–Meier survival curves of xenografted mice. Log-rank test, *n* = 8 per group. Data represent mean ± SEM, **P < 0.01, ***P <0.001, ****P < 0.0001.

To study the functional role of CD2AP in GBM, we transduced U87MG and U251 cells with lentiviruses expressing CD2AP or control and selected cells stably overexpressing CD2AP with puromycin. Both qRT-PCR and western blot analysis confirmed elevated CD2AP expression in these cells (Fig. 2A–C). We found that CD2AP overexpression markedly promoted colony formation (Fig. 2D, E) and cell proliferation (Fig. 2F, G). Moreover, we performed migration and matrigel invasion assays and found that CD2AP overexpression resulted in increased cell migration (Fig. 2H, I) and cell invasion abilities (Fig. 2J, K). We further noticed that CD2AP overexpression reduced cell apoptosis (Supplementary Fig. S2A, B).

To evaluate the role of CD2AP overexpression in tumorigenesis in vivo, we xenografted U87MG cells with CD2AP overexpression and control cells into the brain of nude mice. About one month later, all xenografted mice developed brain tumors that were clearly detected by MRI (Fig. 2M) but cells with CD2AP overexpression formed bigger tumors than control cells (Fig. 2L, M, and Supplementary Fig. S2C). The average body weight between mice xenografted with CD2AP-overexpressing cells and with control cells were not significantly different (Supplementary Fig. S2D) and both mice gained some weight at the beginning (Supplementary Fig. S2E). But at late stages after xenografting, the average body weight of mice xenografted with CD2AP-overexpressing cells dropped and were significantly lighter than that with control cells (Supplementary Fig. S2E). Moreover, mice xenografted with CD2AP-overexpressing cells (median survival time: 37 days) had reduced survival time compared to control mice (median survival time: 41.5 days) (Fig. 2N). These results suggest that elevated CD2AP levels promotes malignancy of GBM cells.

### CD2AP knockdown reduces the malignancy of GBM cells both in vitro and in vivo

**Fig. 3:**
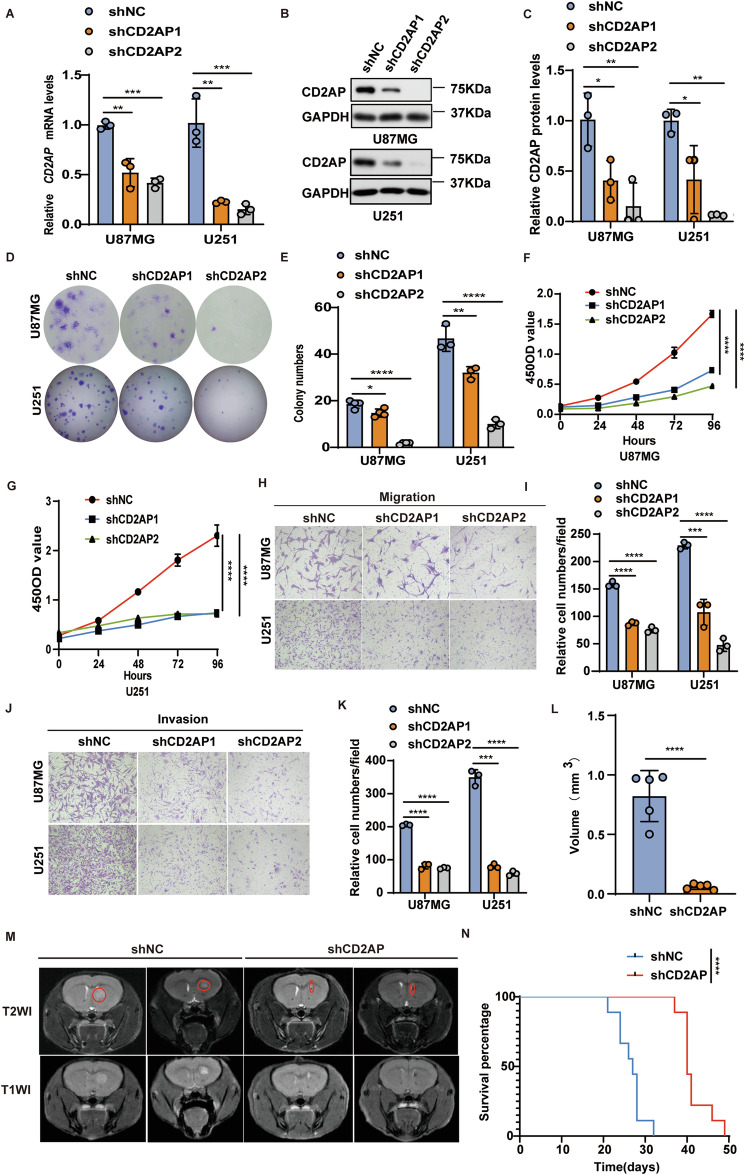
CD2AP knockdown reduces the malignancy of GBM cells both in vitro and in vivo. **A** U87MG and U251 cells were stably transduced with two CD2AP shRNA lentiviruses (shCD2AP1 and shCD2AP2) or control shRNA lentiviruses (shNC); and the mRNA levels of CD2AP were compared. One-way ANOVA with Tukey’s post hoc test, *n* = 3 per group. CD2AP protein in U87MG and U251 cells stably transduced with CD2AP shRNA or control shRNA lentiviruses were analyzed by western blotting (**B**) and densitometry quantification for comparison (**C**). One-way ANOVA with Tukey’s post hoc test, *n* = 3 per group. U87MG and U251 cells with CD2AP knockdown were assayed for colony formation (**D**) and the colony numbers were compared (**E**). One-way ANOVA with Tukey’s post hoc test, *n* = 4 per group. U87MG (**F**) and U251 (**G**) cells with CD2AP knockdown were assayed for their proliferation. Two-way ANOVA with Sidak’s post hoc test, *n* = 8 per group for U87MG and *n* = 10 per group for U251. U87MG and U251 cells with CD2AP knockdown were studied for their migration ability (**H**, **I**) and invasion ability (**J**, **K**). One-way ANOVA with Tukey’s post hoc test, *n* = 3 per group. U87MG cells with CD2AP knockdown (shCD2AP) and controls (shNC) were xenografted into the mouse brain. Tumor formation was observed by MRI brain images (**M**) and tumor volumes were compared (**L**). Red cycles indicate tumor areas. Unpaired *t-*test, *n* = 5 per group. **N** Kaplan–Meier survival curves of xenografted mice. Log-rank test, *n* = 9 per group. Data represent mean ± SEM, *P < 0.05, **P < 0.01, ***P < 0.001, ****P < 0.0001.

Next, we transduced U87MG and U251 cell lines with lentiviruses expressing two different CD2AP shRNAs (shCD2AP1 and shCD2AP2) or a scrambled control shRNA (shNC). After puromycin selection, cells were studied for CD2AP mRNA and protein levels to confirm the knockdown of CD2AP (Fig. 3A–C). CD2AP knockdown markedly reduced colony formation (Fig. 3D, E), cell proliferation (Fig. 3F, G), cell migration (Fig. 3H, I), and cell invasion (Fig. 3J, K). CD2AP knockdown also promoted cell apoptosis (Supplementary Fig. S3A, B); and cells with CD2AP knockdown exhibited elevated cleavages of PARP, caspase-8, and caspase-3 that indicates apoptosis (Supplementary Fig. S3C–E) and apoptotic morphologies (Supplementary Fig. S3F).

When cells were xenografted into the brain of nude mice, we found that U87MG cells with CD2AP knockdown formed smaller tumors than control cells (Fig. 3L, M and Supplementary Fig. S3G). Although the average body weight between mice xenografted with CD2AP-knockdown cells and with control cells were not different at the beginning (Supplementary Fig. S3H), the average body weight of mice xenografted with control cells became less than that of mice xenografted with CD2AP-knockdown cells at later stages (Supplementary Fig. S3I). Moreover, Kaplan–Meier survival curve analysis showed that mice xenografted with CD2AP-knockdown cells had much longer survival time (median survival time: 40 days) than control mice (median survival time: 27 days) (Fig. 3N). Together, these results suggest that CD2AP knockdown reduces GBM cell malignancy. We also noticed that the median survival time of control mice used for CD2AP knockdown experiments was shorter than that of control mice used for CD2AP overexpression experiments. One possible explanation for this is a batch difference between mice used for the two experiments, as the average weight of the formal is about 22 g (Supplementary Fig. S3H) and that of the latter is about 17 g (Supplementary Fig. S2D). Nevertheless, since we used a same batch of animals for each experiment and there were no weight differences between control and experimental animals, the data are comparable within each experiment.

### CD2AP regulates the NF-κB signaling in GBM

**Fig. 4:**
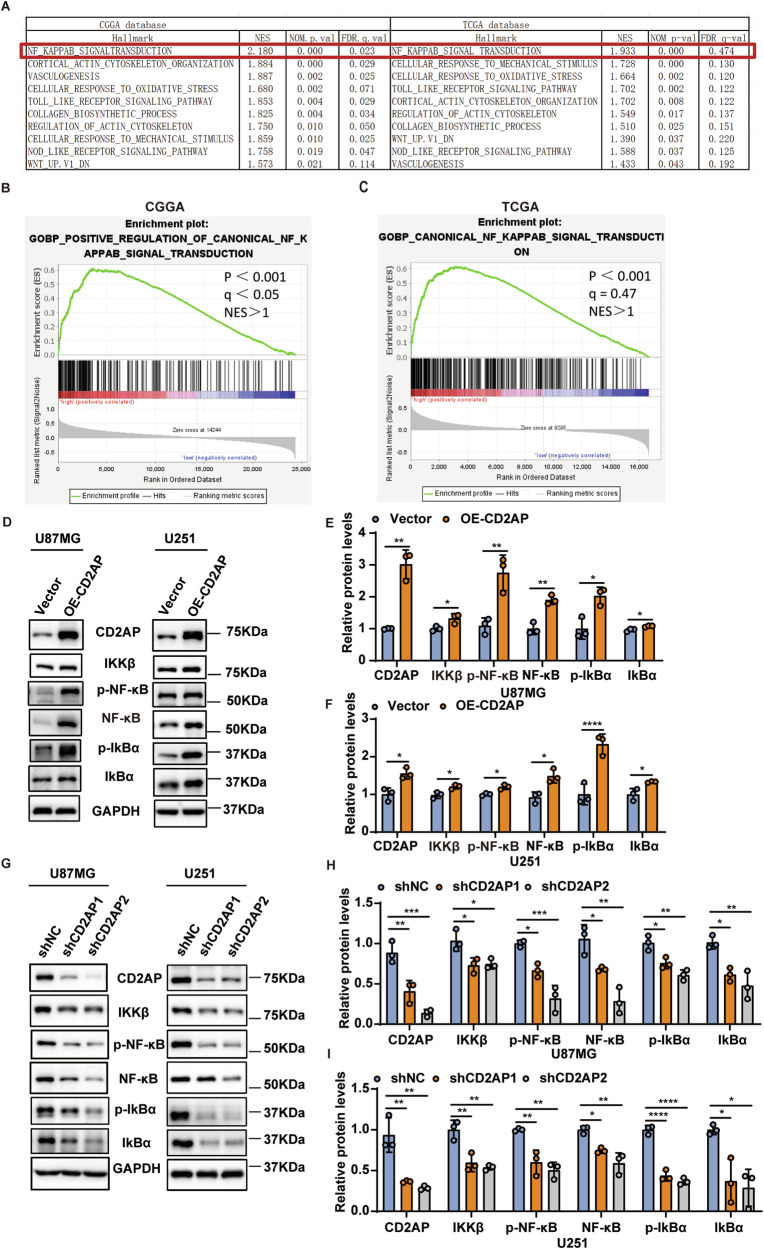
CD2AP activates the NF-κB signaling in GBM. (**A**) GSEA analysis of data from the CGGA and TCGA databases. GSEA analysis of the correlation between CD2AP expression and NF-κB signaling pathway based on CGGA (**B**) and TCGA (**C**) datasets. Levels of NF-κB signaling-related proteins in U87MG and U251 cells with stable CD2AP expression (OE-CD2AP) or vector control were analyzed by western blotting (**D**) and densitometry quantification comparison (**E**, **F**). Unpaired *t*-test, *n* = 3 per group. Levels of NF-κB signaling-related proteins in U87MG and U251 cells with stable CD2AP knockdown were analyzed by western blotting (**G**) and densitometry quantification comparison (**H**, **I**). One-way ANOVA with Tukey’s post hoc test, *n* = 3 per group. Data represent mean ± SEM, *P < 0.05, **P < 0.01, ***P <0.001, ****P < 0.0001.

To explore the underlying mechanism by which CD2AP regulates GBM cell malignancy, we performed GSEA analysis using the CGGA and TCGA data and found that CD2AP was positively correlated with multiple molecular pathways of malignant tumors (Fig. 4A), including the NF-κB signaling (Fig. 4B, C).

Because the NF-κB signaling has been reported to play a pivotal role in promoting GBM progression [30–32], we further analyzed how CD2AP affects the NF-κB signaling. Overexpression of CD2AP markedly increased protein levels of NF-κB/p65, phosphorylated (p-)NF-κB/p65, IKKβ, IκBα, and p-IκBα (Fig. 4D–F), whereas knockdown of CD2AP reduced levels of these proteins (Fig. 4G–I). Because p-NF-κB/p65 at S536 site is important for its nuclear translocation and transcription activity [33], we studied the cytoplasmic and nuclear distribution of p-NF-κB (S536) and found that it predominantly localized in the nuclear fraction, and CD2AP overexpression markedly increased its levels in the nuclear fraction (Supplementary Fig. 4A, B). On the other hand, total NF-κB was found mainly localized in the cytoplasmic fraction and CD2AP overexpression increased its levels in the cytosol (Supplementary Fig. 4A, C). Moreover, we found that the expressions of several known NF-κB target genes conferring antiapoptotic properties, such as CFLAR, TRAF2, IEX-1L, and MNSOD [21] were elevated in GBM cells with CD2AP overexpression (Supplementary Fig. S4D, E). Together, these findings show that CD2AP regulates the NF-κB signaling in GBM.

### Inhibition of NF-kB activity constrains the malignancy of GBM cells

**Fig. 5:**
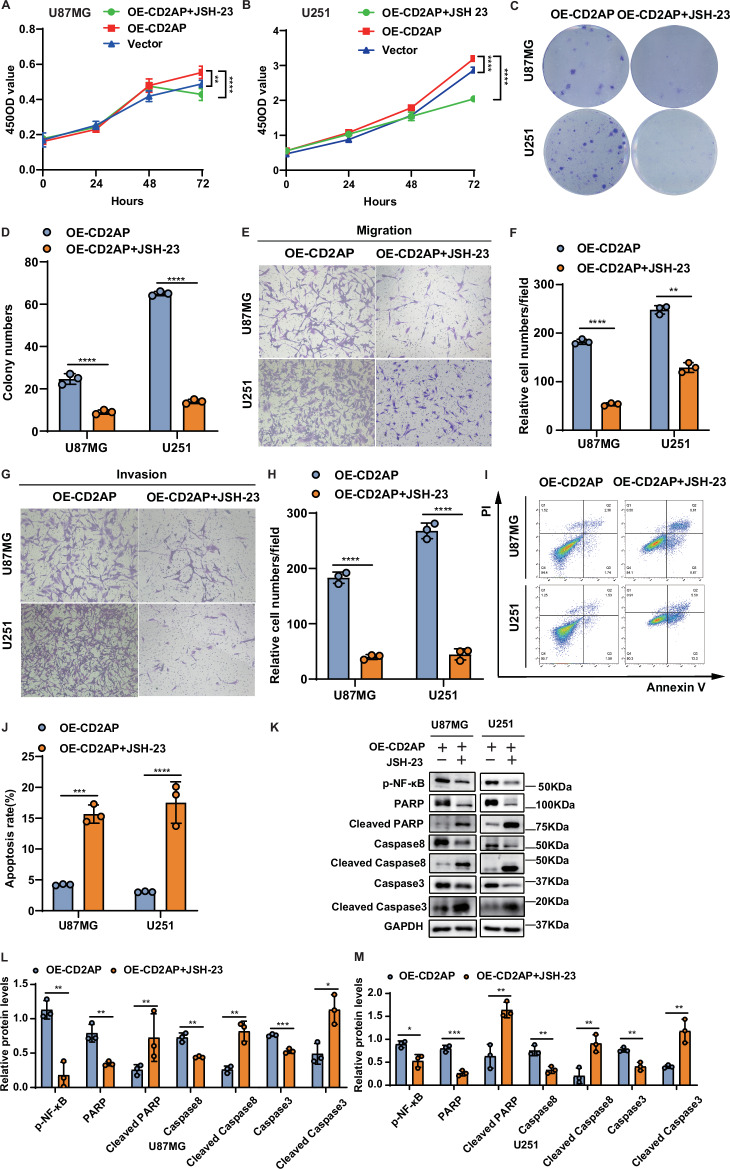
Inhibition of the NF-kB activity reduces the malignancy of GBM cells with CD2AP overexpression. U87MG (**A**) and U251 (**B**) cells with stable CD2AP expression (OE-CD2AP) were treated with or without the NF-kB inhibitor JSH-23 (80 μM) for indicated time periods and cell proliferation was analyzed. Cells transfected with control vector were used as a negative control. Two-way ANOVA with Sidak’s post hoc test, *n* = 4 per group for U87MG and *n* = 8 per group for U251. U87MG and U251 cells with stable CD2AP overexpression were treated with 80 μM JSH-23 and analyzed for their colony formation ability (**C**) and colony numbers (**D**). Unpaired *t-*test, *n* = 3 per group. U87MG and U251 cells with stable CD2AP overexpression were treated with 80 μM JSH-23 and studied for their migration (**E**, **F**) and invasion (**G**, **H**) abilities. Unpaired *t*-test, *n* = 3 per group. U87MG and U251 cells with stable CD2AP overexpression were treated with 80 μM JSH-23 for 48 h and cell apoptosis was measured by flow cytometry (**I**) for apoptosis ratio comparison (**J**). Unpaired *t*-test, *n* = 3 per group. U87MG and U251 cells with stable CD2AP overexpression were treated with 80 μM JSH-23 for 72 h and equal amounts of cell lysates were subjected to western blotting (**K**) and quantification analysis (**L**, **M**) for apoptosis-related proteins. Unpaired *t*-test, *n* = 3 per group. Data represent mean ± SEM, *P < 0.05, **P < 0.01, ***P < 0.001, ****P < 0.0001.

Since CD2AP overexpression promotes NF-κB activity, we treated GBM cells stably overexpressing CD2AP with JSH-23, an NF-κB inhibitor. As expected, JSH-23 treatment reduced p-NF-κB/p65 levels (Fig. 5K), suggesting the inhibition of NF-κB activity. Importantly, JSH-23 treatment dramatically reduced proliferation (Fig. 5A, B), colony formation (Fig. 5C, D), migration (Fig. 5E, F), and invasion (Fig. 5G, H) of these cells, and increased their apoptosis (Fig. 5I–M). Together, these results suggest a critical role of CD2AP in facilitating GBM malignancy via regulating the NF-κB activity.

### CD2AP-mediated NF-kB activation is dependent on TRIM5

**Fig. 6:**
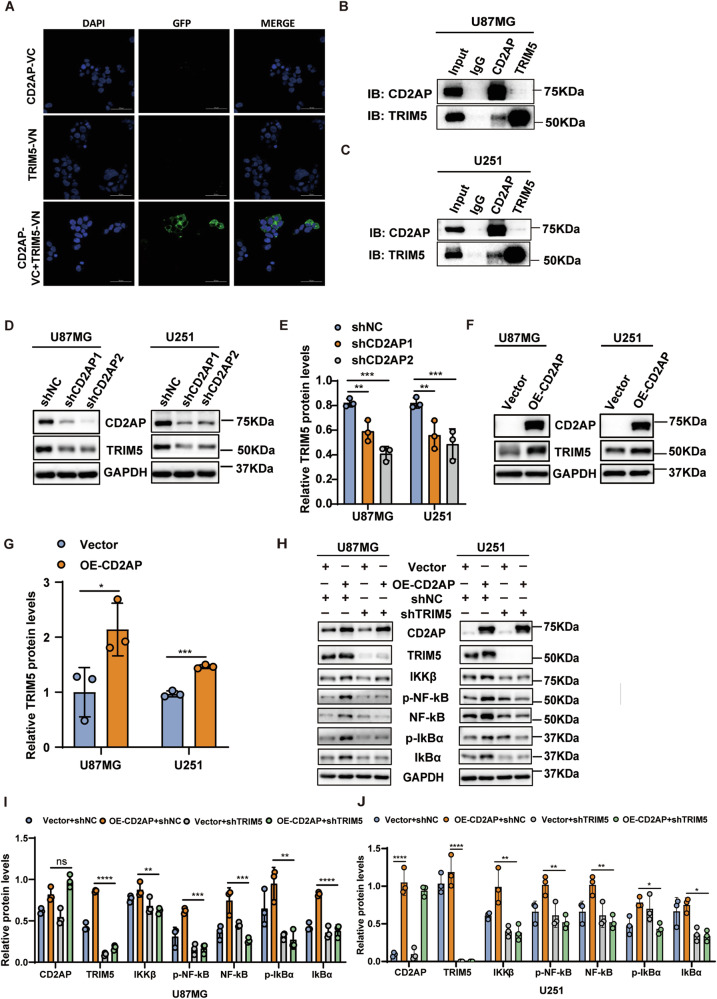
TRIM5 is required for CD2AP-mediated NF-kB activation. **A** HEK293T cells were transfected with TRIM5 carrying the N-terminus of Venus (TRIM5-VN) and CD2AP carrying the C-terminus of Venus (CD2AP-VC) individually or collectively for BiFC assay. Green signals indicate the interaction between TRIM5-VN and CD2AP-VC. Equal amounts of U87MG (**B**) and U251 (**C**) cell lysates were immunoprecipitated and then immunoblotted with anti-CD2AP and anti-TRIM5 antibodies. The TRIM5 protein in U87MG and U251 cells with CD2AP knockdown (**D**, **E**) or CD2AP overexpression (**F**, **G**) were analyzed by western blotting (**D**, **F**) and quantified for comparison (**E**, **G**). One-way ANOVA with Tukey’s post hoc test for (**E**) and Unpaired *t*-test for (**G**), *n* = 3 per group. U87MG and U251 cells with stable CD2AP overexpression were transfected with TRIM5 shRNA (shTRIM5) or control shRNA (shNC) and equal amounts of protein lysates were subjected to western blotting (**H**) and quantification comparison (**I**, **J**) for NF-kB signaling-related proteins. One-way ANOVA with Tukey’s post hoc test, *n* = 3 per group. Data represent mean ± SEM, *P < 0.05, **P < 0.01, ***P < 0.001, ****P < 0.0001, ns not significant.

To further determine how CD2AP regulates the NF-κB signaling, we used an CD2AP antibody and IgG to immunoprecipitate proteins in U87MG cells (Supplementary Fig. S5A) and then analyzed immunoprecipitated proteins by ion mobility mass spectrometry. We identified 2397 proteins immunoprecipitated by the CD2AP antibody and 2390 proteins immunoprecipitated by IgG (Supplementary Table S1). However, there were 2035 proteins immunoprecipitated by both the CD2AP antibody and IgG (Supplementary Fig. S5B). Among the 362 proteins specifically immunoprecipitated by the CD2AP antibody but not by IgG, Venn analysis revealed that only one protein, TRIM5, was also involved in the NF-κB signaling (Supplementary Fig. S5B, C). We further noticed that CD2AP and TRIM5 expressions in GBM samples were positively correlated using data from the GEPIA, CGGA, and Gravendeel datasets, though the coefficient values ranged between 0.489-0.65, implicating a moderate correlation between them (Supplementary Fig. S5D–F). To evaluate whether CD2AP interacts with TRIM5, we performed a bimolecular fluorescence complementation (BiFC) assay based on the fluorescent protein Venus [34]. We fused TRIM5 and CD2AP with the N- (VN) and C-terminal (VC) fragments of Venus, respectively. When TRIM5-VN and CD2AP-VC were expressed together but not individually, fluorescence was detected (Fig. 6A), suggesting an interaction between the two proteins. When CD2AP and TRIM5 were co-expressed in HEK 293 T cells, coimmunoprecipitation assays also revealed an interaction between them (Supplementary Fig. 5G). The interaction between endogenous CD2AP and TRIM5 was further confirmed by coimmunoprecipitation assays in GBM cells (Fig. 6B, C).

Moreover, we found that CD2AP knockdown reduced TRIM5 protein levels (Fig. 6D, E), whereas CD2AP overexpression increased TRIM5 protein levels (Fig. 6F, G). Importantly, in cells stably overexpressing CD2AP, downregulation of TRIM5 reversed the elevated NF-κB signaling activity by reducing protein levels of NF-κB/p65, p-NF-κB/p65, IKKβ, IκBα, and p-IκBα (Fig. 6H–J), though downregulation of TRIM5 had no significant effects on affecting NF-κB signaling proteins in control cells (Fig. 6H and Supplementary Fig. S5H, I). These results suggest that increased CD2AP promotes TRIM5 for activating NF-κB signaling, which can be reversed by TRIM5 knockdown.

### CD2AP mediates GBM resistance to temozolomide (TMZ)

**Fig. 7:**
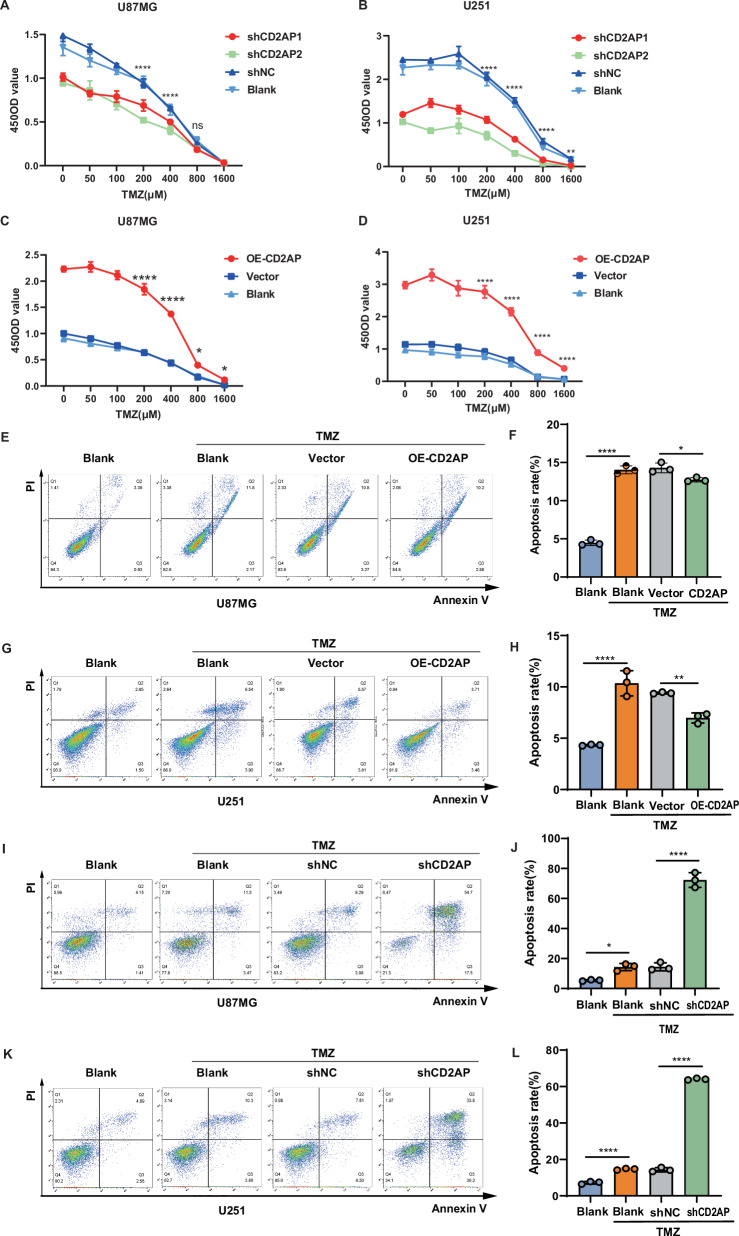
CD2AP promotes glioma cell resistance to TMZ. U87MG (**A**, **C**) and U251 (**B**, **D**) cells with CD2AP knockdown (**A**, **B**) or with CD2AP overexpression (**C**, **D**) and respective control cells were treated with indicated amounts of TMZ for 48 h, and then analyzed for cell proliferation. Two-way ANOVA with Sidak’s post hoc test, *n* = 4 per group for U87MG and *n* = 6 per group for U251. U87MG (**E**, **F**) and U251 (**G**, **H)** cells with stable CD2AP overexpression and control cells were treated with 200 μM TMZ for 48 h, and cell apoptosis was measured by flow cytometry (**E**, **G**) for apoptosis ratio comparison (**F**, **H**). One-way ANOVA with Tukey’s post hoc test, *n* = 3 per group. U87MG (**I**, **J**) and U251 (**K**, **L**) cells with stable CD2AP knockdown and control cells were treated with 200 μM TMZ for 48 h, and cell apoptosis was measured by flow cytometry (**I**, **K**) for apoptosis ratio comparison (**J**, **L**). One-way ANOVA with Tukey’s post hoc test, *n* = 3 per group. Data represent mean ± SEM, *P < 0.05, **P < 0.01, ****P < 0.0001, ns not significant.

TMZ resistance is a problem in GBM treatment [35, 36]. To investigate whether CD2AP is involved in TMZ resistance in GBM, we treated U87MG and U251 cells with CD2AP knockdown or CD2AP overexpression with different concentrations of TMZ. TMZ treatment dose-dependently reduced proliferation in all groups of cells (Fig. 7A–D). However, the proliferation of cells with CD2AP knockdown was more susceptible to TMZ treatment when compared to that of controls (Fig. 7A, B), whereas the proliferation of cells with CD2AP overexpression was more resistant to TMZ treatment when compared to that of controls (Fig. 7C, D). Moreover, TMZ treatment resulted in increased apoptosis in blank cells (Fig. 7E–L). However, cells with CD2AP overexpression exhibited less apoptosis than control cells in response to TMZ treatment (Fig. 7E–H). In contrast, cells with CD2AP knockdown showed more apoptosis than control cells in response to TMZ treatment (Fig. 7I–L). We also analyzed apoptosis-related proteins by western blot. The results confirmed that TMZ treatment dramatically promoted apoptosis, which were attenuated by CD2AP overexpression (Supplementary Fig. 6A–C) but potentiated by CD2AP knockdown (Supplementary Fig. 6D–F). These results indicate that CD2AP can mediate TMZ resistance in GBM cells.

## Discussion

CD2AP was originally identified as a molecule implicated in formation of the specialized junction between a T cell and an antigen-presenting cell [37]. Several studies have suggested that CD2AP was associated with but exhibited different functions in various malignant tumors [10, 14, 15]. One study found that CD2AP interacted with TKS4 to form a scaffolding protein complex involving in tumor development via regulating the epithelial–mesenchymal transition process in colon cancer [10], whereas another study showed that CD2AP could inhibit gastric cancer metastasis by promoting cellular adhesion and cytoskeleton assembly [14]. The expression pattern of CD2AP was also altered in several types of kidney tumors [15], implicating its involvement in kidney tumors. Nevertheless, whether and how CD2AP participates in other types of tumors including GBM remain unknown. In the present study, we identified a novel role of CD2AP in GBM. Through analysis of the transcriptome expression profiles of public databases and study on available clinical samples and GBM cell lines, we found that CD2AP expression was significantly upregulated in GBM samples compared to non-tumor and low-grade glioma samples. Furthermore, we found that high CD2AP expression was associated with poor survival rate of glioma patients. To study the biological function of CD2AP in GBM, we generated GBM cell lines with stable CD2AP overexpression or knockdown. CD2AP overexpression significantly promoted proliferation, colony formation, migration, and invasion of GBM cells and decreased their apoptosis. GBM cells with stable CD2AP overexpression also formed larger tumors than control cells when xenografted into the brain of mice, resulting in reduced mouse survival rate. While CD2AP knockdown reduced the malignancy of GBM cells both in vitro and in vivo. Moreover, GBM cells with CD2AP overexpression were more resistant, whereas GBM cells with CD2AP knockdown were more susceptible to TMZ. Together, these results indicate that CD2AP is a potential biomarker and therapeutic target for GBM.

RNA-seq GSEA profiles revealed a positive correlation between CD2AP expression and the NF-κB signaling in GBM. Prior studies have reported that aberrant activation of NF-κB signaling promotes tumor progression including GBM [21]. For example, tripartite motif-containing 25 (TRIM25) facilitates immunosuppression and inhibits glioma apoptosis via activating NF-κB [38]. Chitinase-3 like-protein-1 (CHI3L1) binds to actinin alpha 4 (ACTN4) and NF-κB1 and enhances the NF-κB signaling by promoting the NF-κB subunit nuclear translocation to facilitate glioma progression [32]. LINC01057 interacts with IKKα and maintains IKKα nucleus localization, leading to NF-κB activation and GBM progression [39]. Fos-like antigen 1 (FOSL1) promotes proneural to mesenchymal transition (PMT) in glioblastoma stem cells (GSCs) through NF-κB signaling pathway [40]. T cell immunoglobulin domain and mucin domain protein 3 (TIM-3) regulates IL6 expression via activation of NF-κB signaling, mediating immune escape of GBM [41]. Receptor-interacting protein 2 (RIP2) enhances GBM cell resistance to TMZ through activation of NF-κB and upregulation of MGMT expression [42]. Therefore, we speculated that CD2AP could promote the progression of GBM also by activating NF-κB signaling. As expected, CD2AP overexpression promoted, whereas CD2AP knockdown reduced the activity of NF-κB. In addition, CD2AP overexpression facilitated nuclear translocation of phosphorylated NF-κB and transcription of downstream target genes. Moreover, we found that inhibiting the NF-κB activity reversed the elevated malignancy of GBM upon CD2AP overexpression. Thus, our results demonstrate that CD2AP exerts an oncogenic function in GBM cells through enhancing the NF-κB signaling.

Because it was reported that CD2AP bound CAPZA1 to promote intercellular adhesion and to influence cell cytoskeleton in gastric cancer [14], there is a possibility that CD2AP also interacts with certain protein(s) in the NF-kB signaling to regulate the NF-kB activity. Therefore, we performed IP mass spectrometry assay to identify CD2AP-interacting proteins and found one of them, TRIM5, has been associated with NF-κB signaling. TRIM5 was initially identified as a host antiviral protein and later studies found that human TRIM5 could promote NF-kB activity [18–20]. Another study noticed that TRIM5 expression was increased in glioma when compared to normal tissues [43]. Therefore, it is possible that CD2AP activates NF-κB signaling by interacting with TRIM5. We confirmed the interaction between CD2AP and TRIM5 using co-IP and BiFC assays. Importantly, we found that overexpression and knockdown of CD2AP increased and decreased TRIM5 levels, respectively, and that downregulation of TRIM5 reversed the activated NF-κB signaling in GBM cells with CD2AP overexpression. Together, these results indicate that elevated CD2AP promotes the NF-κB signaling through interacting with TRIM5 and increasing its levels.

In summary, this study identifies a novel oncogenic role of CD2AP in GBM cells. We showed that CD2AP expression was significantly increased in GBM and that high CD2AP expression was correlated with a poor prognosis. Moreover, we demonstrated that CD2AP overexpression promoted GBM malignant behavior through interacting with TRIM5 to enhance the NF-κB signaling. Therefore, CD2AP may represent a novel biomarker for the evaluation of GBM patient prognosis and the CD2AP-TRIM5-NF-κB axis may have therapeutic value for GBM treatment.

## Supplementary information

Supplementary Figures S1-S6

Supplementary Table S1

Supplementary raw gel images

## Data Availability

The datasets used and/or analyzed in the current study are available from the corresponding authors upon reasonable request.
