# Supplementary material for: CD2AP promotes the progression of glioblastoma multiforme via TRIM5-mediated NF-kB signaling
Source: Cell Death Dis. 2024 Oct 1;15(10):722. doi: 10.1038/s41419-024-07094-7 (PMC11445578; doi:10.1038/s41419-024-07094-7)
Supplement: Supplementary file 1 — Supplementary Figures S1-S6 [file 41419_2024_7094_MOESM1_ESM.docx]

**Supplementary materials**

**CD2AP promotes the progression of glioblastoma multiforme via TRIM5-mediated NF-kB signaling**

Liang Zhang et al.

**Supplementary Figures S1-S6**

**
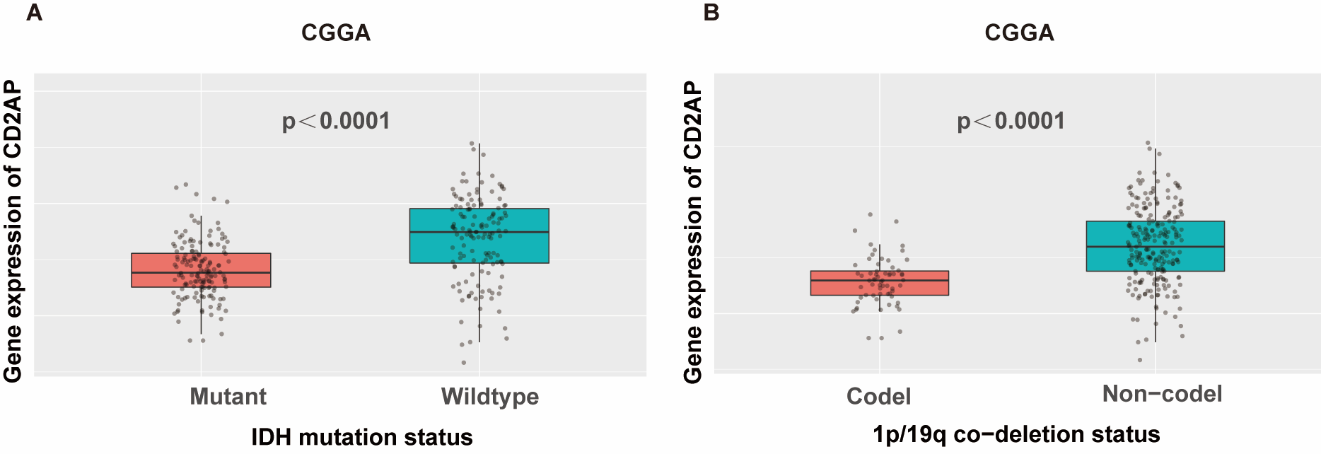
**

**Supplementary Figure S1. CD2AP expression is decreased in GBM samples with IDH mutations and 1/19q codeletions.** (A) CD2AP expression comparison between GBM samples with or without IDH mutations. Unpaired t test, n = 172 for Mutant and n=145 for Wildtype. (B) CD2AP expression comparison between GBM samples with or without 1/19q codeletions (Codel). Unpaired t test, n = 67 for Codel and n=250 for Non-codel. Data represent mean ± SEM.


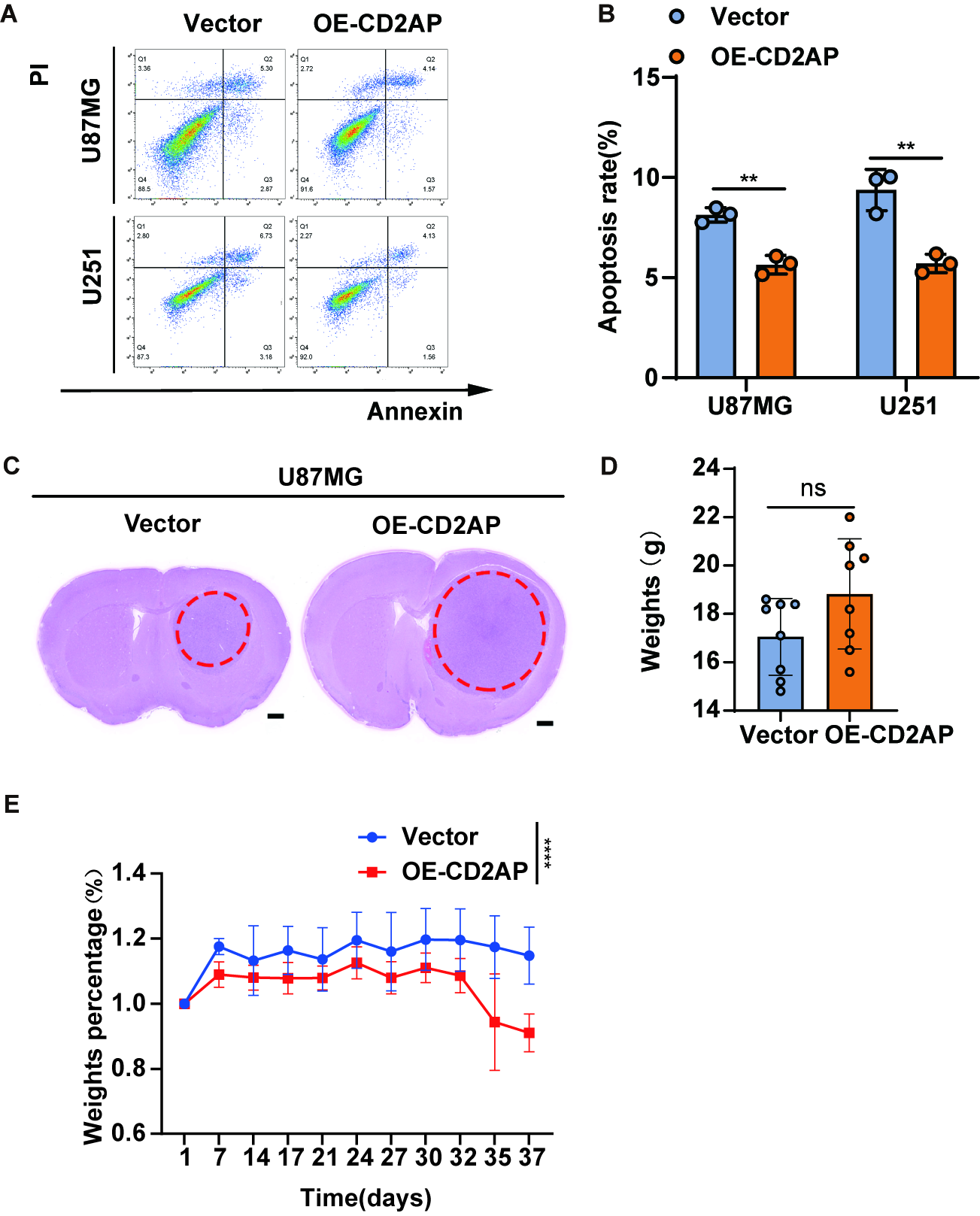


**Supplementary Figure S2. CD2AP overexpression reduces cell apoptosis and promotes tumor formation.** (A,B) U87MG and U251 cells with stable CD2AP overexpression (OE-CD2AP) were measured by flow cytometry (A) for apoptosis ratio comparison (B). Unpaired t test, n = 3 per group. (C) Representative H&E staining showing tumor regions in mice xenografted with U87MG cells stably overexpressing CD2AP or control cells. Scale bars: 500 μm. (D) Body weight comparison before mice were xenografted with U87MG cells stably overexpressing CD2AP or control cells. Unpaired t test, n = 8 per group. (E) Body weight change of mice xenografted with U87MG cells stably overexpressing CD2AP or control cells. Two-way ANOVA with Sidak’s post hoc test, n = 8 per group. Data represent mean ± SEM, **P < 0.01, ****P <0.0001, ns: not significant.


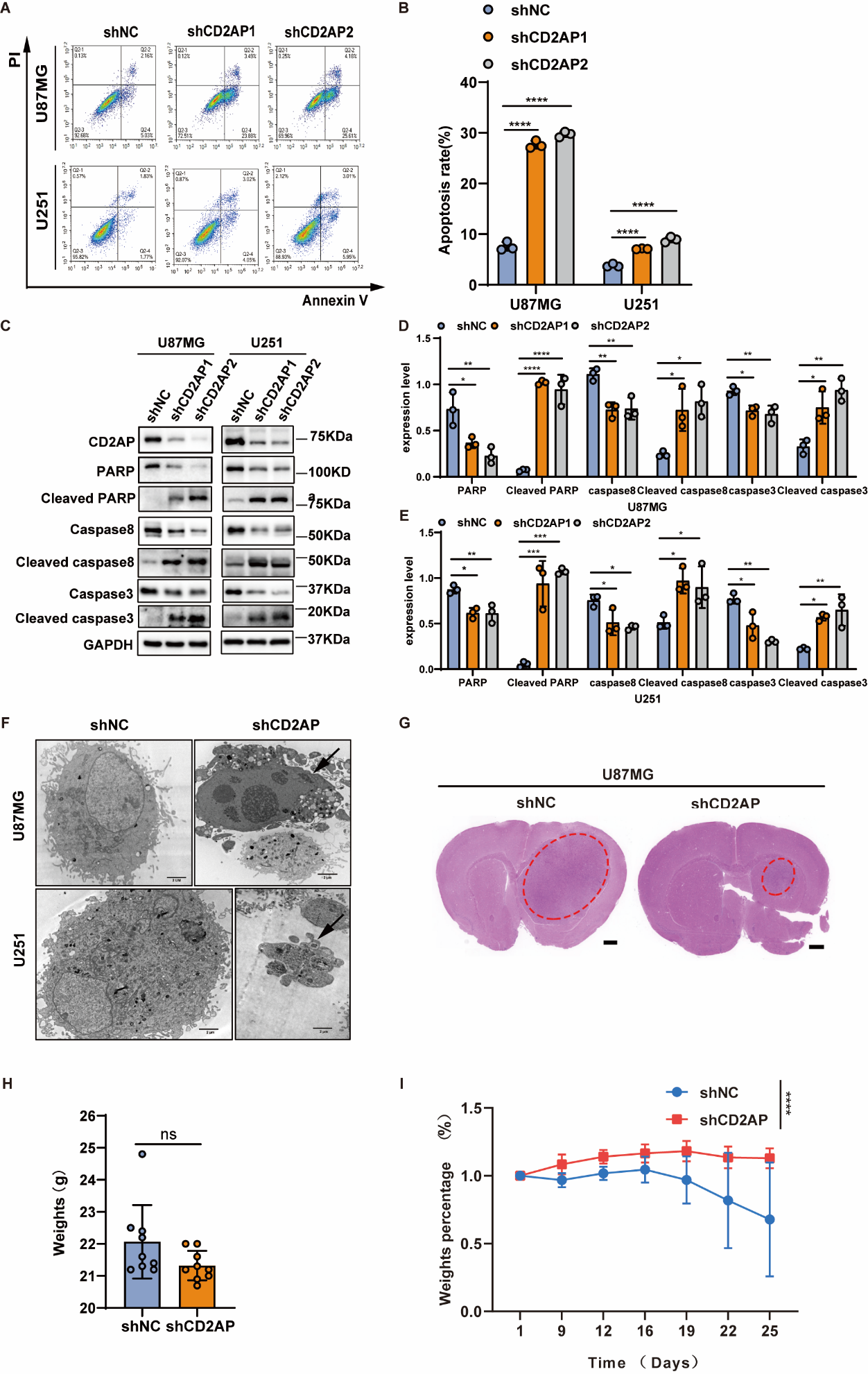


**Supplementary Figure S3. CD2AP knockdown promotes cell apoptosis and reduces tumor formation.** (A,B) U87MG and U251 cells with CD2AP knockdown were measured by flow cytometry (A) for apoptosis ratio comparison (B). One-way ANOVA with Tukey’s post hoc test, n = 3 per group. (C-E) Equal amounts of cell lysates of U87MG and U251 cells with CD2AP knockdown were subjected to western blotting (C) and quantification analysis (D,E) for apoptosis-related proteins. One-way ANOVA with Tukey’s post hoc test, n = 3 per group. (F) Representative electron microscopy images showing apoptotic cell bodies (indicated by arrows) with CD2AP knockdown. 2.5K, scale bars: 2 μm. (G) Representative H&E staining showing tumor regions in mice xenografted with U87MG cells with CD2AP knockdown or control cells. Scale bars: 500 μm. (H) Body weight comparison before mice were xenografted with U87MG cells with CD2AP knockdown or control cells. Unpaired t test, n = 9 per group. (I) Body weight change of mice xenografted with U87MG cells with CD2AP knockdown or control cells. Two-way ANOVA with Sidak’s post hoc test, n = 9 per group. Data represent mean ± SEM, *P < 0.05, **P < 0.01, ***P < 0.001, ****P <0.0001, ns: not significant.


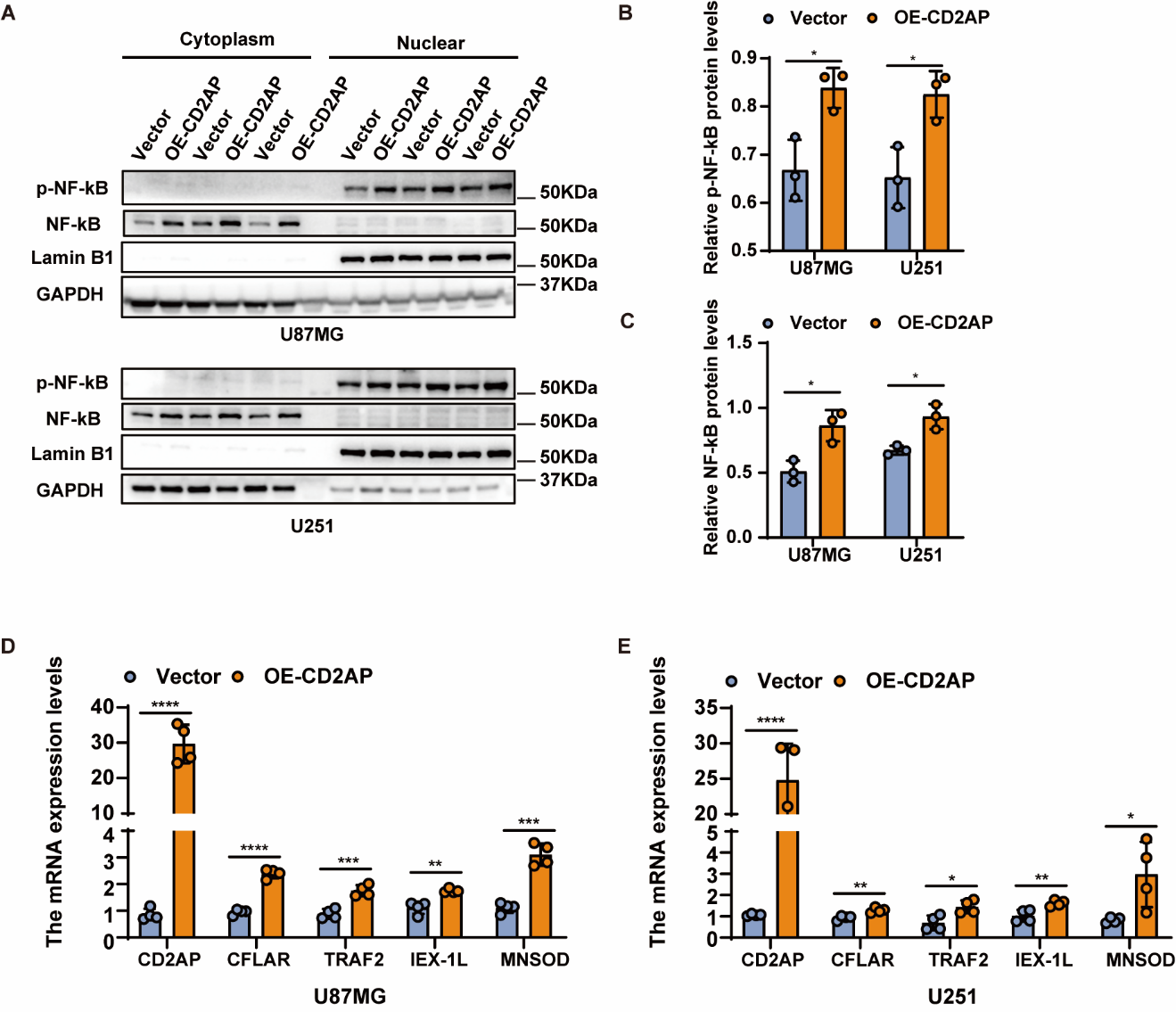


**Supplementary Figure S4. CD2AP overexpression promotes nuclear translocation of p-NF-κB and NF-κB downstream gene expression.** (A,B) After separating cytoplasmic and nuclear fractions of U87MG and U251 cells with CD2AP overexpression, equal amounts of protein lysates were subjected to western blotting (A) and quantification analyses (B) for p-NF-κB (site 536) in the nuclear fraction and NF-κB in the cytoplasmic fraction (C). Lamin B1 was used as a nuclear marker. Unpaired t test, n = 3 per group. (D,E) U87MG and U251cells were stably transduced with lentiviruses expressing CD2AP or control vector and the mRNA levels of CD2AP and several NF-κB downstream targets were studied for comparison. Unpaired t test, n = 4 per group. Data represent mean ± SEM, *P < 0.05, **P < 0.01, ***P＜0.001, ****P <0.0001.


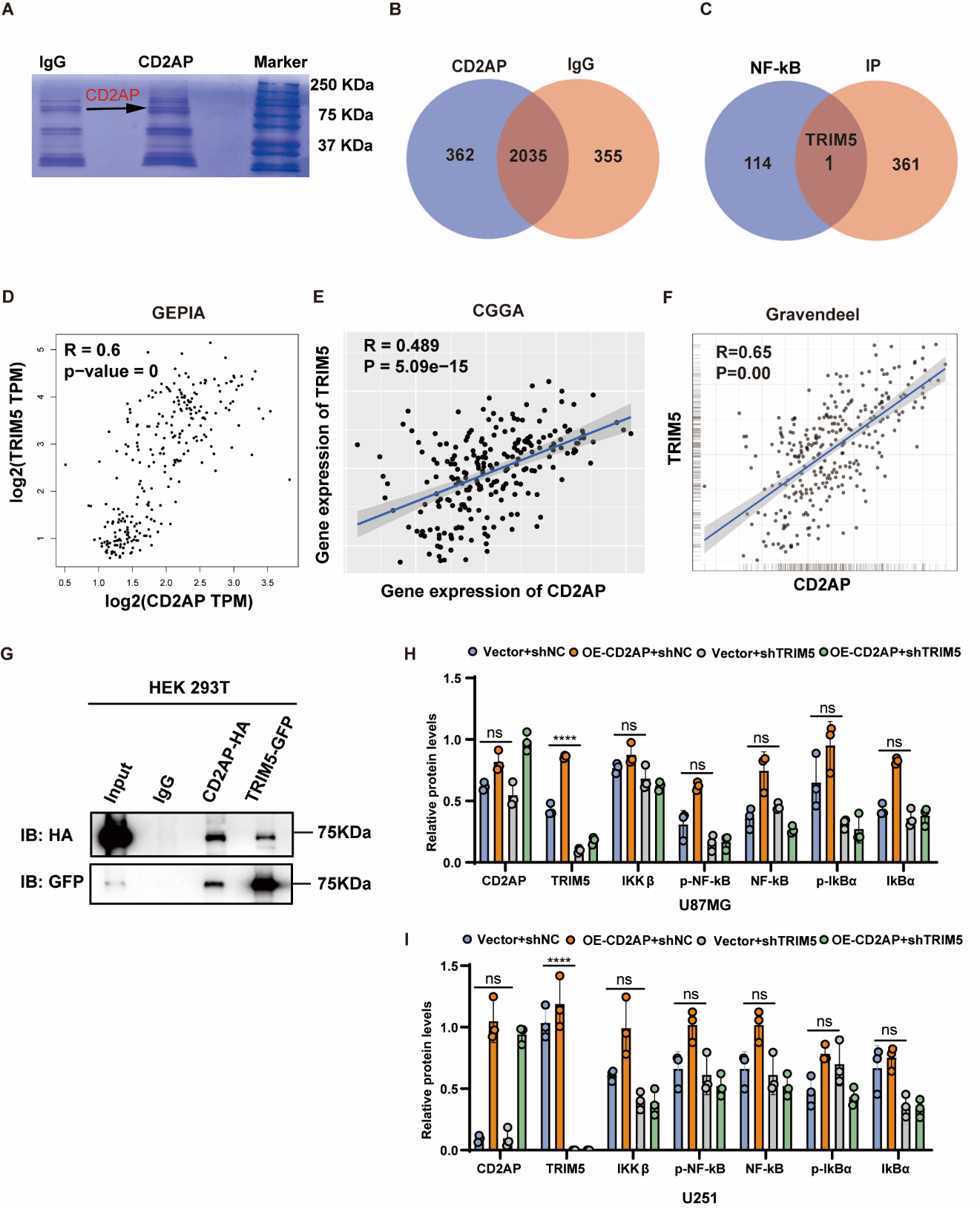


**Supplementary Figure S5. CD2AP interacts with and is correlated with TRIM5 in GBM.** (A) Equal amounts of U87MG cell protein lysates were immunoprecipitated with an anti-CD2AP antibody and IgG. Immunoprecipitated proteins were subjected to SDS-PAGE and Coomassie blue staining. (B) Venn diagram analysis of proteins immunoprecipitated by the anti-CD2AP antibody and by IgG. (C) Venn diagram analysis of NF-kB signaling-related proteins and proteins specifically immunoprecipitated by the anti-CD2AP antibody. (D-F) Pearson correlation analysis of CD2AP and TRIM5 based on data from the GEPIA (D), CGGA (E), and Gravendeel datasets (F). (G) HEK293T cells were co-transfected with CD2AP-HA and TRIM5-GFP vectors. Equal amounts of cell lysates were immunoprecipitated and then immunoblotted with anti-HA and anti-GFP antibodies. (H,I) U87MG (H) and U251 (I) cells with stable CD2AP overexpression or control vector were transfected with TRIM5 shRNA (shTRIM5) or control shRNA (shNC) and equal amounts of protein lysates were subjected to western blotting (shown in Figure 6H) and quantification comparison for NF-kB signaling-related proteins between Vector+shNC and Vector+shTRIM5. One-way ANOVA with Tukey’s post hoc test, n = 3 per group. Data represent mean ± SEM, ****P <0.0001, ns: not significant.


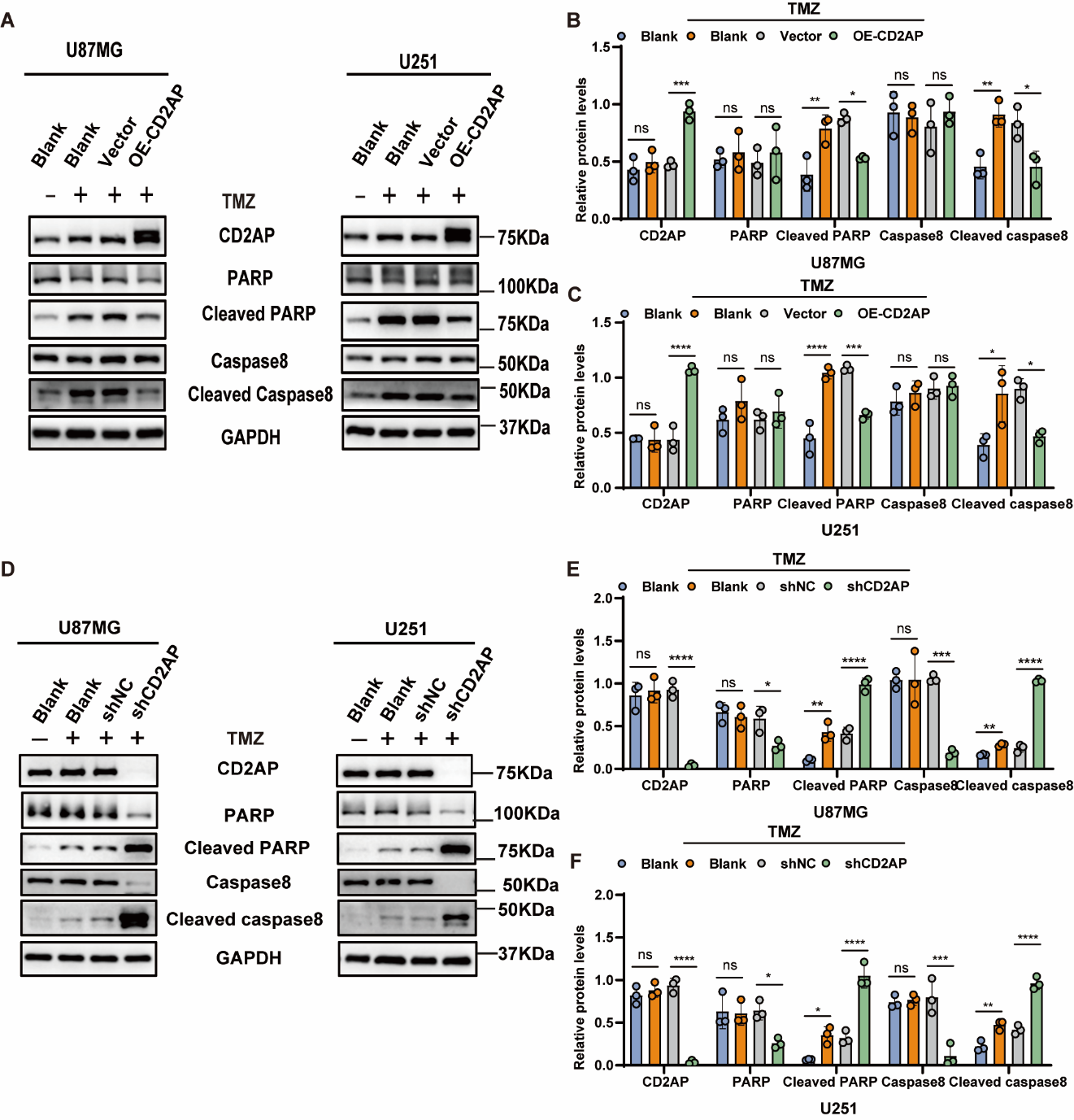


**Supplementary Figure S6. CD2AP promotes glioma cell resistance to TMZ.** (A-C) U87MG and U251 cells with stable CD2AP overexpression (OE-CD2AP) and control cells were treated with 200 μM TMZ for 48 h and equal amounts of protein lysates were subjected to western blotting (A) and quantification comparison (B,C) for apoptosis-related proteins. (D-F) U87MG and U251 cells with stable CD2AP knockdown and control cells were treated with 200 μM TMZ for 48 h and equal amounts of protein lysates were subjected to western blotting (D) and quantification comparison (E,F) for apoptosis-related proteins. One-way ANOVA with Tukey’s post hoc test, n = 3 per group. Data represent mean ± SEM, *P < 0.05, **P < 0.01, ***P＜0.001, ****P <0.0001, ns: not significant.
