# Supplementary material for: CD2AP promotes the progression of glioblastoma multiforme via TRIM5-mediated NF-kB signaling
Source: Cell Death Dis. 2024 Oct 1;15(10):722. doi: 10.1038/s41419-024-07094-7 (PMC11445578; doi:10.1038/s41419-024-07094-7)
Supplement: Supplementary file 3 — Supplementary raw gel images [file 41419_2024_7094_MOESM3_ESM.pdf]

Full unedited gel for Figure 1H

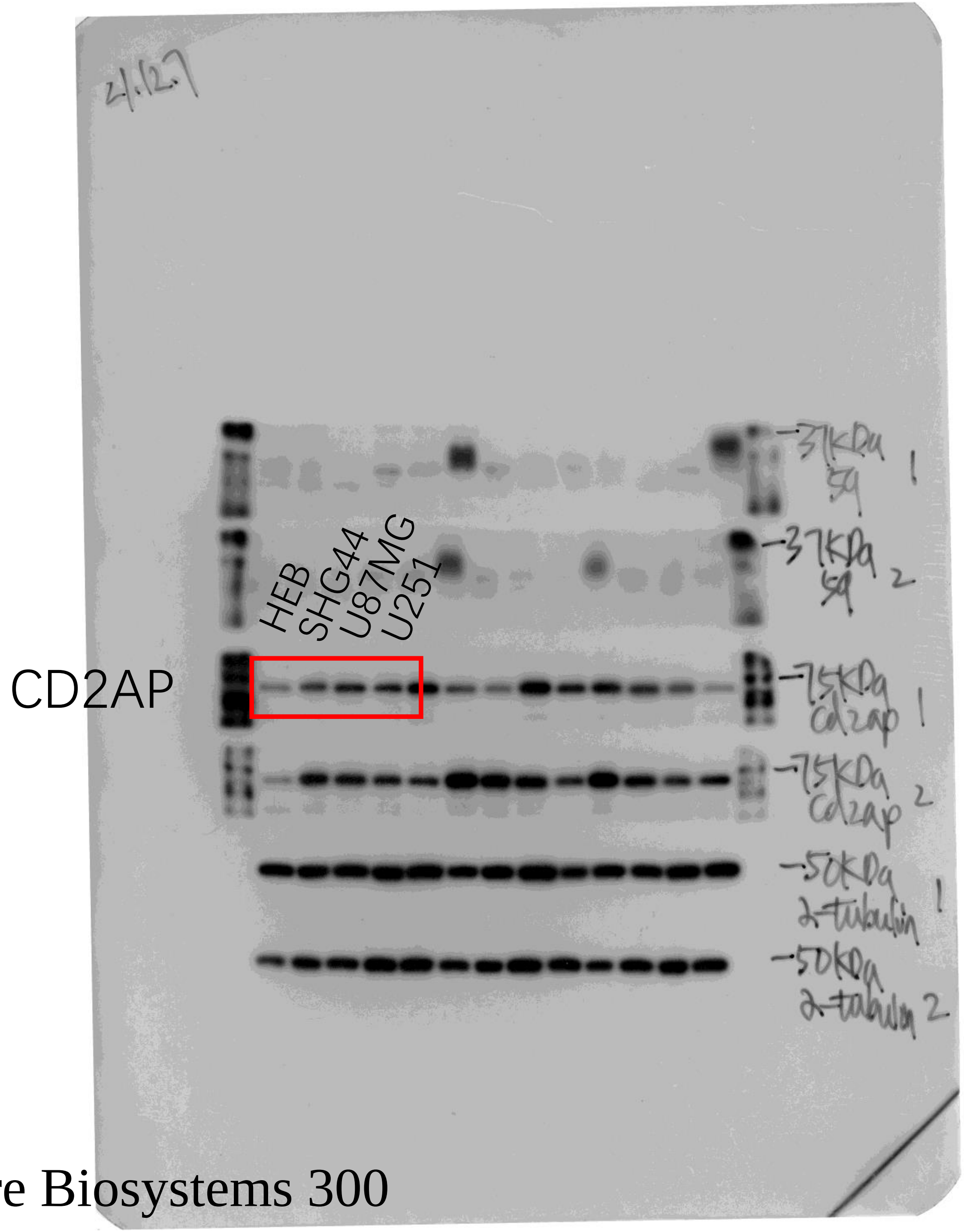

The membrane was imaged with Azure Biosystems 300

Full unedited gel for Figure 1H

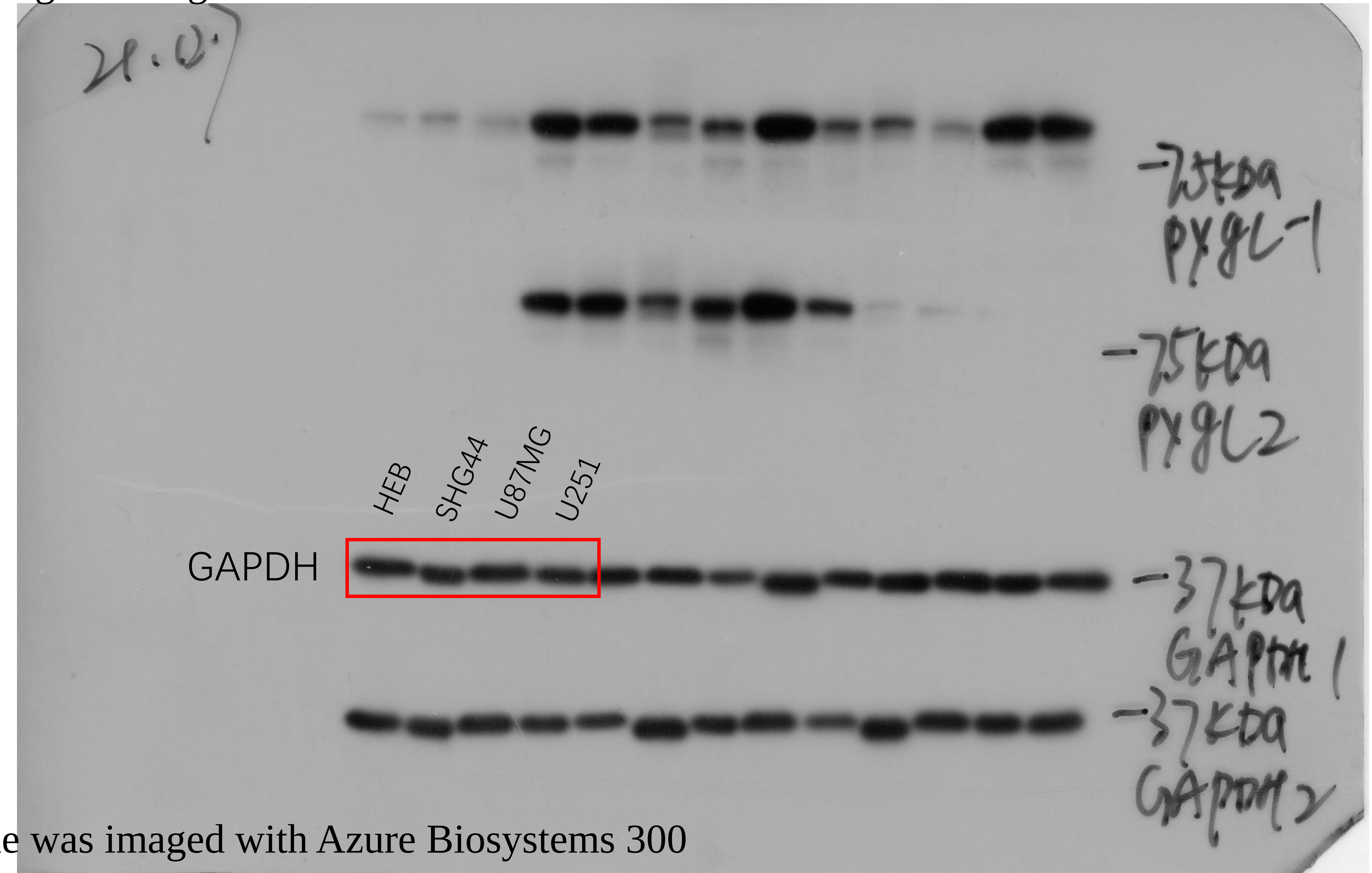

The membrane was imaged with Azure Biosystems 300

Full unedited gel for Figure 1J

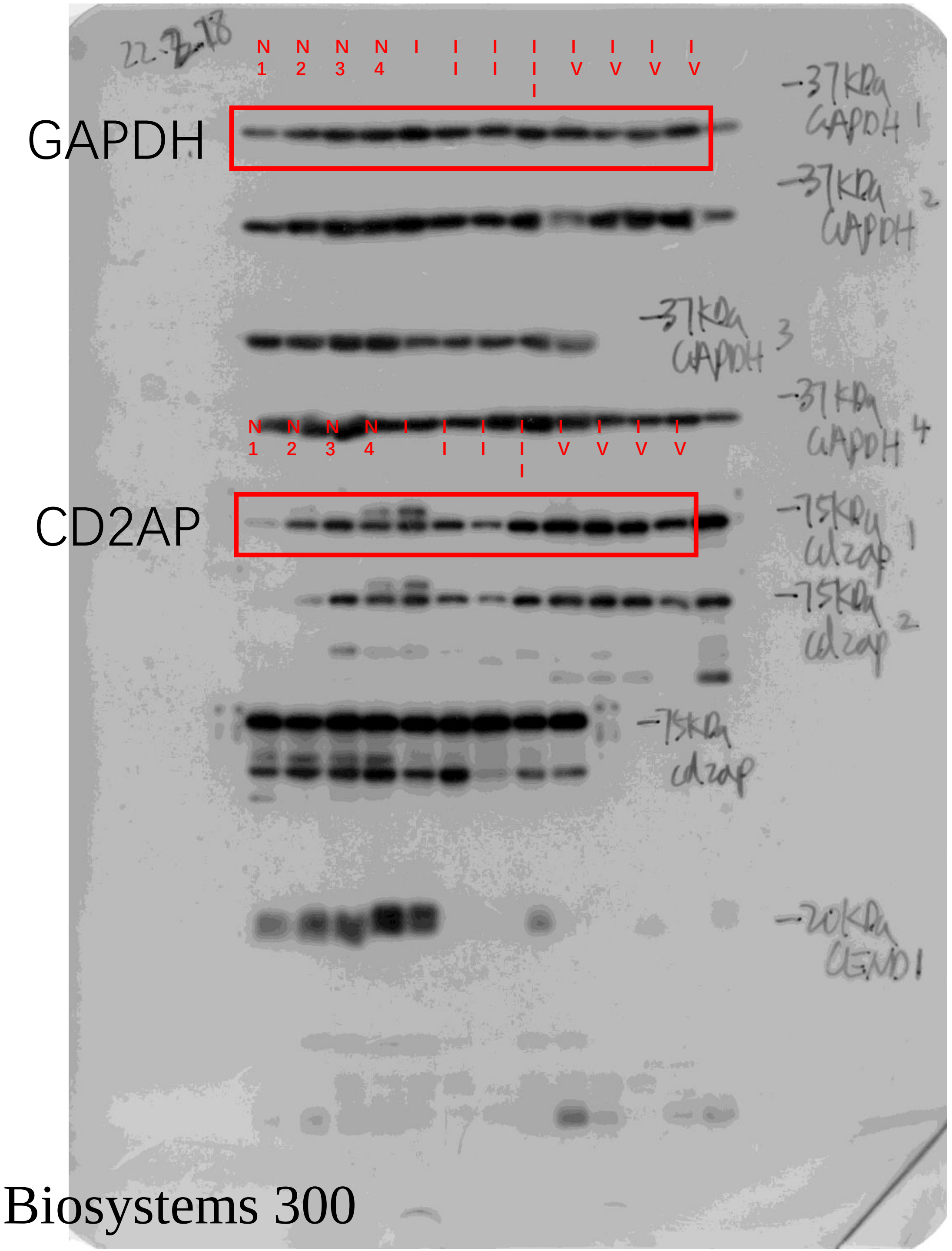

The membrane was imaged with Azure Biosystems 300

Full unedited gel for Figure 2B

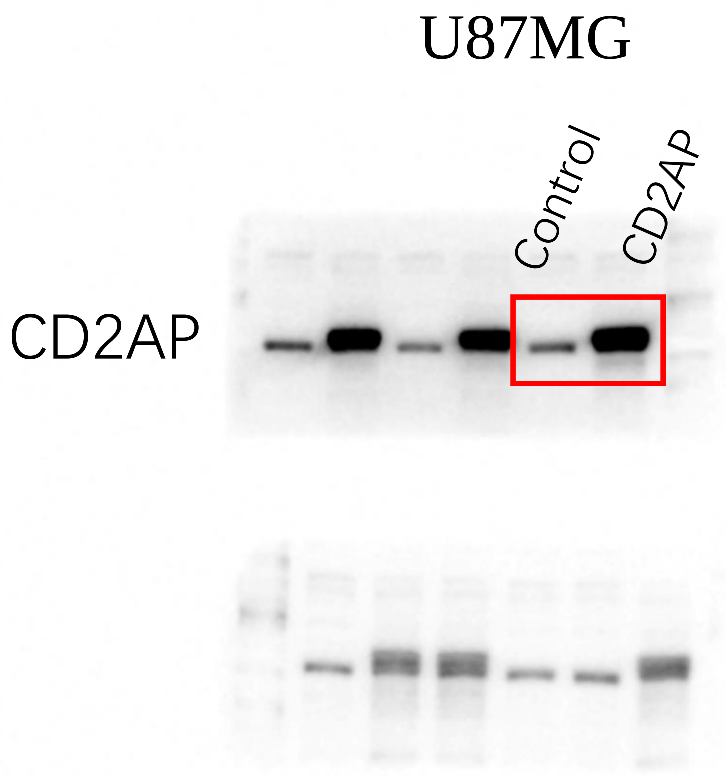

The membrane was imaged with Azure Biosystems 300

Full unedited gel for Figure 2B

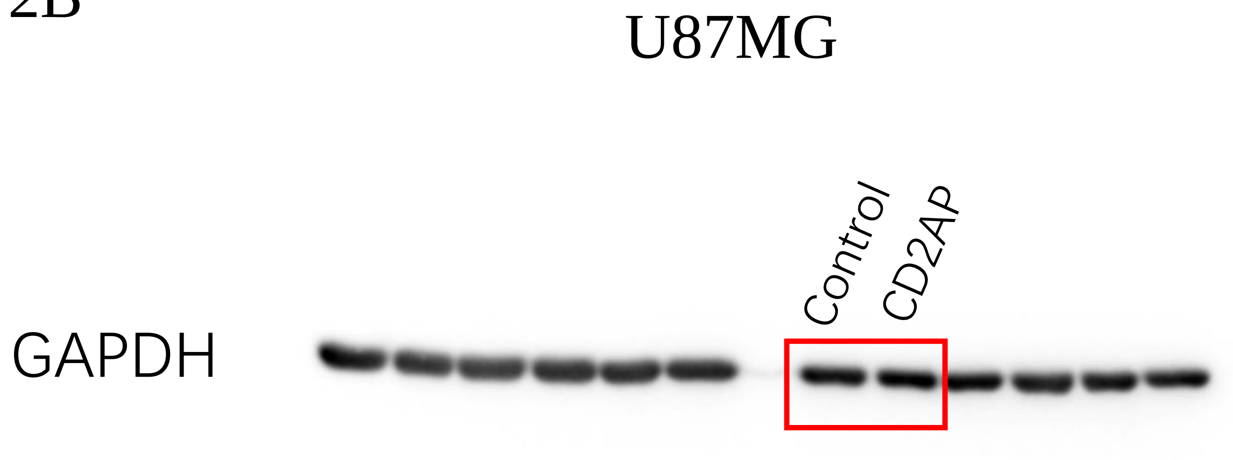

The membrane was imaged with Azure Biosystems 300

Full unedited gel for Figure 2B

U251

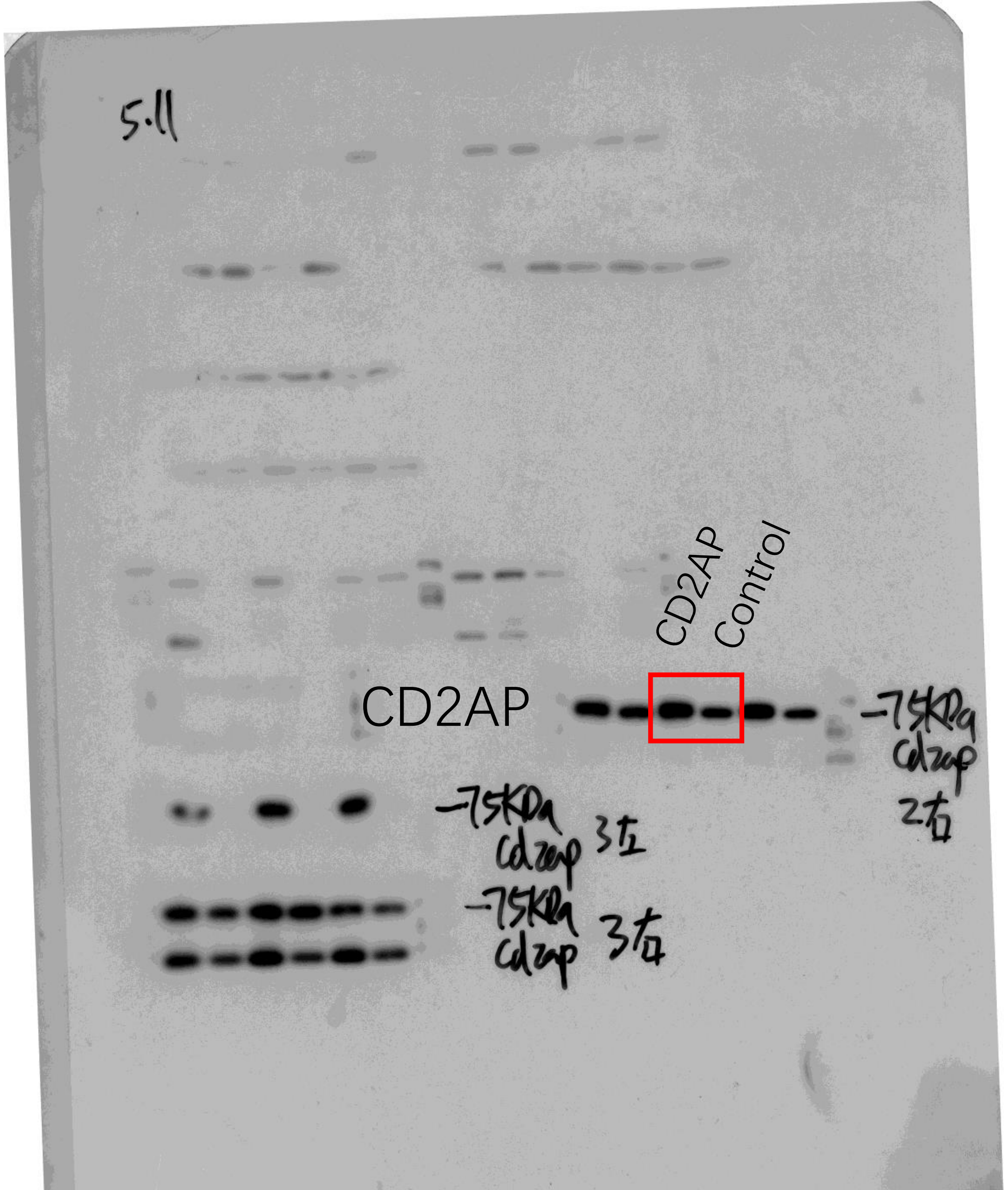

The membrane was imaged with Azure Biosystems 300

Full unedited gel for Figure 2B

U251

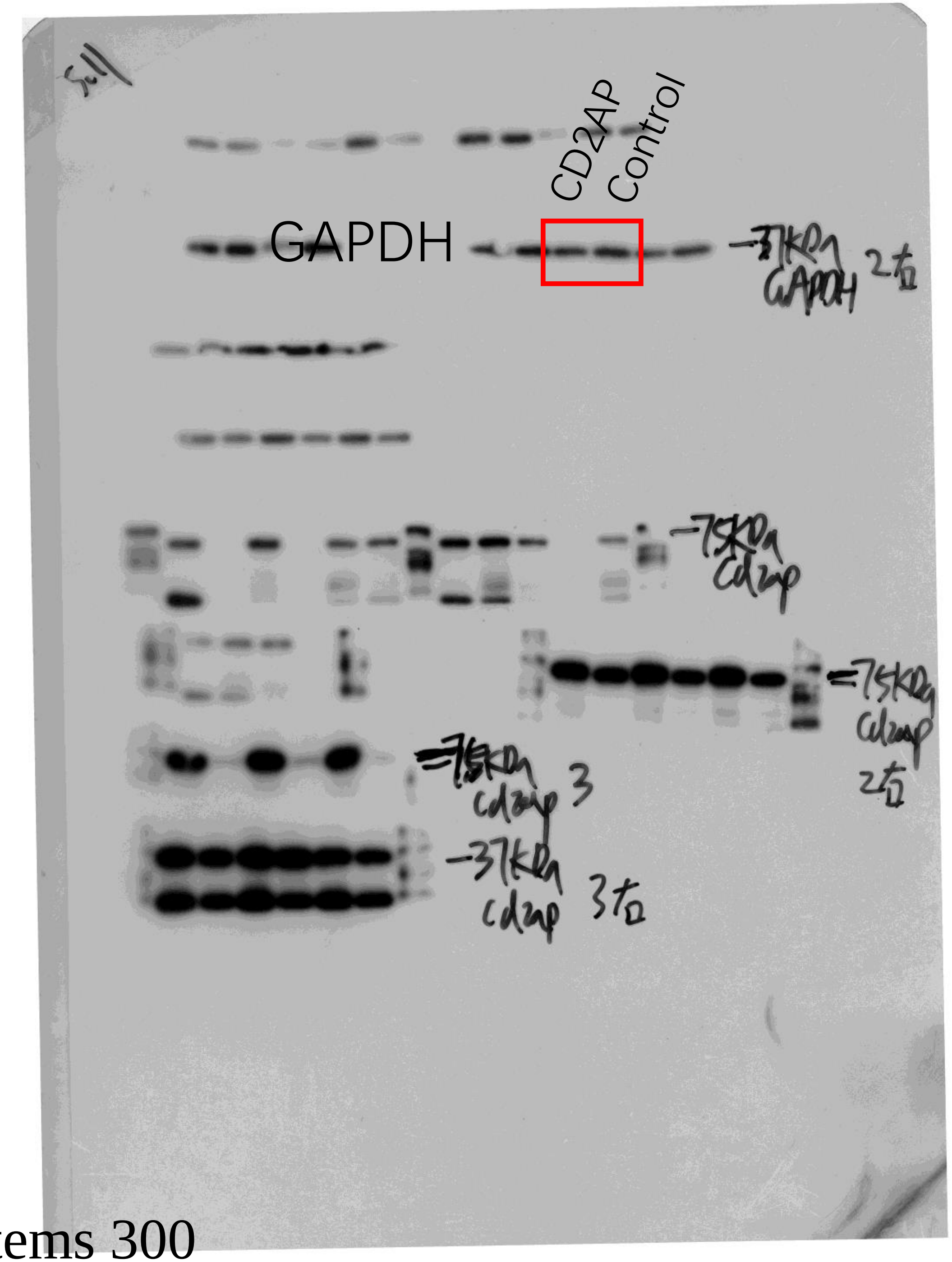

The membrane was imaged with Azure Biosystems 300

Full unedited gel for Figure 3B

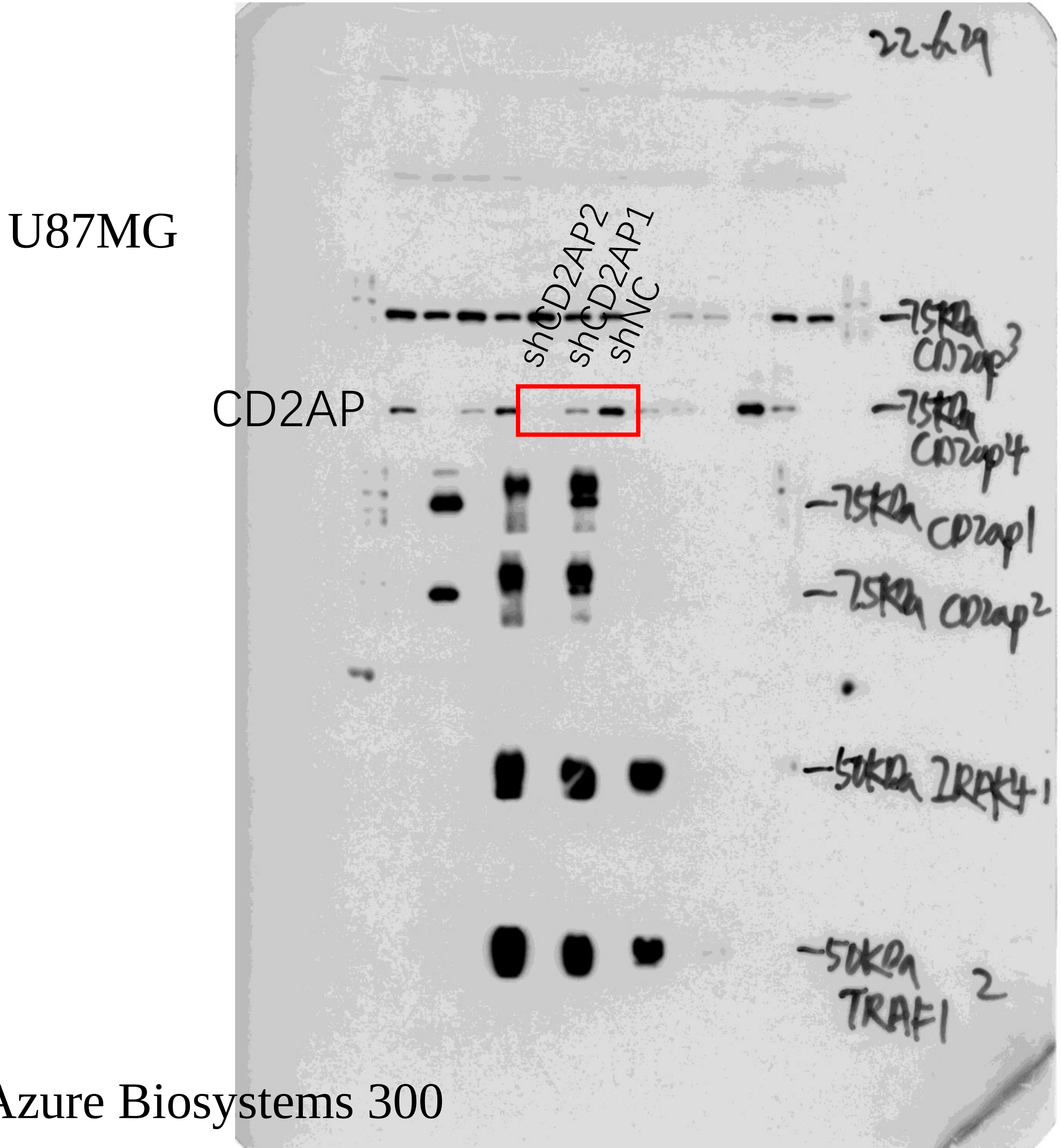

The membrane was imaged with Azure Biosystems 300

Full unedited gel for Figure 3B

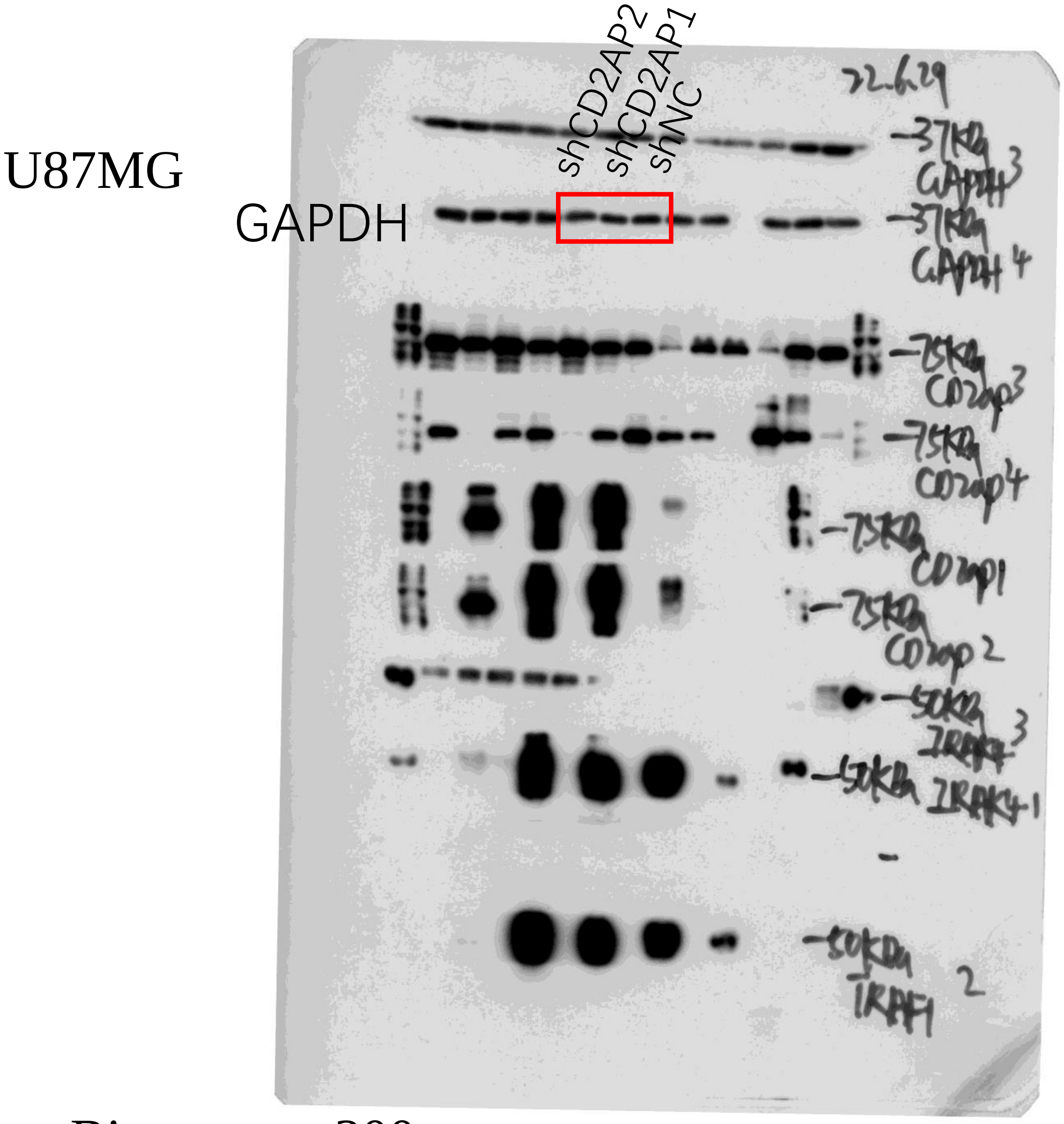

The membrane was imaged with Azure Biosystems 300

Full unedited gel for Figure 3B

U251

CD2AP

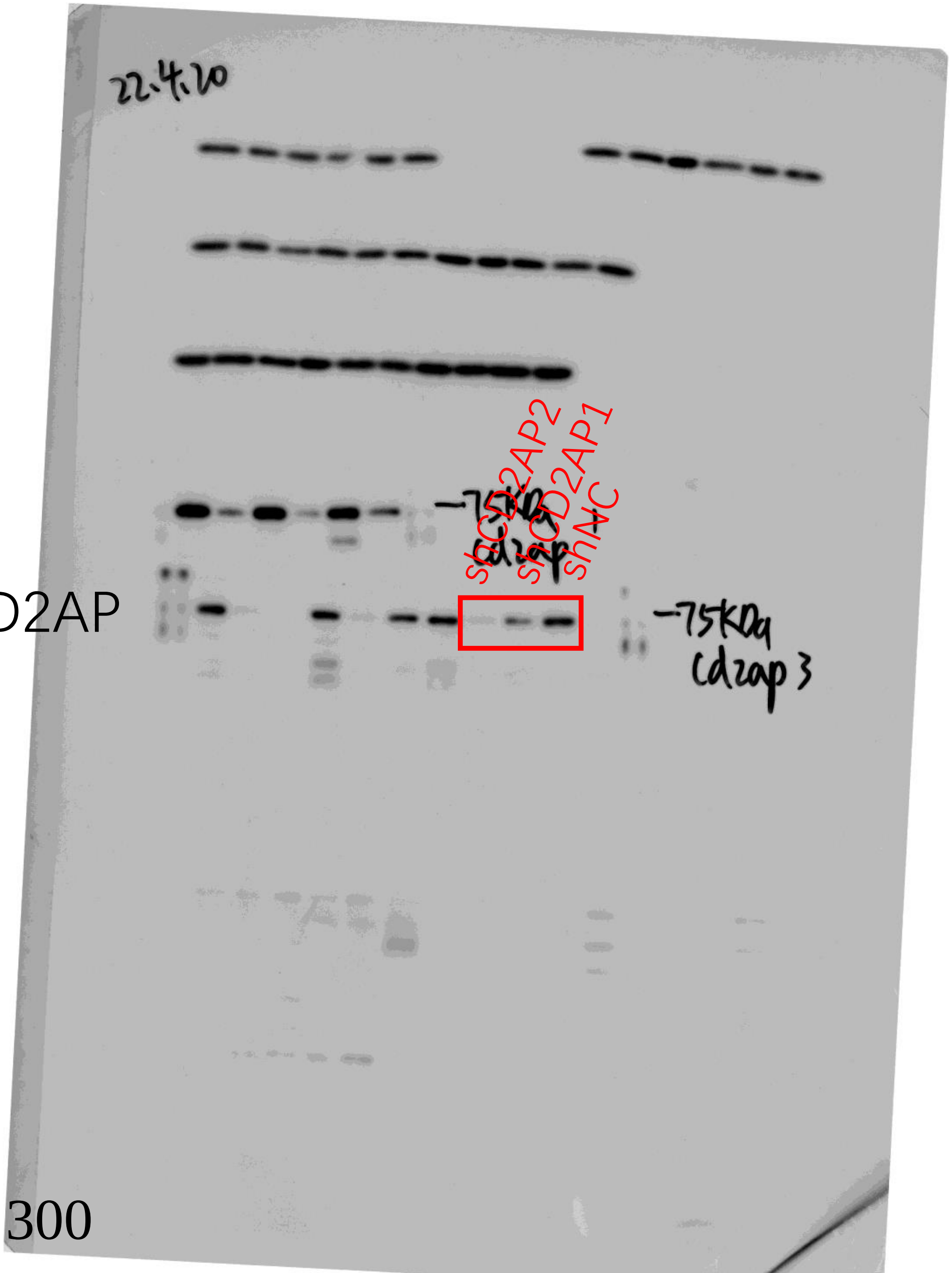

The membrane was imaged with Azure Biosystems 300

Full unedited gel for Figure 3B

U251

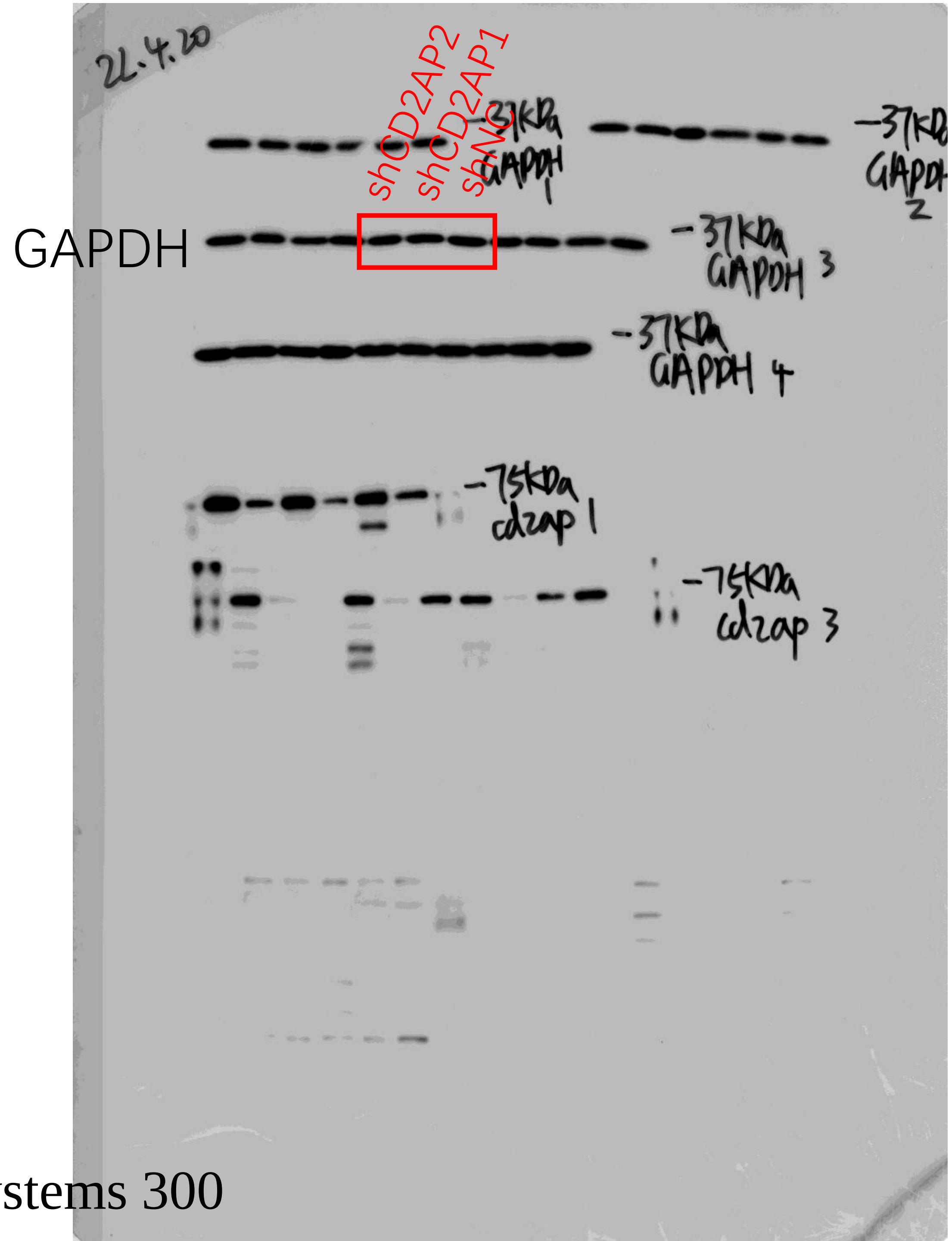

The membrane was imaged with Azure Biosystems 300

Full unedited gel for Figure 4D

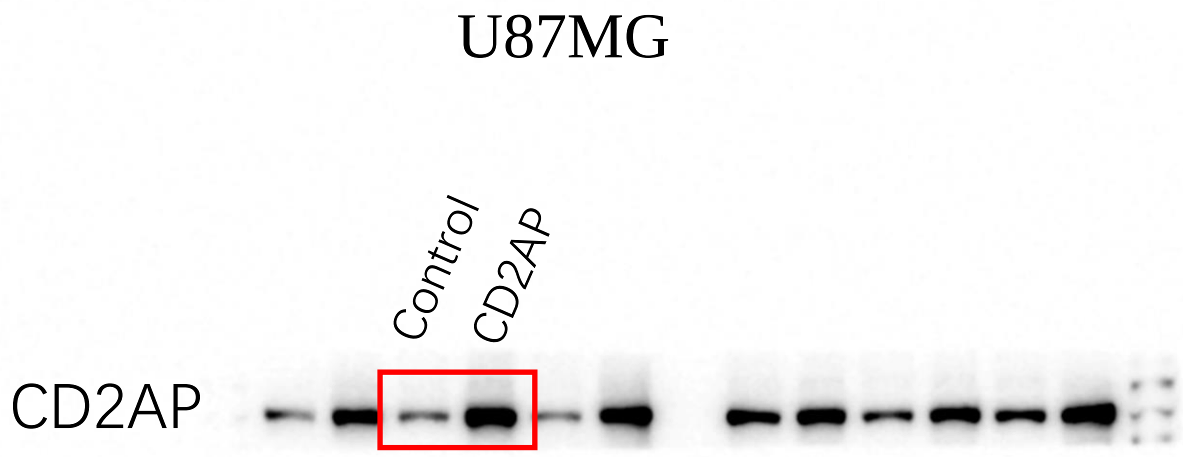

The membrane was imaged with Azure Biosystems 300

Full unedited gel for Figure 4D

U87MG

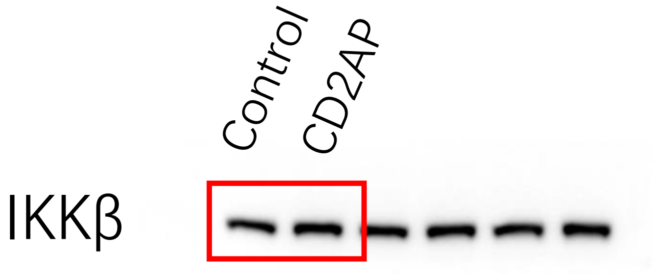

The membrane was imaged with Azure Biosystems 300

Full unedited gel for Figure 4D

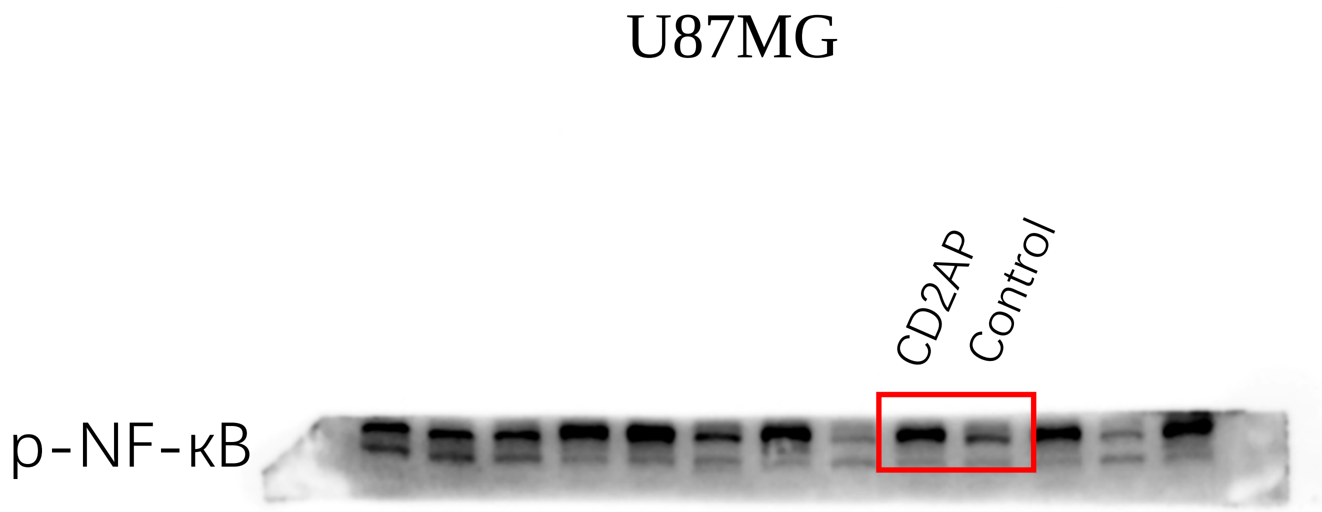

The membrane was imaged with Azure Biosystems 300

Full unedited gel for Figure 4D

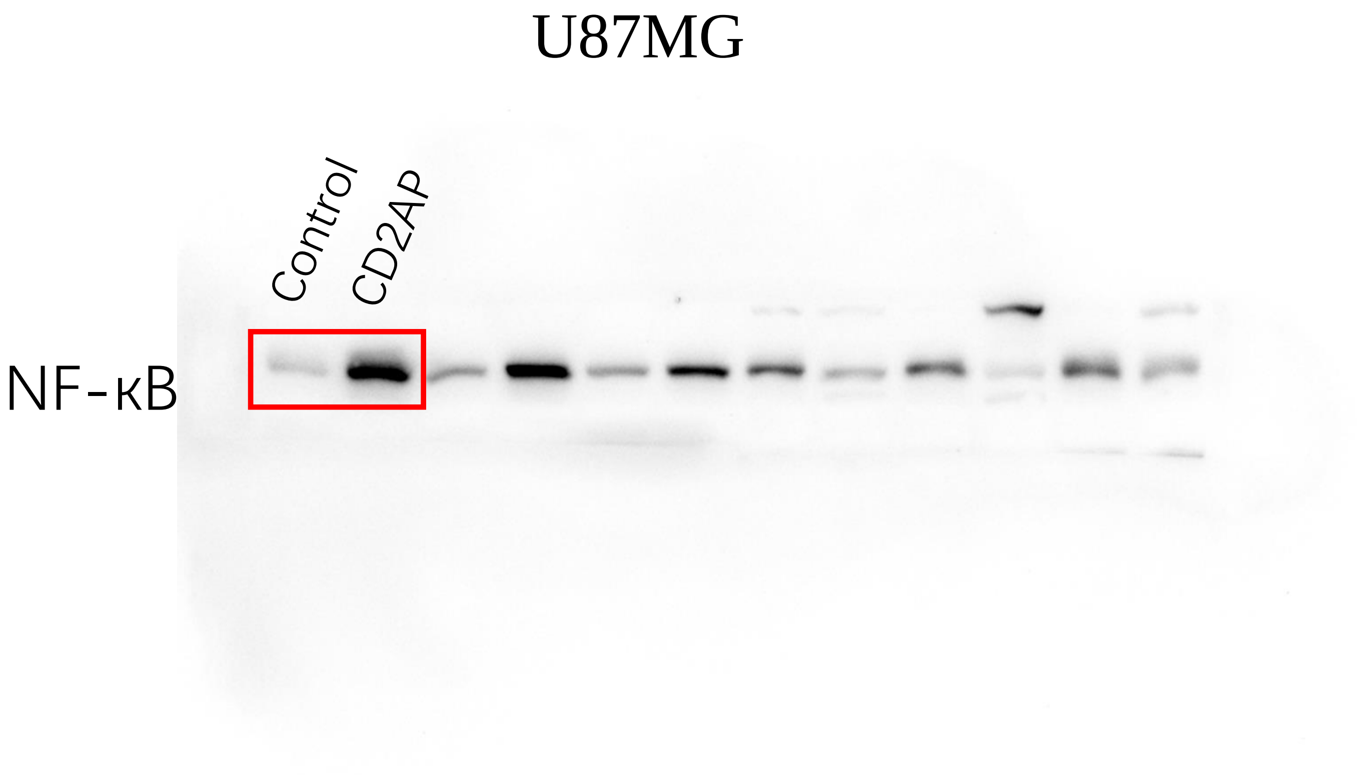

The membrane was imaged with Azure Biosystems 300

Full unedited gel for Figure 4D

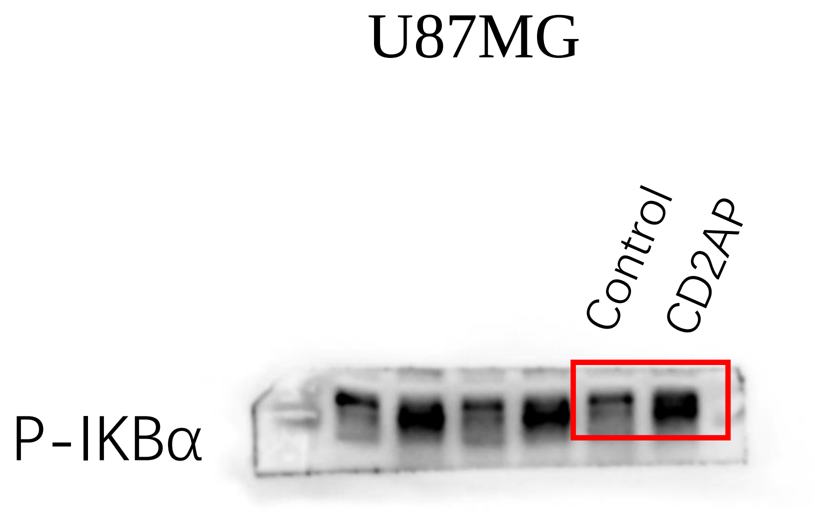

The membrane was imaged with Azure Biosystems 300

Full unedited gel for Figure 4D

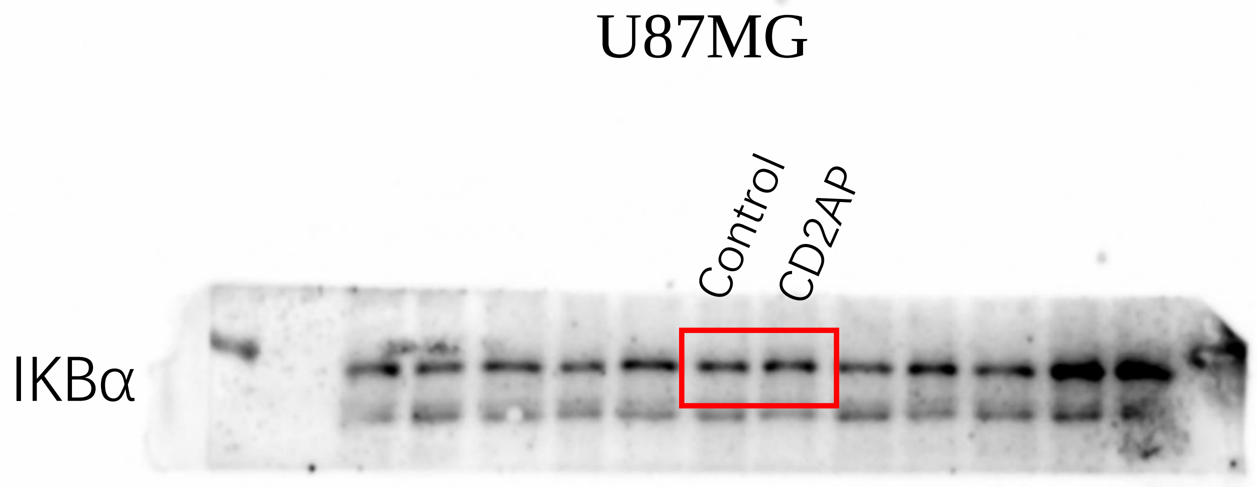

The membrane was imaged with Azure Biosystems 300

Full unedited gel for Figure 4D

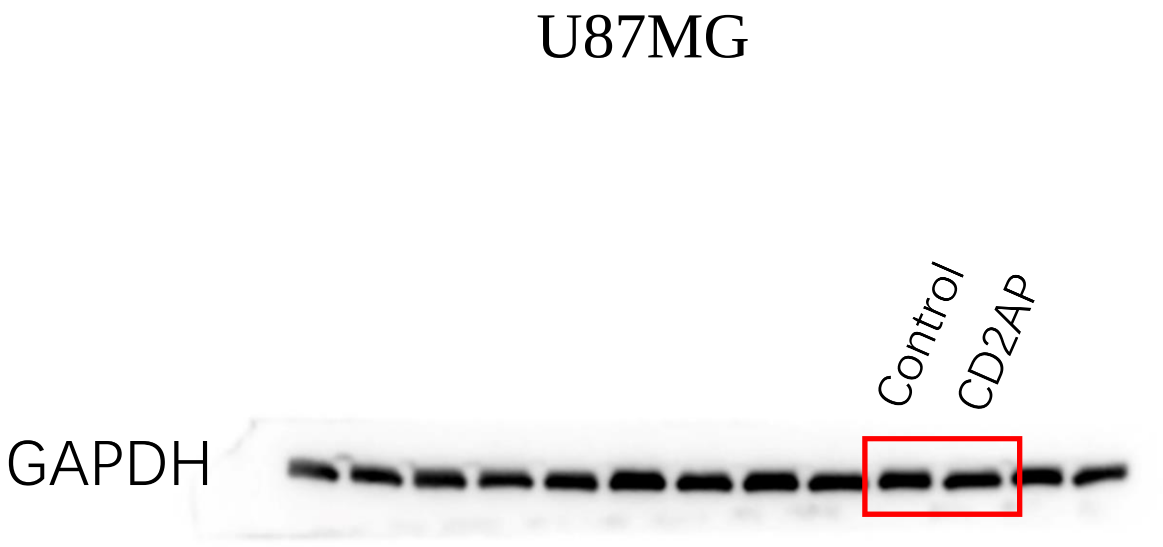

The membrane was imaged with Azure Biosystems 300

Full unedited gel for Figure 4D

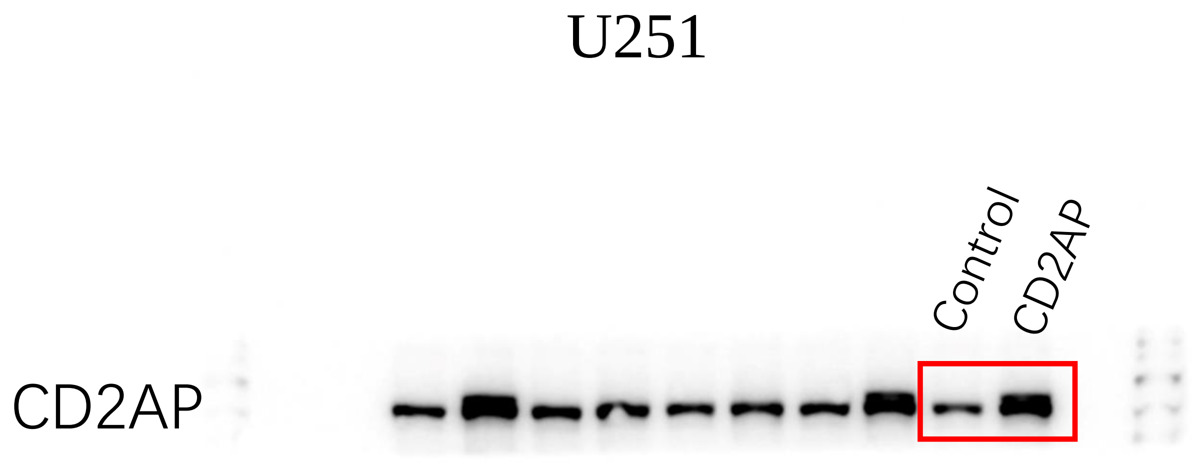

The membrane was imaged with Azure Biosystems 300

Full unedited gel for Figure 4D

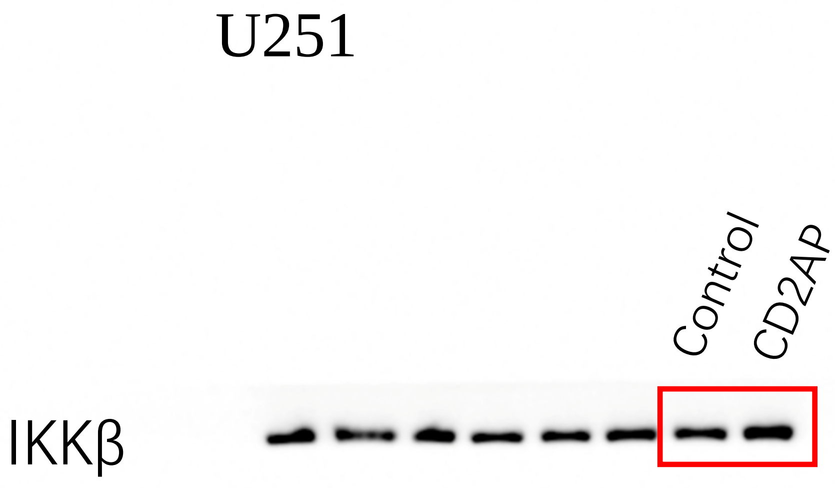

The membrane was imaged with Azure Biosystems 300

Full unedited gel for Figure 4D

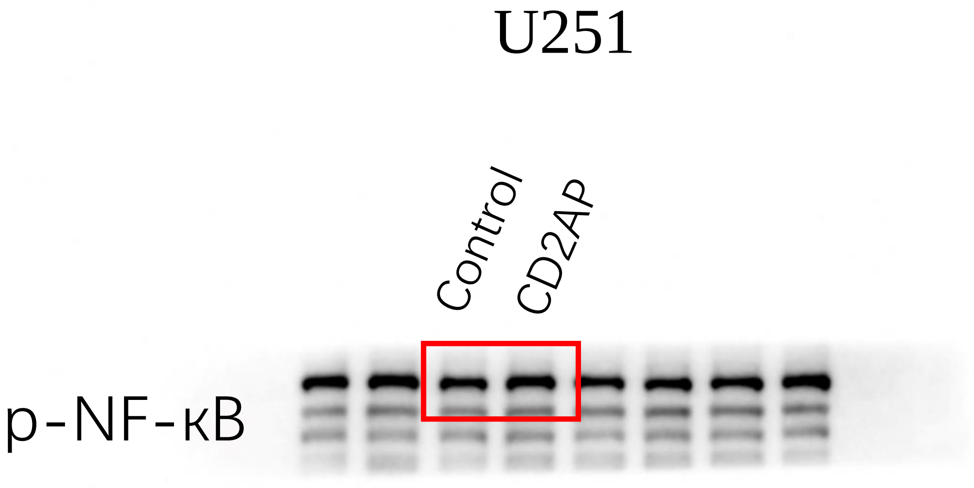

The membrane was imaged with Azure Biosystems 300

Full unedited gel for Figure 4D

U251

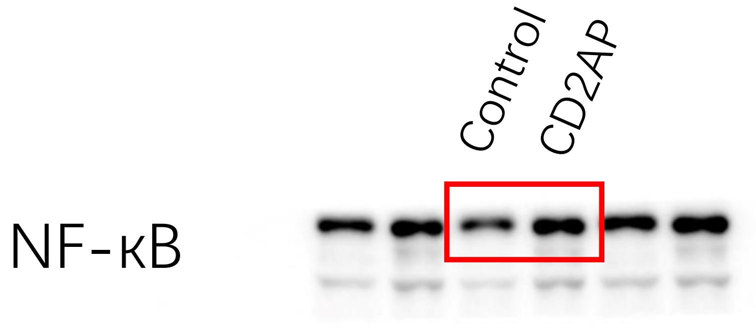

The membrane was imaged with Azure Biosystems 300

Full unedited gel for Figure 4D

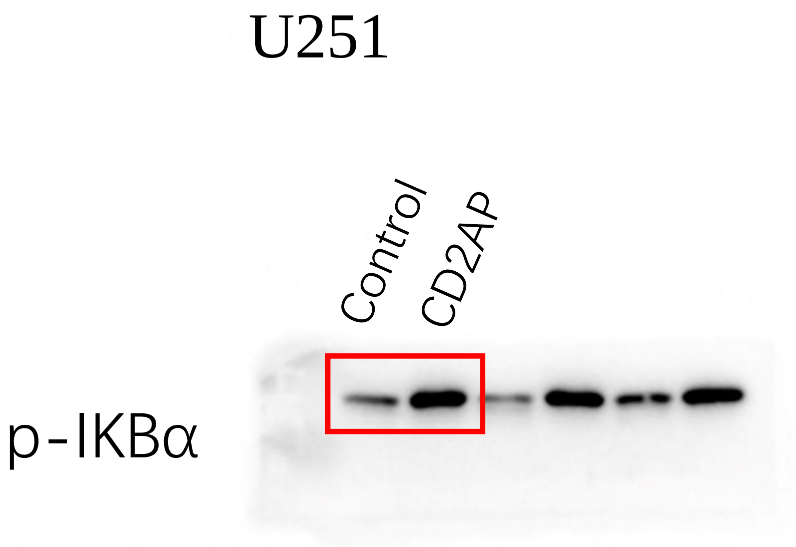

The membrane was imaged with Azure Biosystems 300

Full unedited gel for Figure 4D

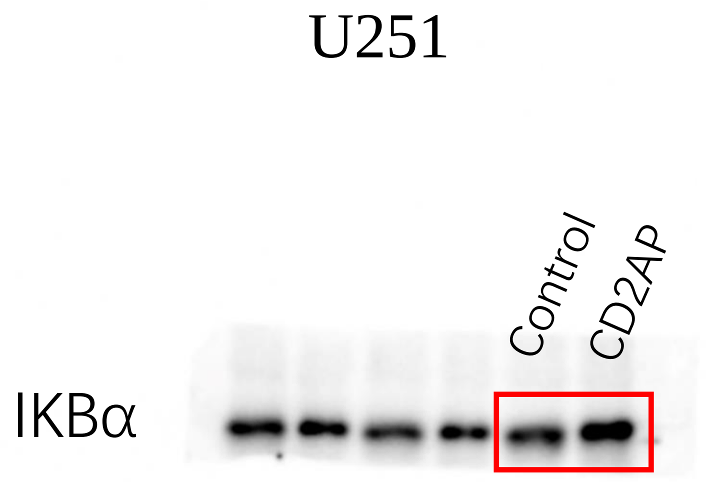

The membrane was imaged with Azure Biosystems 300

Full unedited gel for Figure 4D

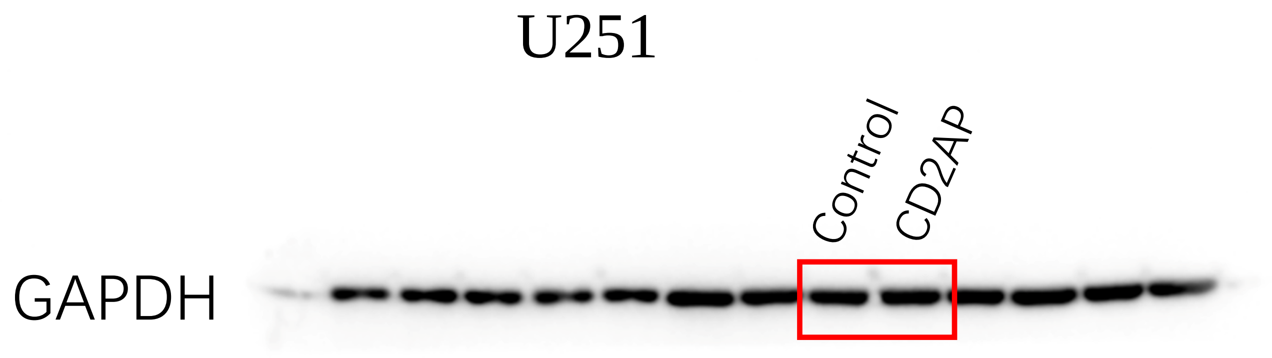

The membrane was imaged with Azure Biosystems 300

Full unedited gel for Figure 4G

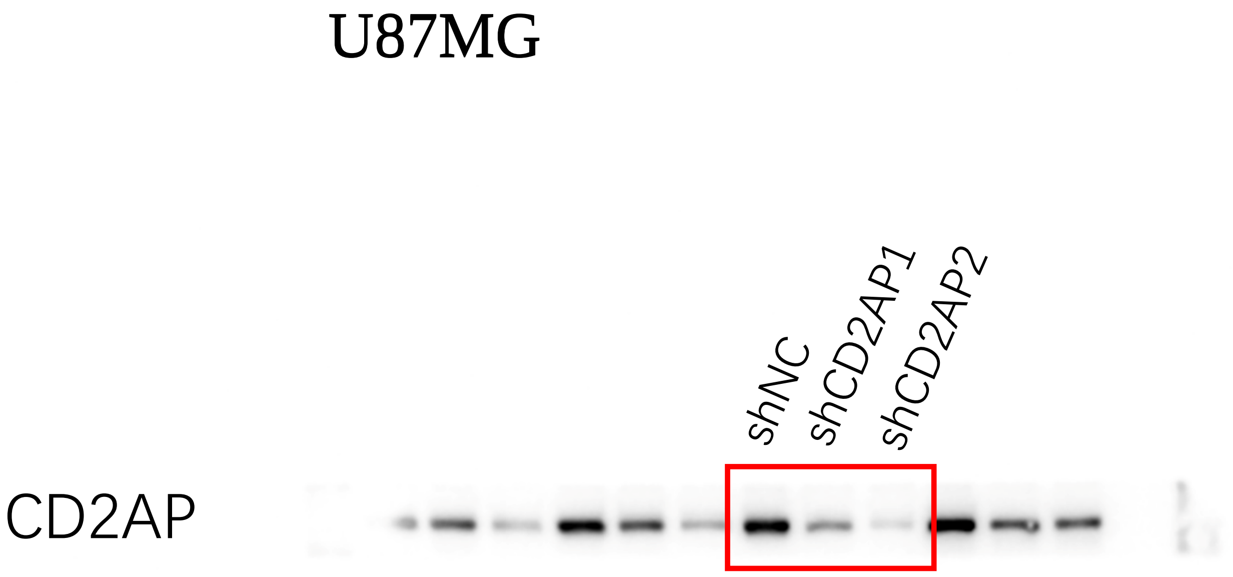

The membrane was imaged with Azure Biosystems 300

Full unedited gel for Figure 4G

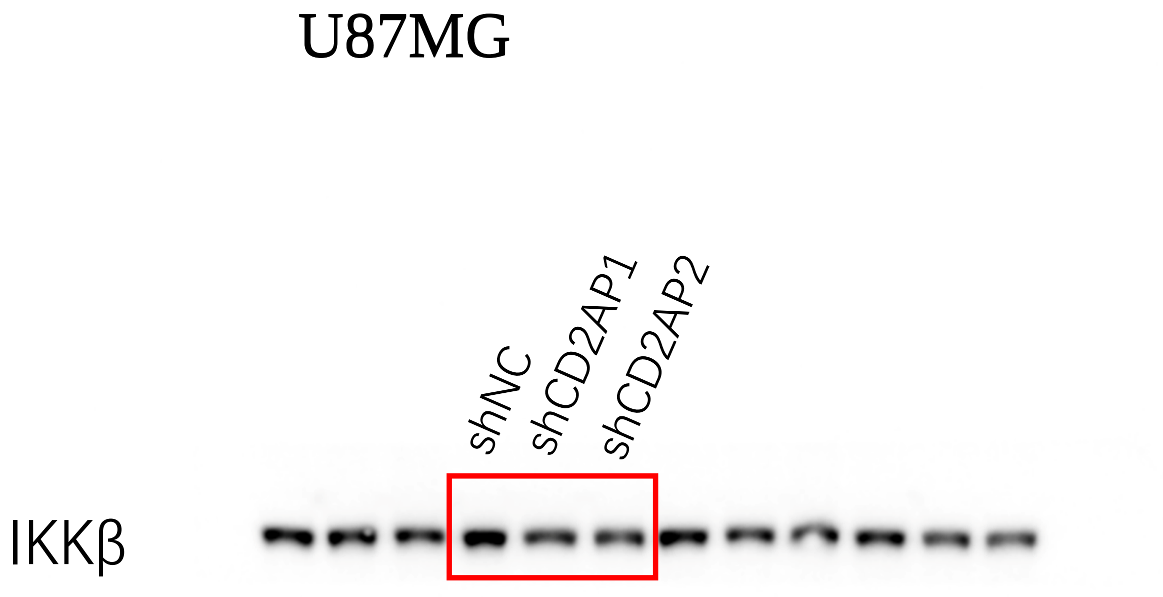

The membrane was imaged with Azure Biosystems 300

Full unedited gel for Figure 4G

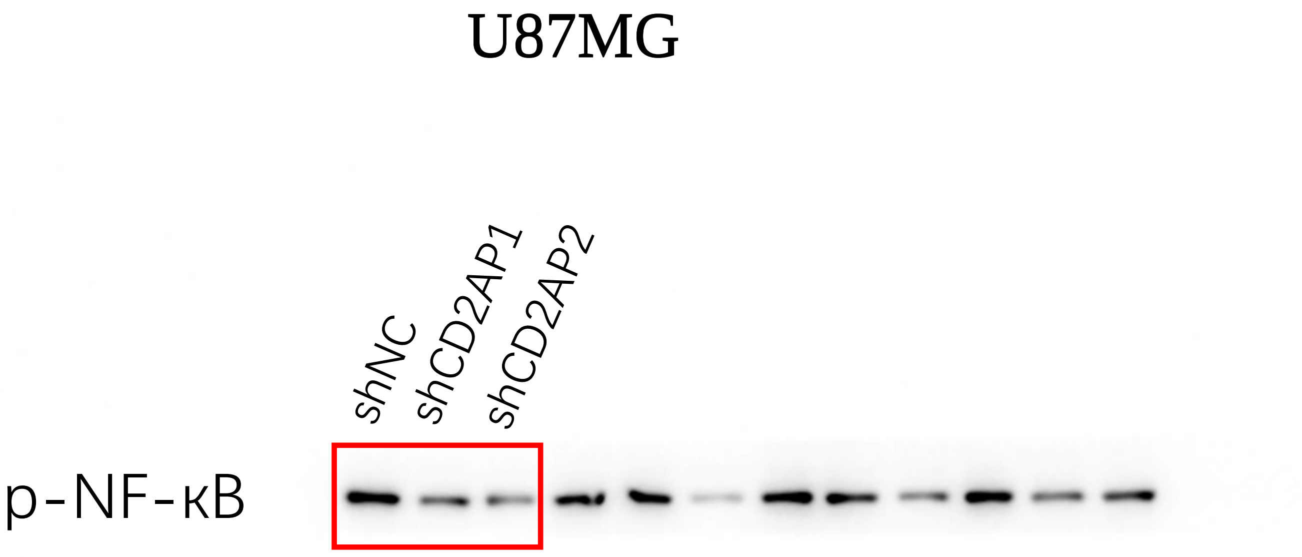

The membrane was imaged with Azure Biosystems 300

Full unedited gel for Figure 4G

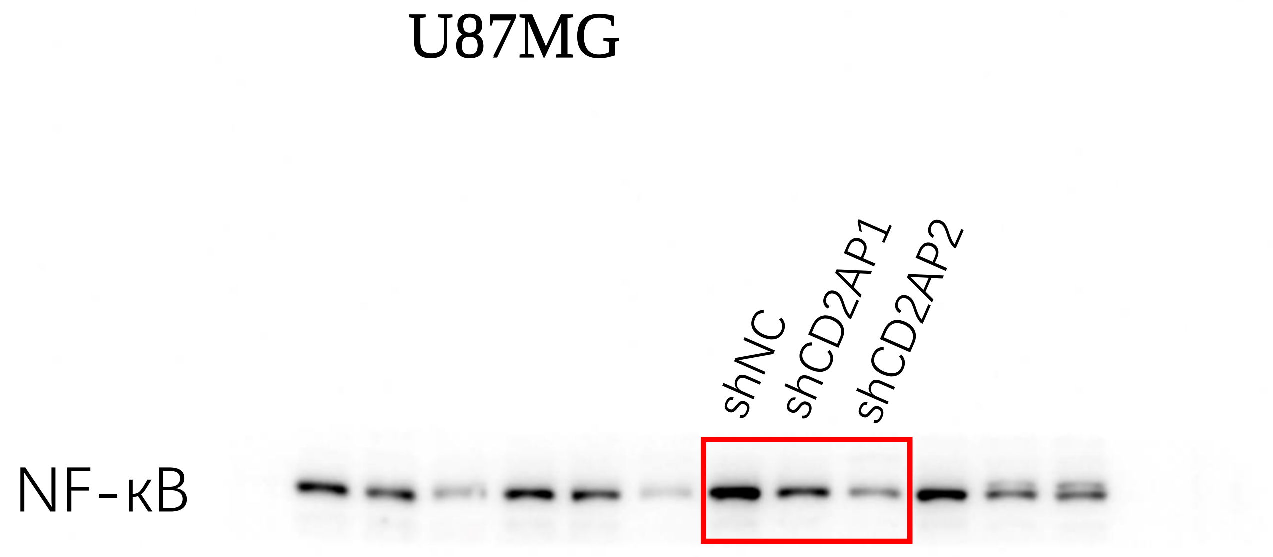

The membrane was imaged with Azure Biosystems 300

Full unedited gel for Figure 4G

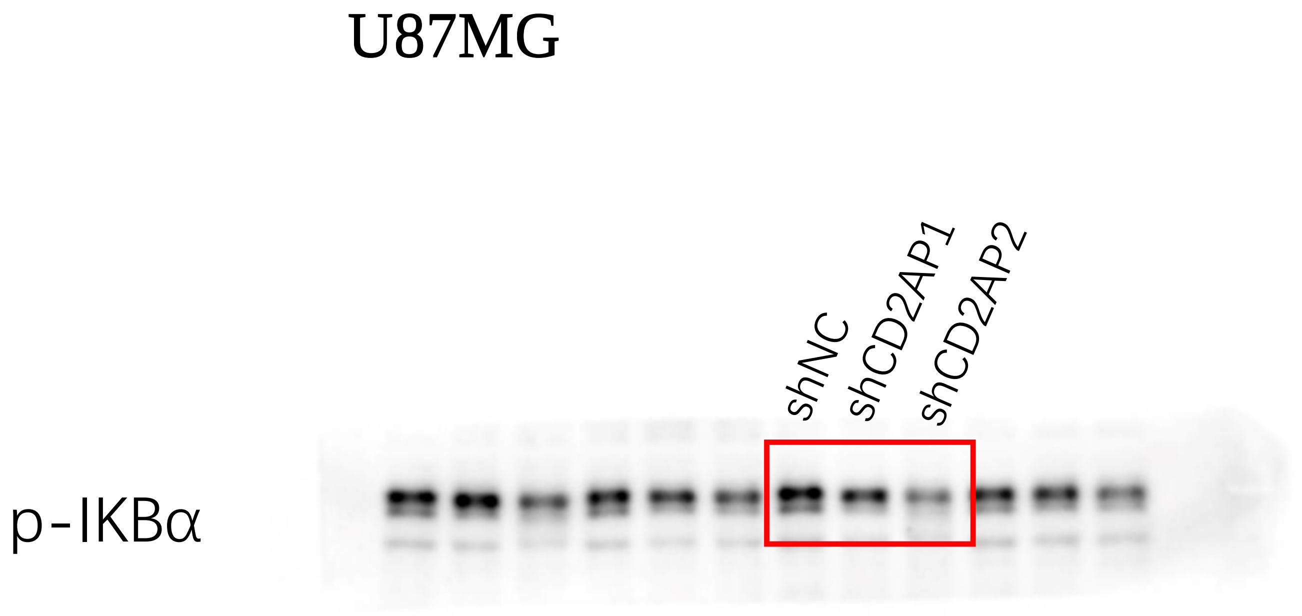

The membrane was imaged with Azure Biosystems 300

Full unedited gel for Figure 4G

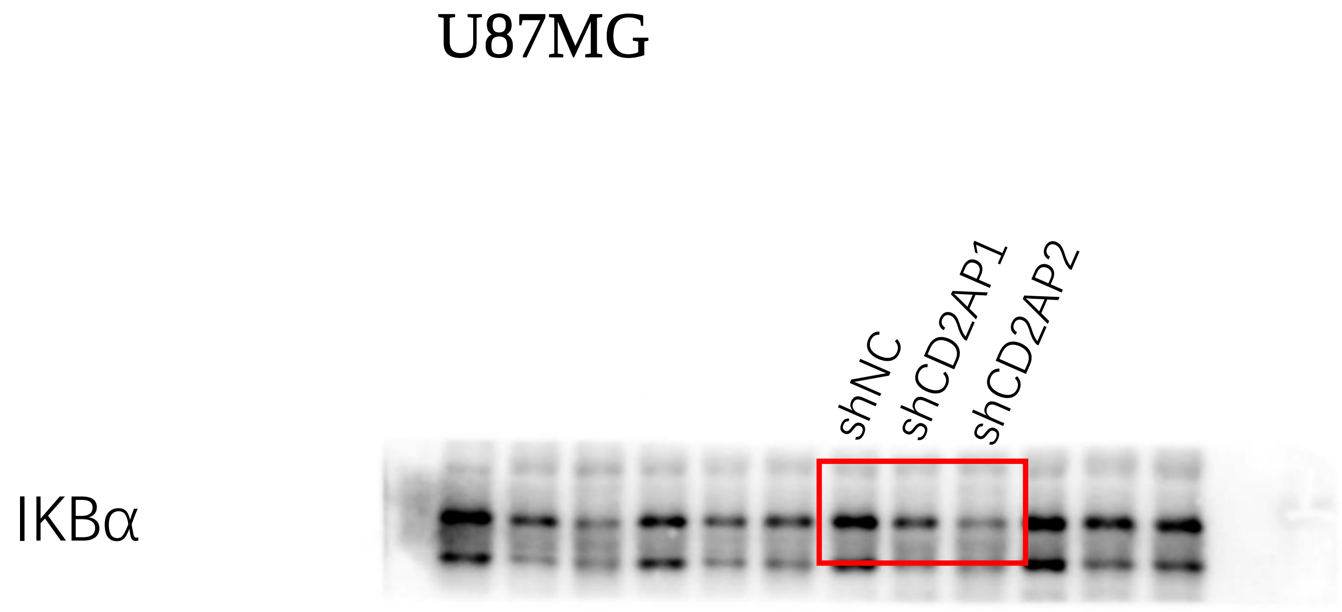

The membrane was imaged with Azure Biosystems 300

Full unedited gel for Figure 4G

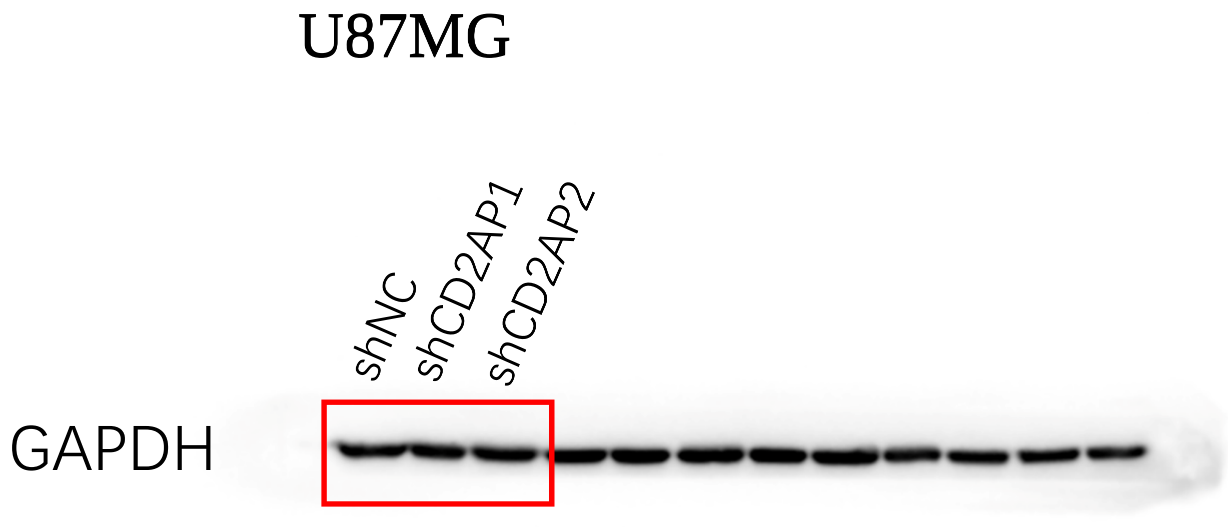

The membrane was imaged with Azure Biosystems 300

Full unedited gel for Figure 4G

U251

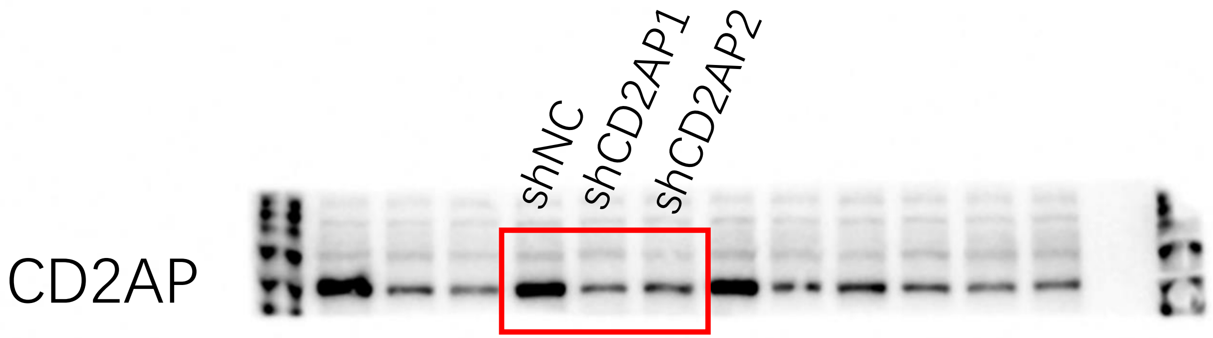

The membrane was imaged with Azure Biosystems 300

Full unedited gel for Figure 4G

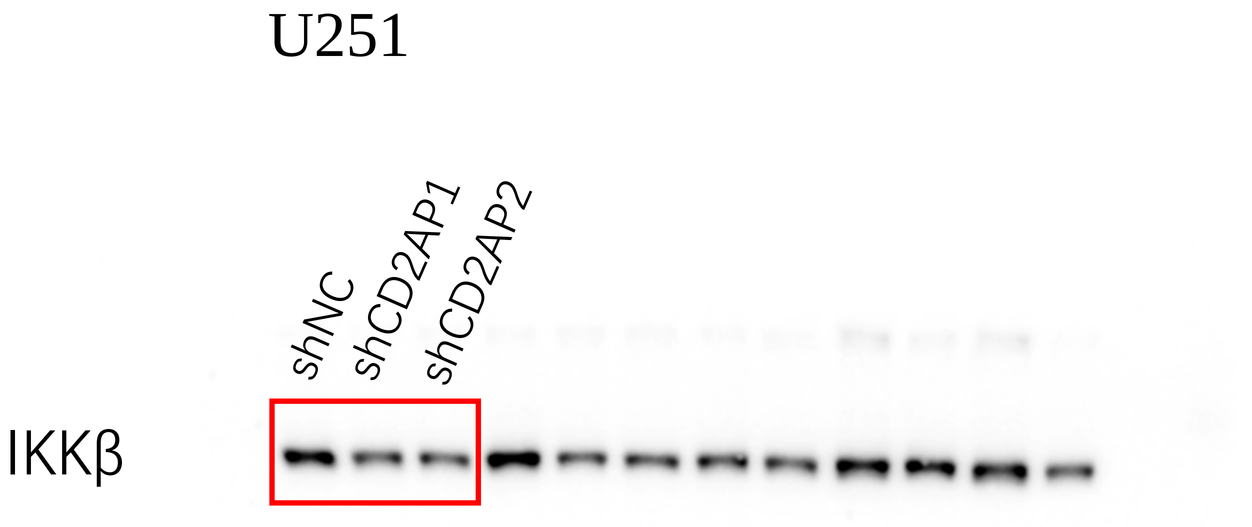

The membrane was imaged with Azure Biosystems 300

Full unedited gel for Figure 4G

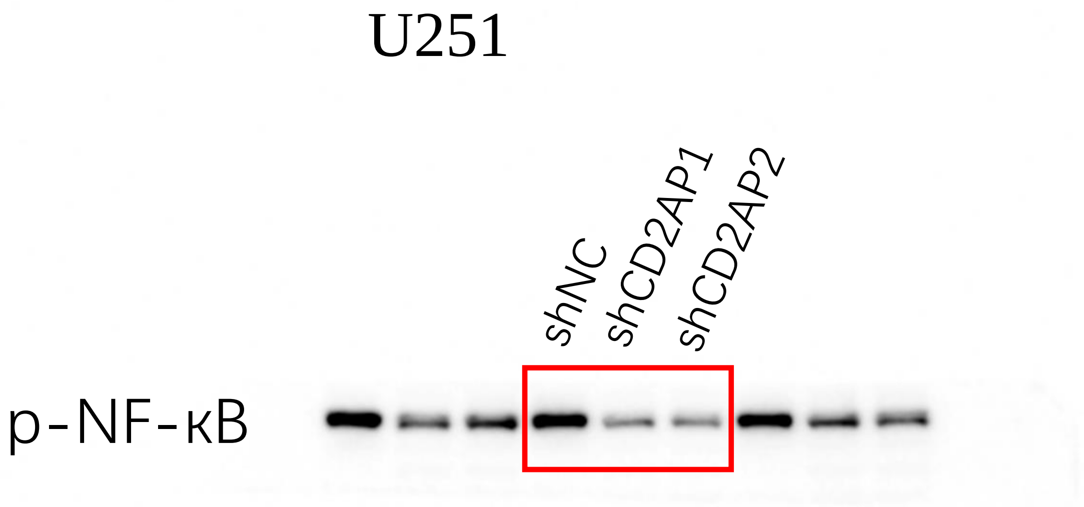

The membrane was imaged with Azure Biosystems 300

Full unedited gel for Figure 4G

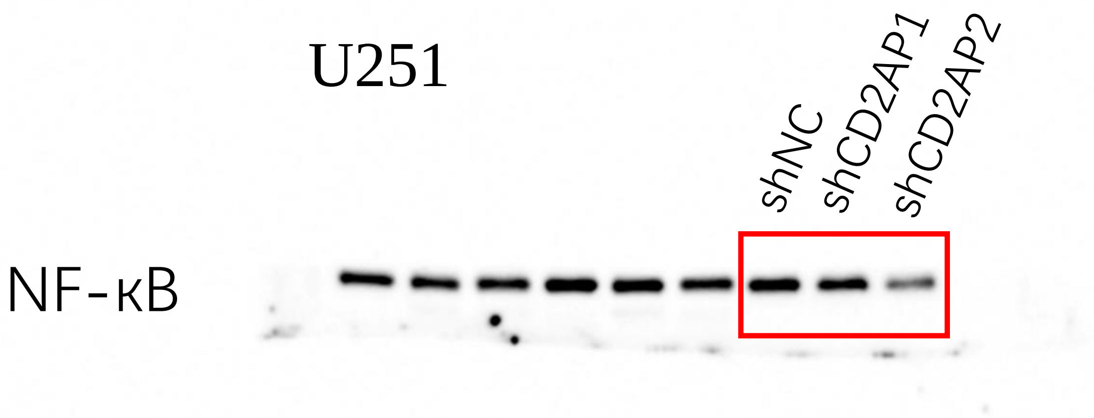

The membrane was imaged with Azure Biosystems 300

Full unedited gel for Figure 4G

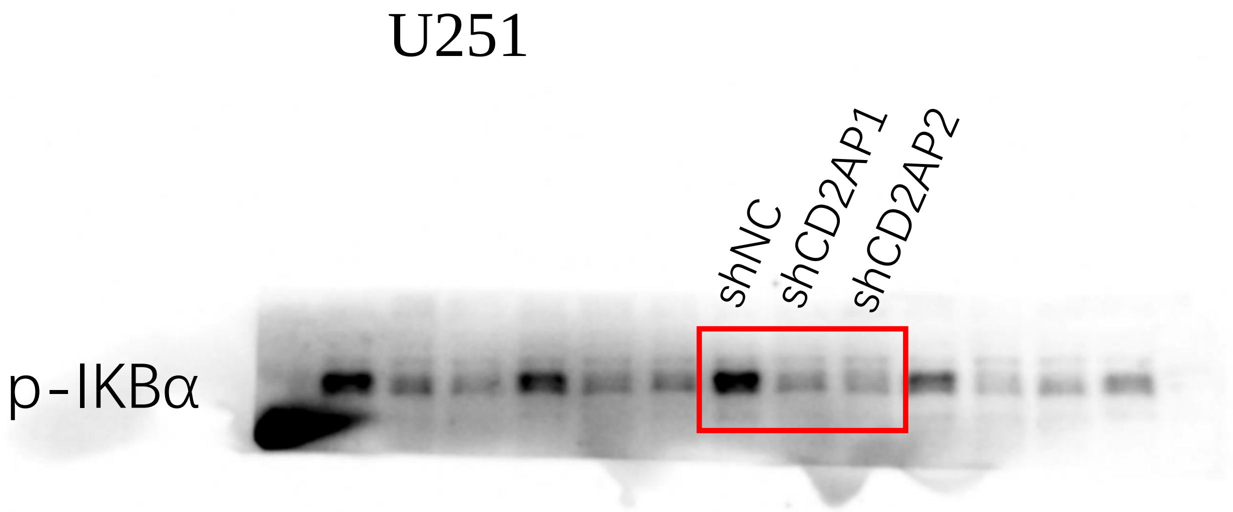

The membrane was imaged with Azure Biosystems 300

Full unedited gel for Figure 4G

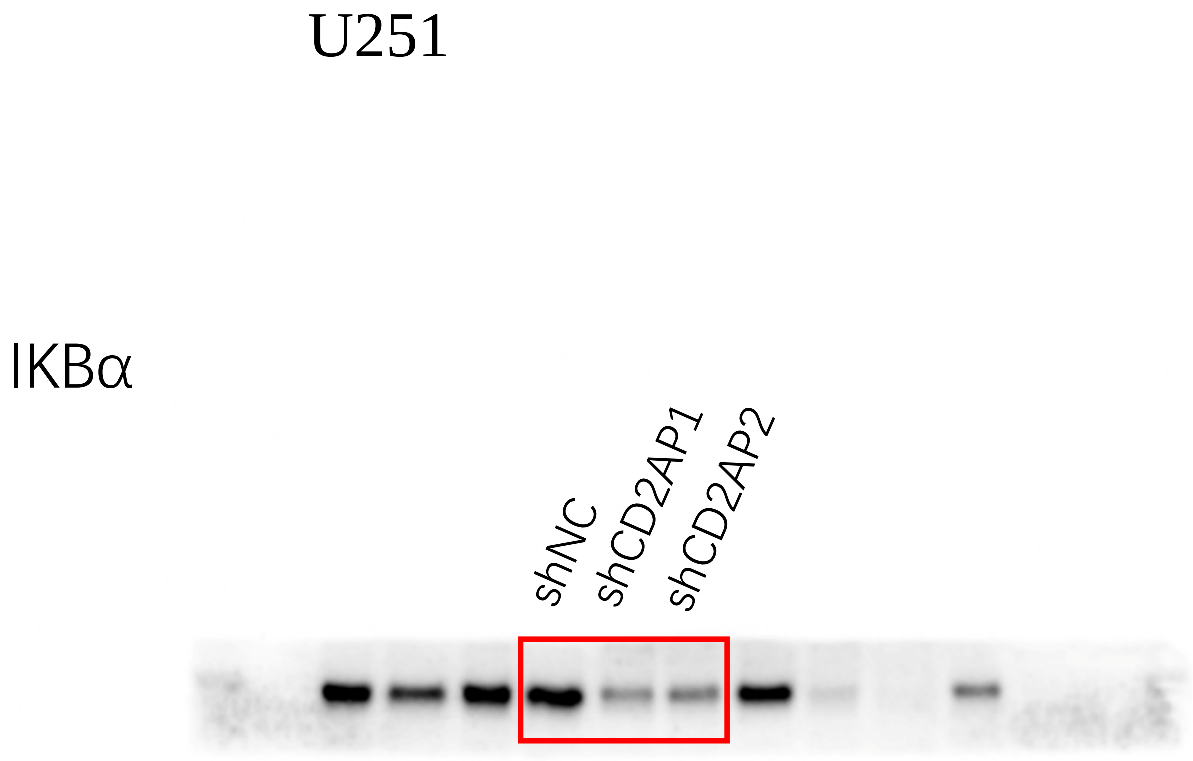

The membrane was imaged with Azure Biosystems 300

Full unedited gel for Figure 4G

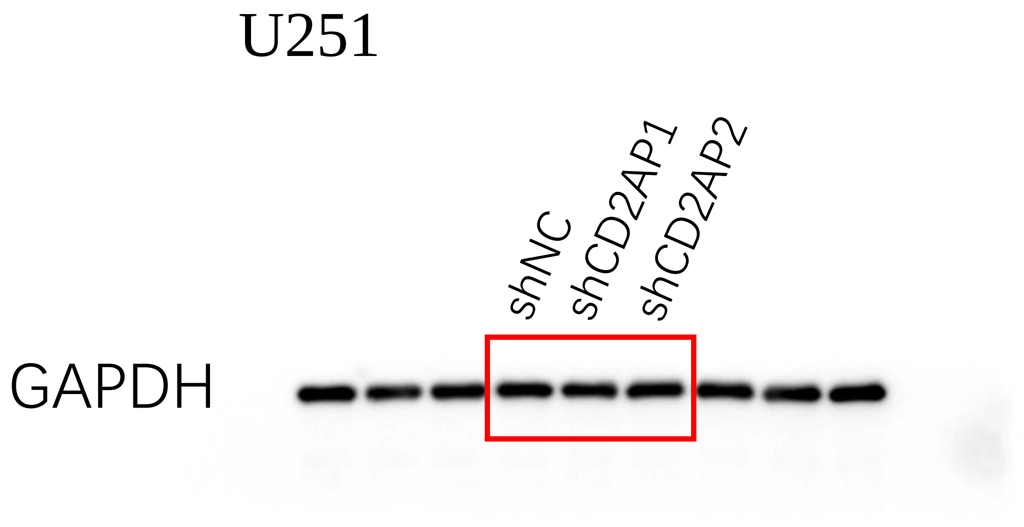

The membrane was imaged with Azure Biosystems 300

Full unedited gel for Figure 5K

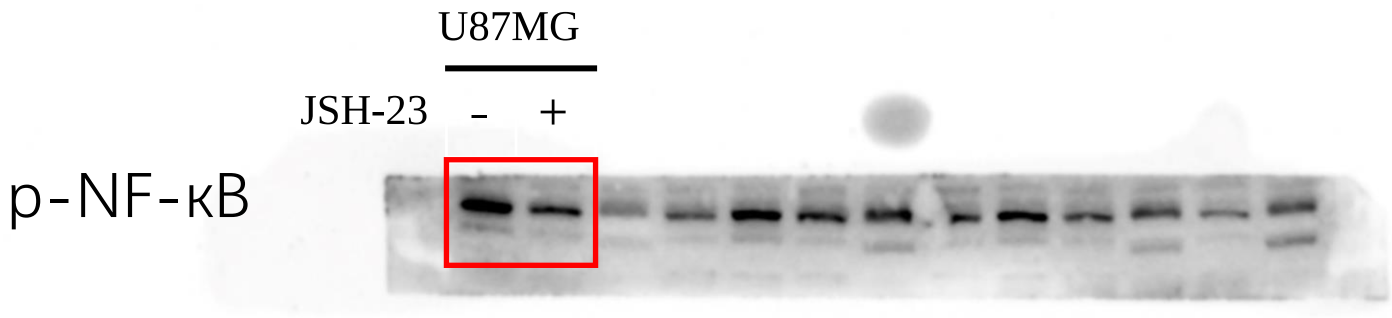

The membrane was imaged with Azure Biosystems 300

Full unedited gel for Figure 5K

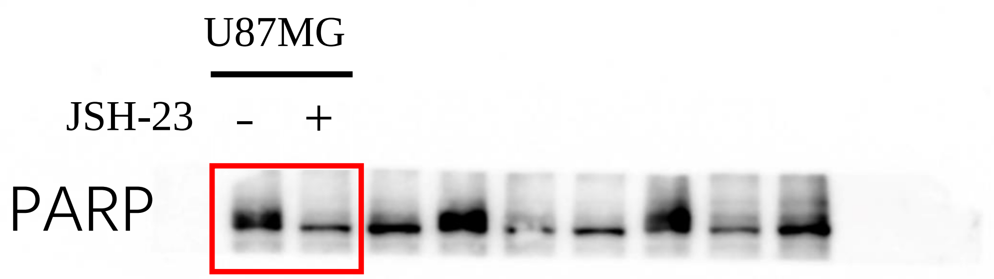

The membrane was imaged with Azure Biosystems 300

Full unedited gel for Figure 5K

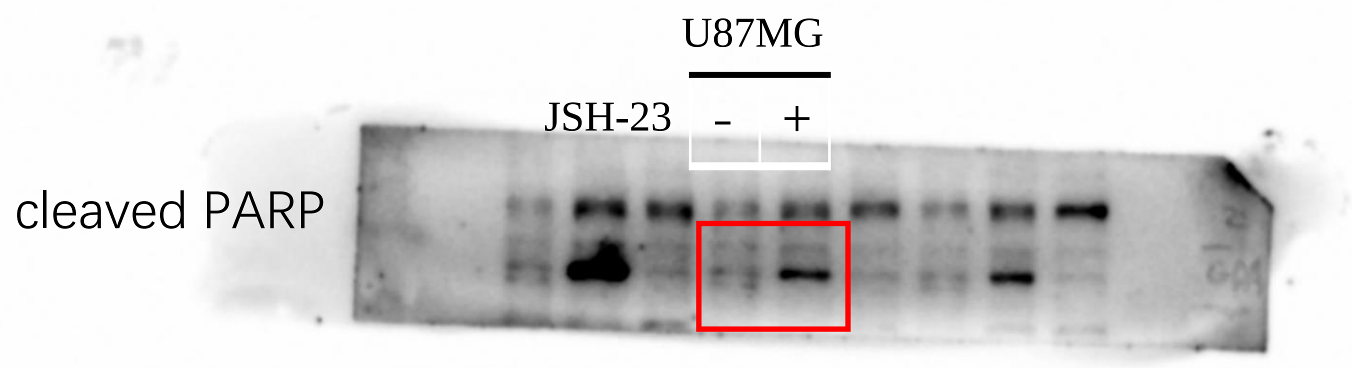

The membrane was imaged with Azure Biosystems 300

Full unedited gel for Figure 5K

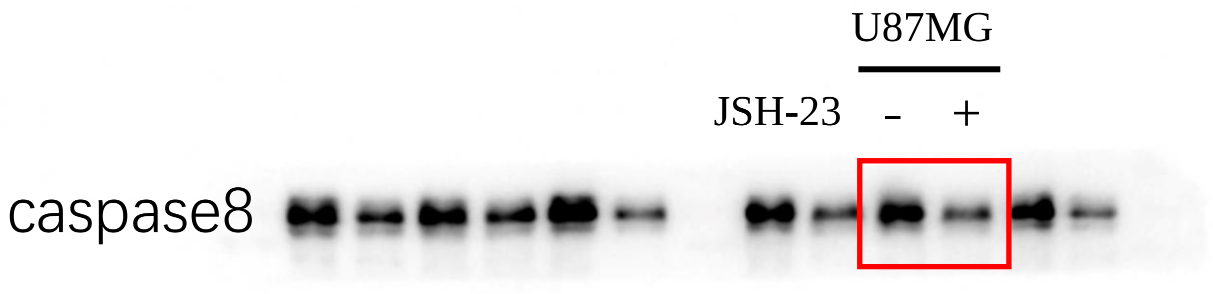

The membrane was imaged with Azure Biosystems 300

Full unedited gel for Figure 5K

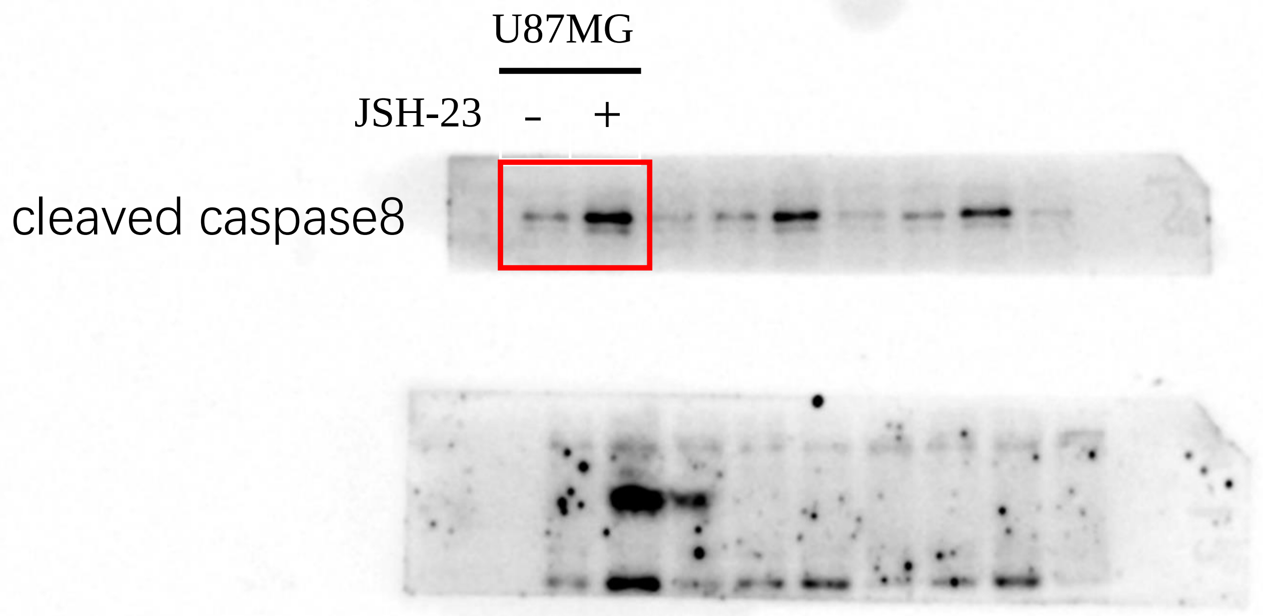

The membrane was imaged with Azure Biosystems 300

Full unedited gel for Figure 5K

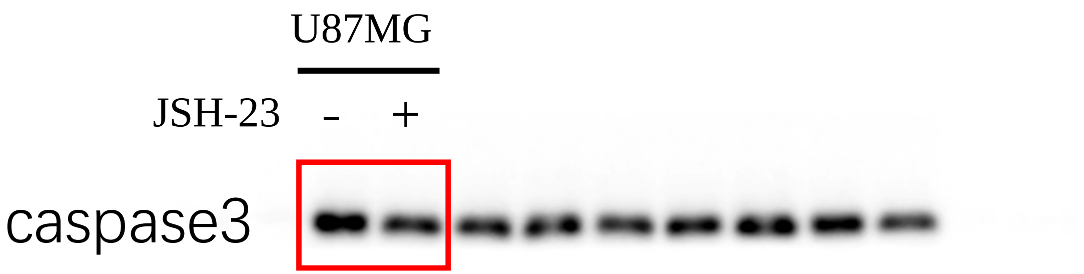

The membrane was imaged with Azure Biosystems 300

Full unedited gel for Figure 5K

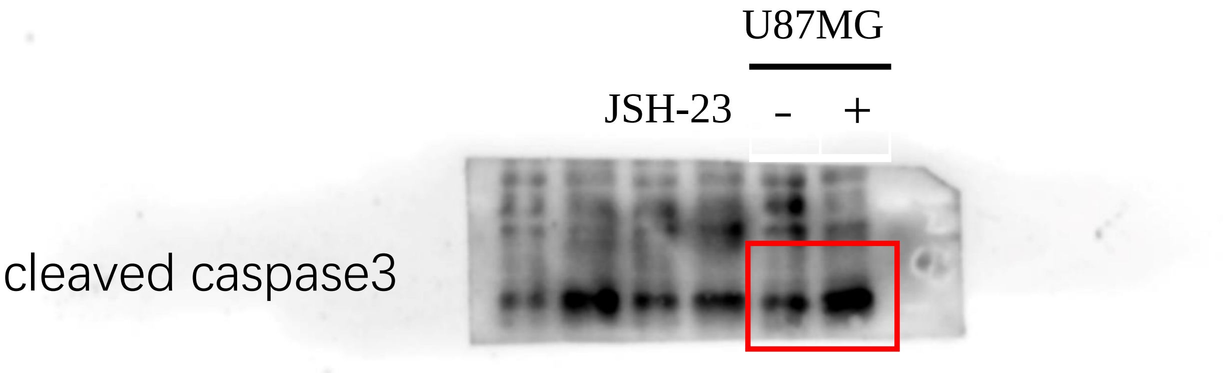

The membrane was imaged with Azure Biosystems 300

Full unedited gel for Figure 5K

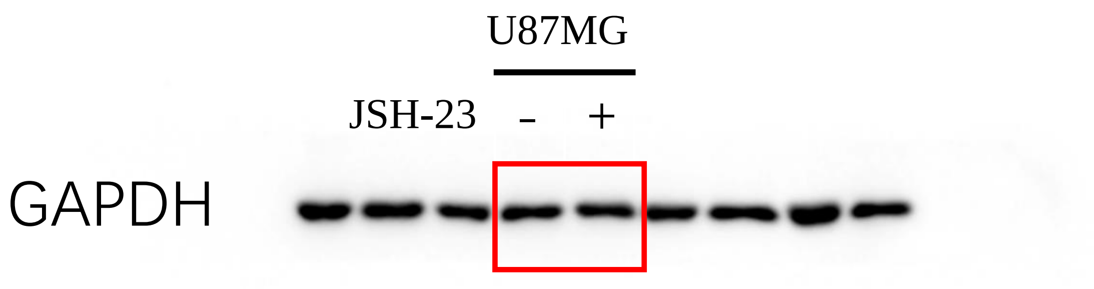

The membrane was imaged with Azure Biosystems 300

Full unedited gel for Figure 5K

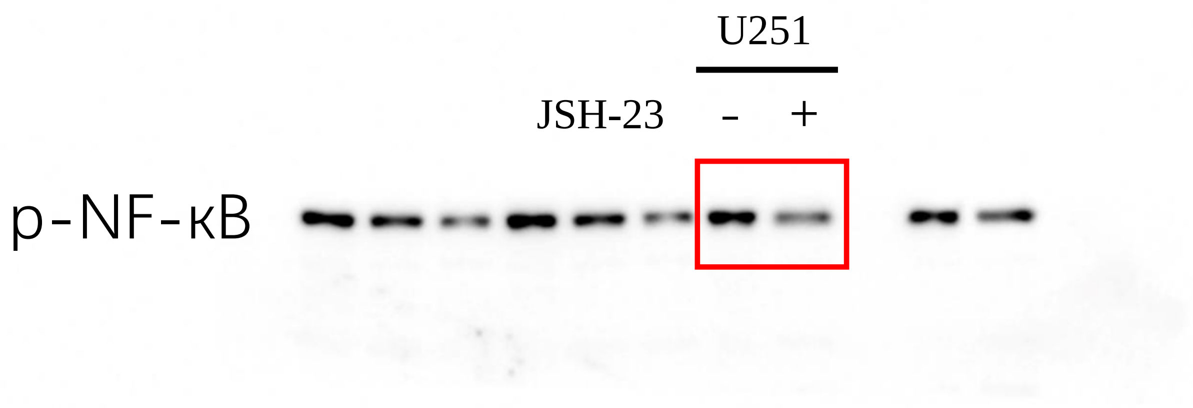

The membrane was imaged with Azure Biosystems 300

Full unedited gel for Figure 5K

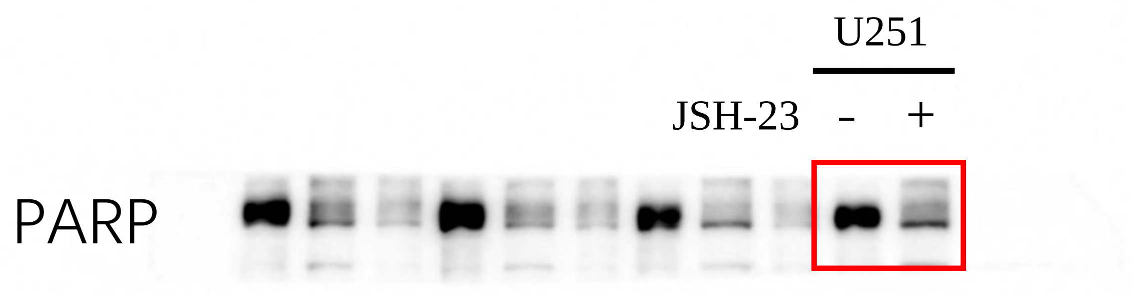

The membrane was imaged with Azure Biosystems 300

Full unedited gel for Figure 5K

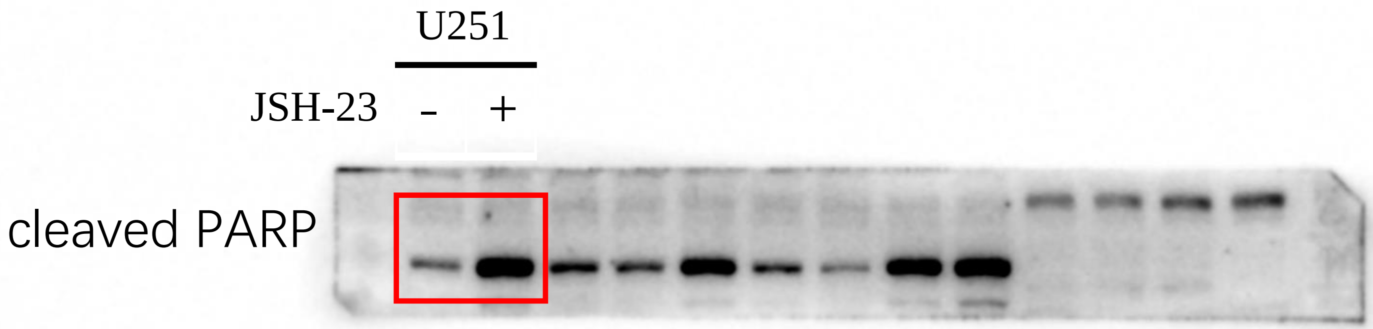

The membrane was imaged with Azure Biosystems 300

Full unedited gel for Figure 5K

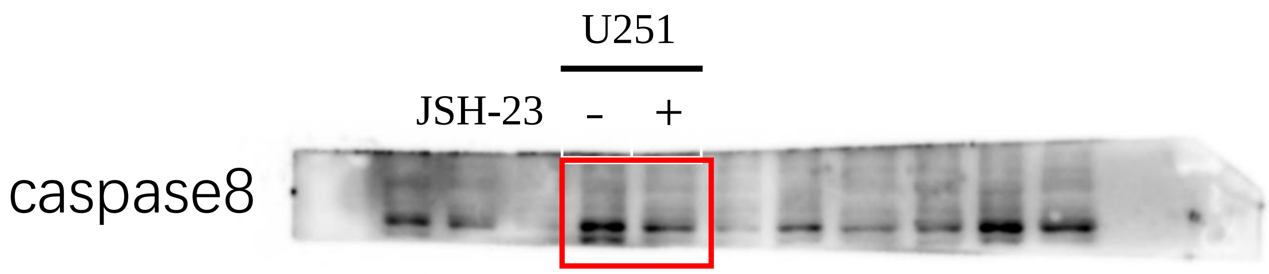

The membrane was imaged with Azure Biosystems 300

Full unedited gel for Figure 5K

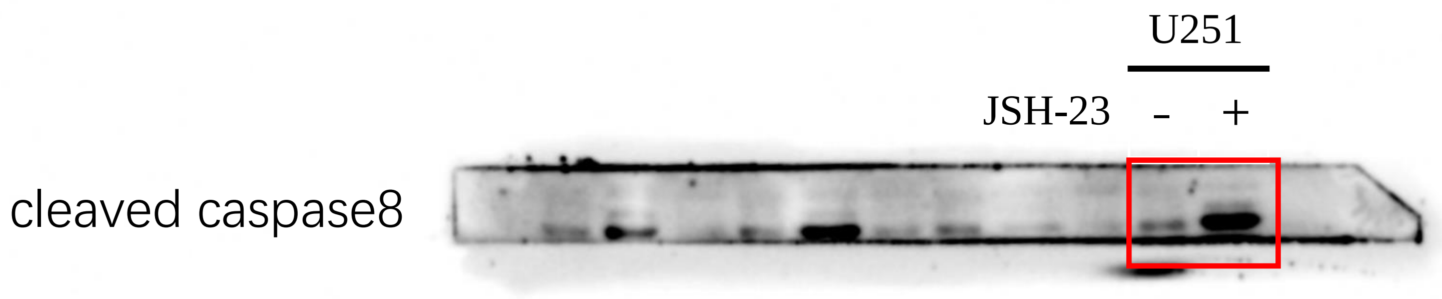

The membrane was imaged with Azure Biosystems 300

Full unedited gel for Figure 5K

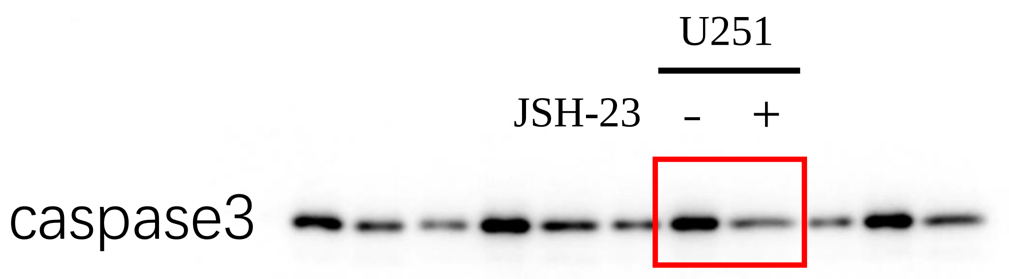

The membrane was imaged with Azure Biosystems 300

Full unedited gel for Figure 5K

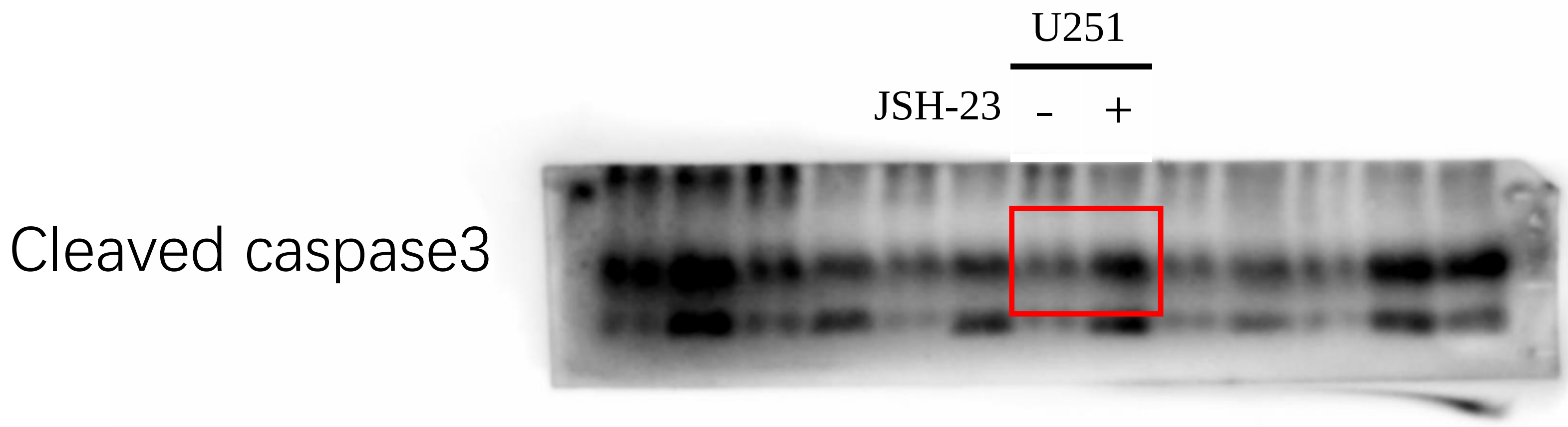

The membrane was imaged with Azure Biosystems 300

Full unedited gel for Figure 5K

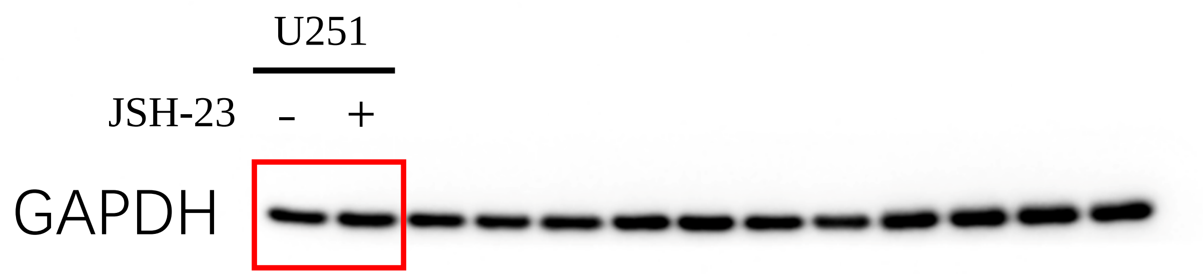

The membrane was imaged with Azure Biosystems 300

Full unedited gel for Figure 6B

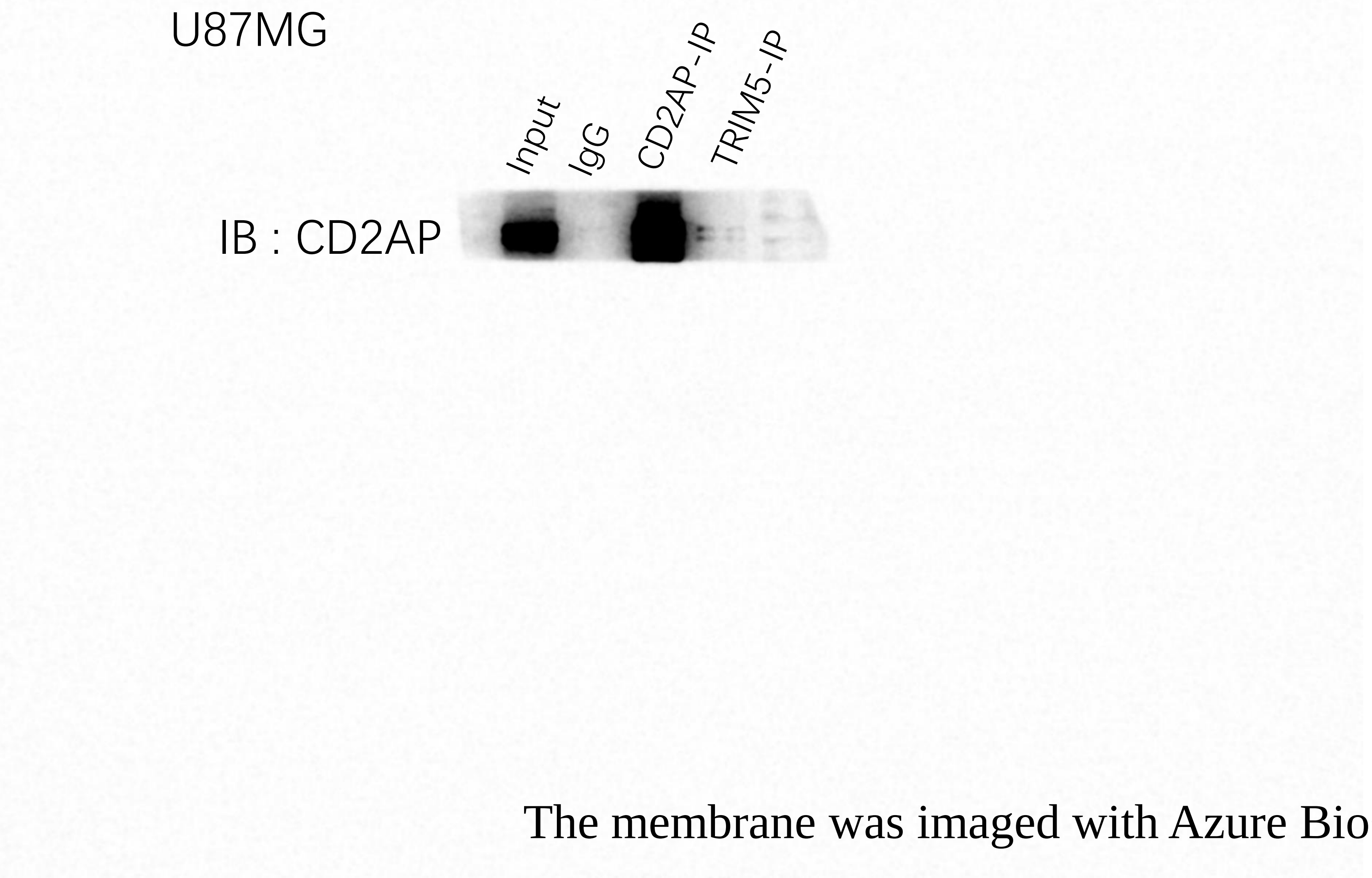

The membrane was imaged with Azure Biosystems 300

Full unedited gel for Figure 6B

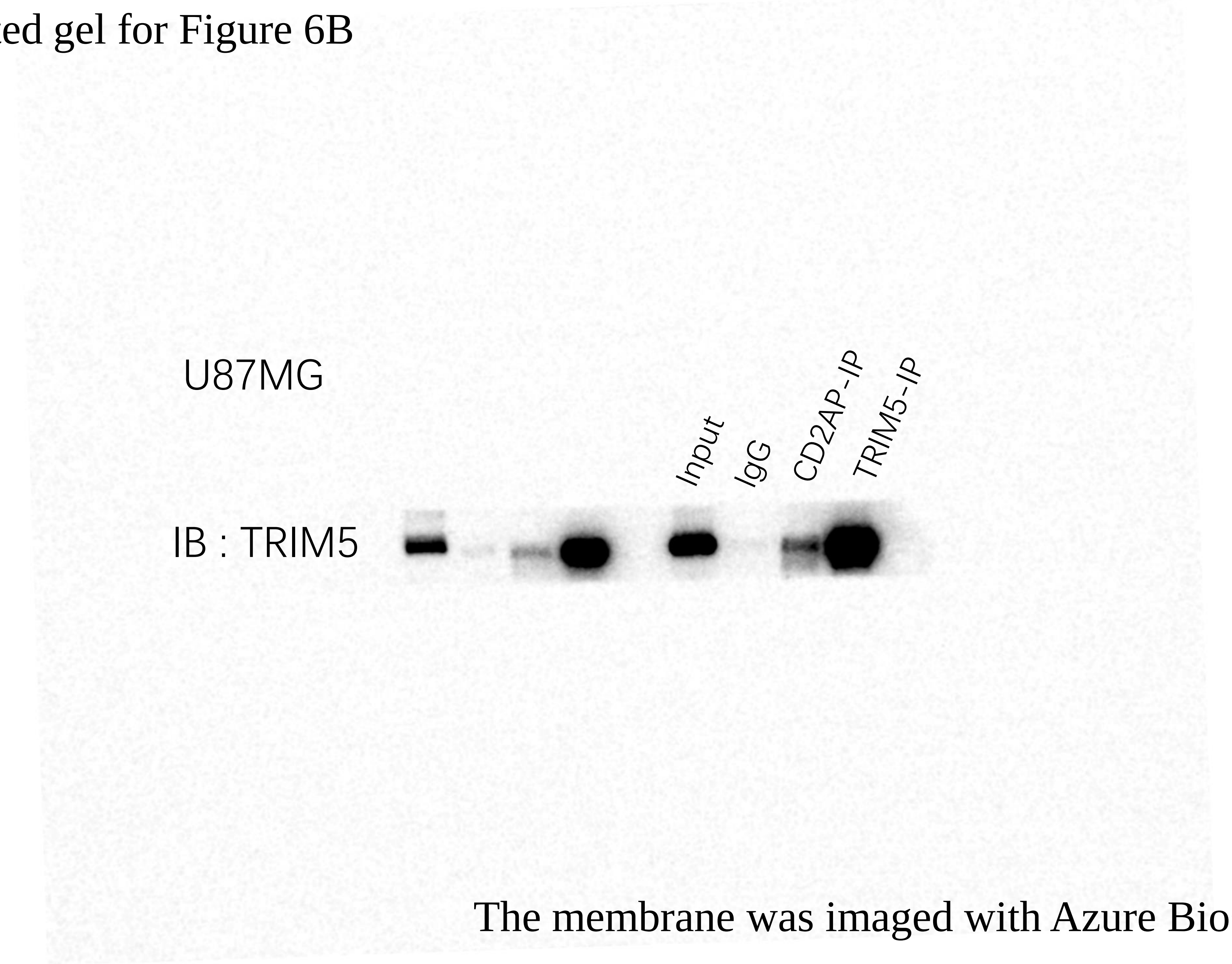

The membrane was imaged with Azure Biosystems 300

Full unedited gel for Figure 6C

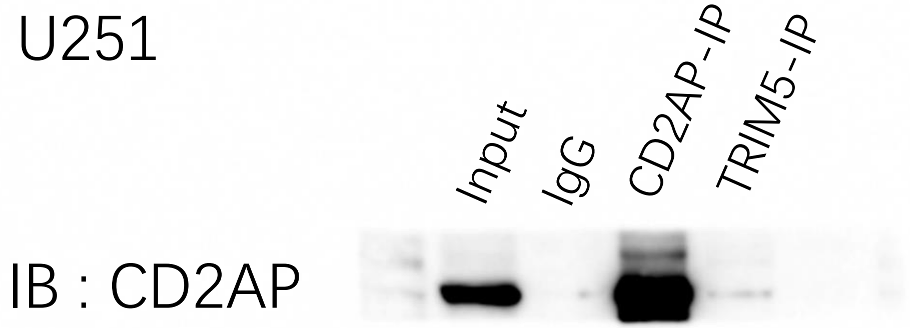

The membrane was imaged with Azure Biosystems 300

Full unedited gel for Figure 6C

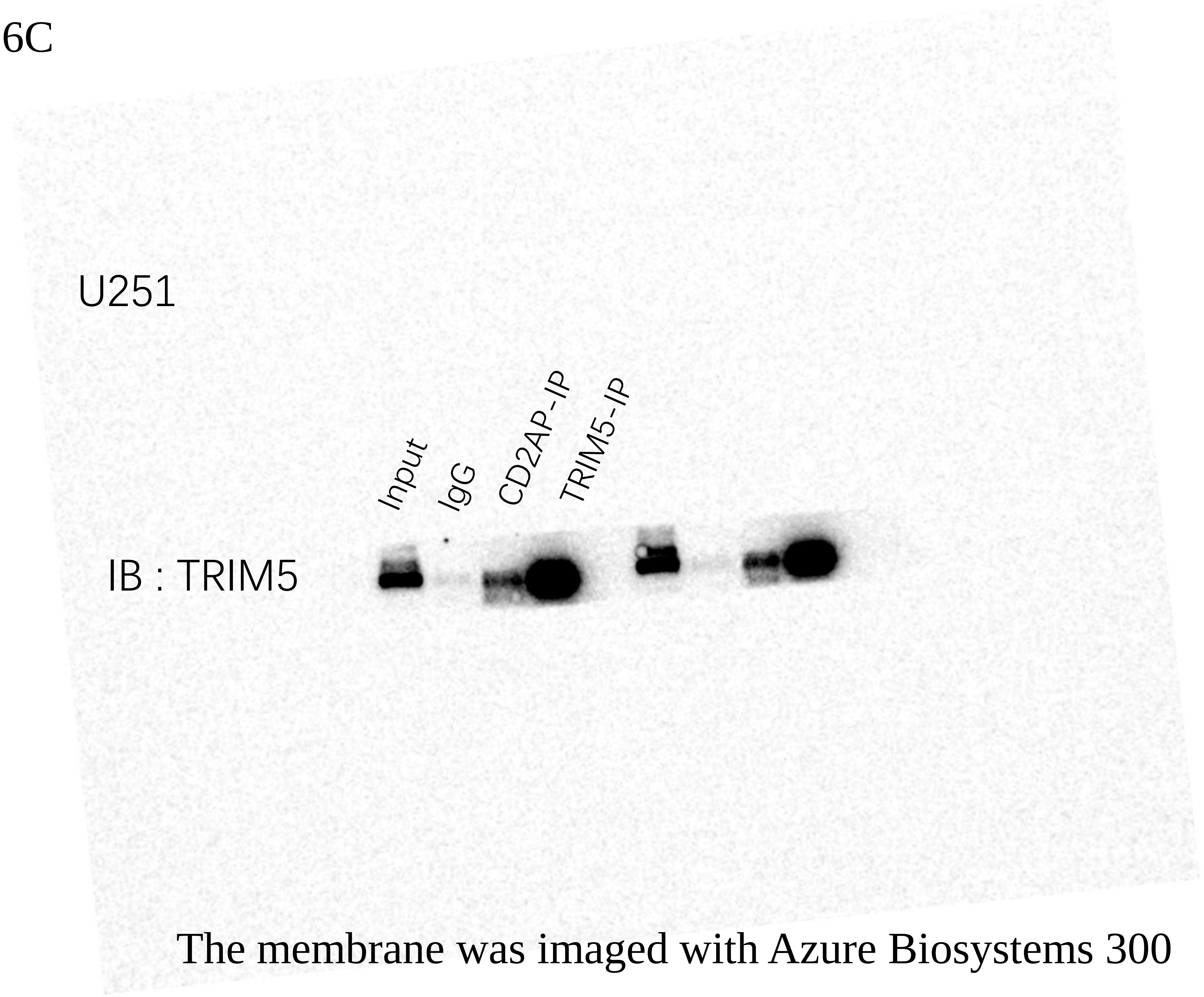

The membrane was imaged with Azure Biosystems 300

Full unedited gel for Figure 6D

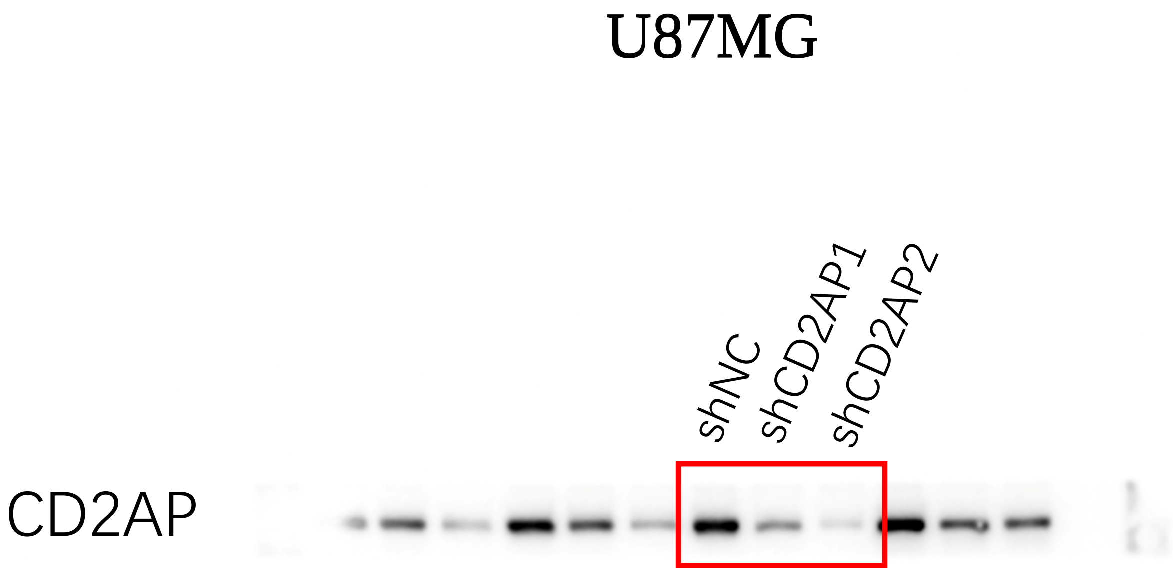

The membrane was imaged with Azure Biosystems 300

Full unedited gel for Figure 6D

U87MG

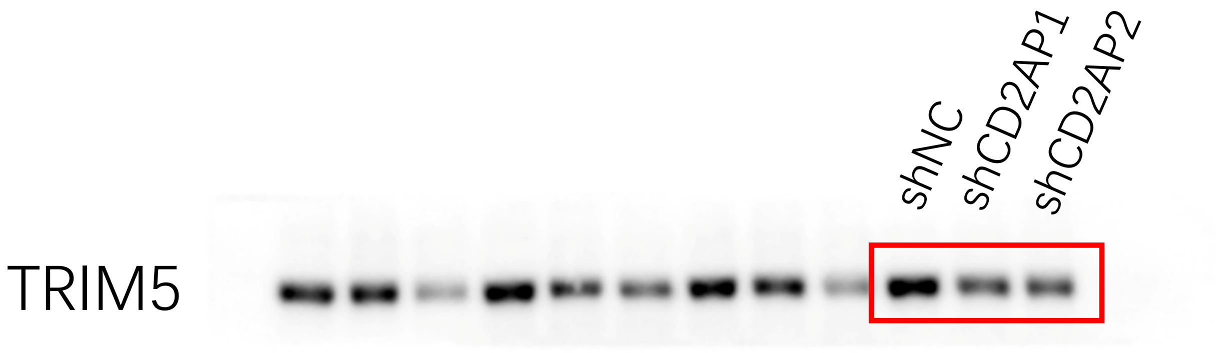

The membrane was imaged with Azure Biosystems 300

Full unedited gel for Figure 6D

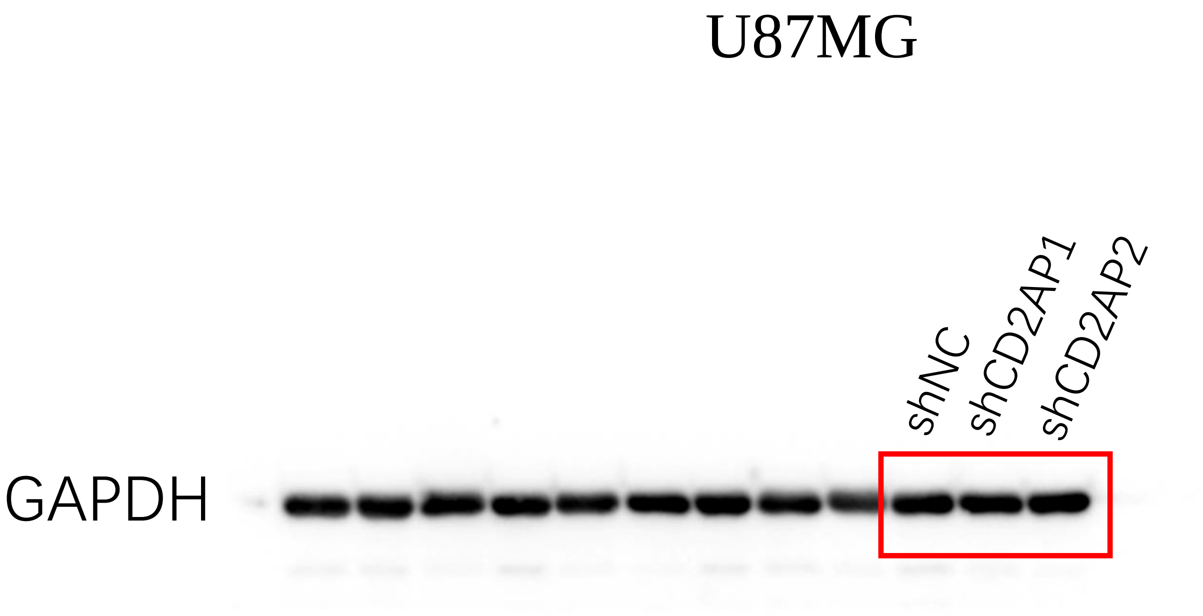

The membrane was imaged with Azure Biosystems 300

Full unedited gel for Figure 6D

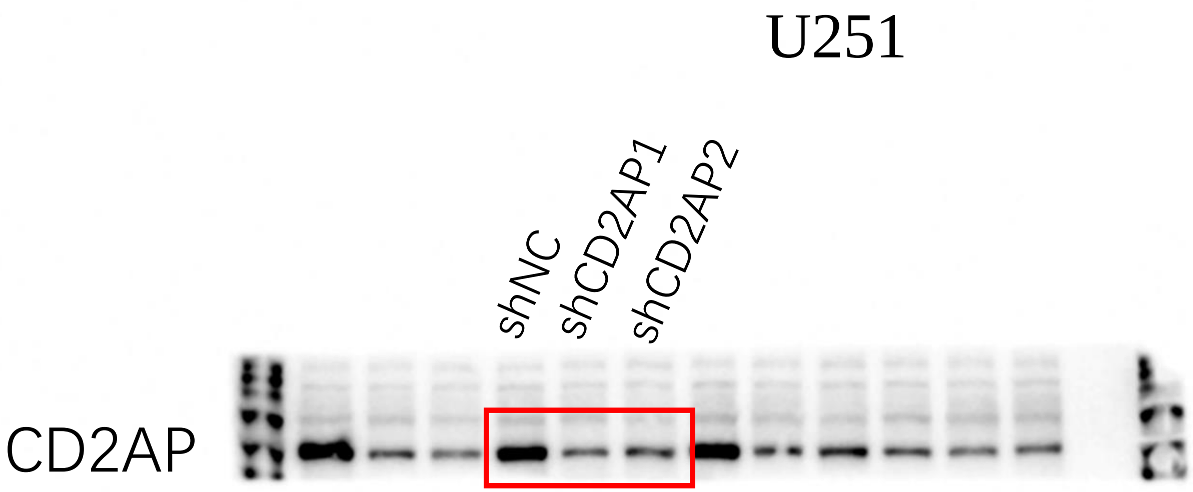

The membrane was imaged with Azure Biosystems 300

Full unedited gel for Figure 6D

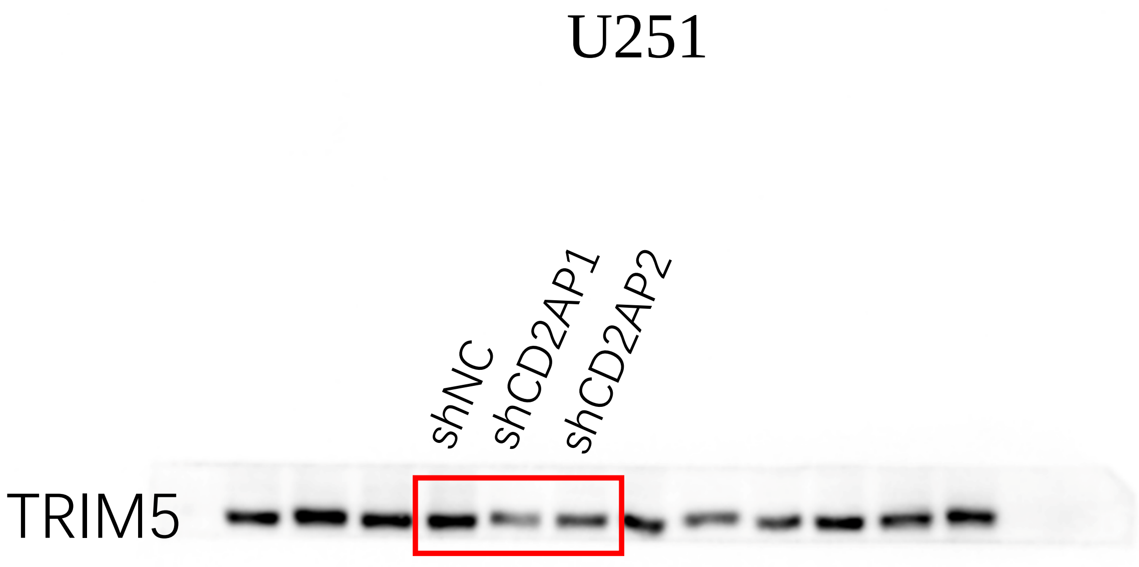

The membrane was imaged with Azure Biosystems 300

Full unedited gel for Figure 6D

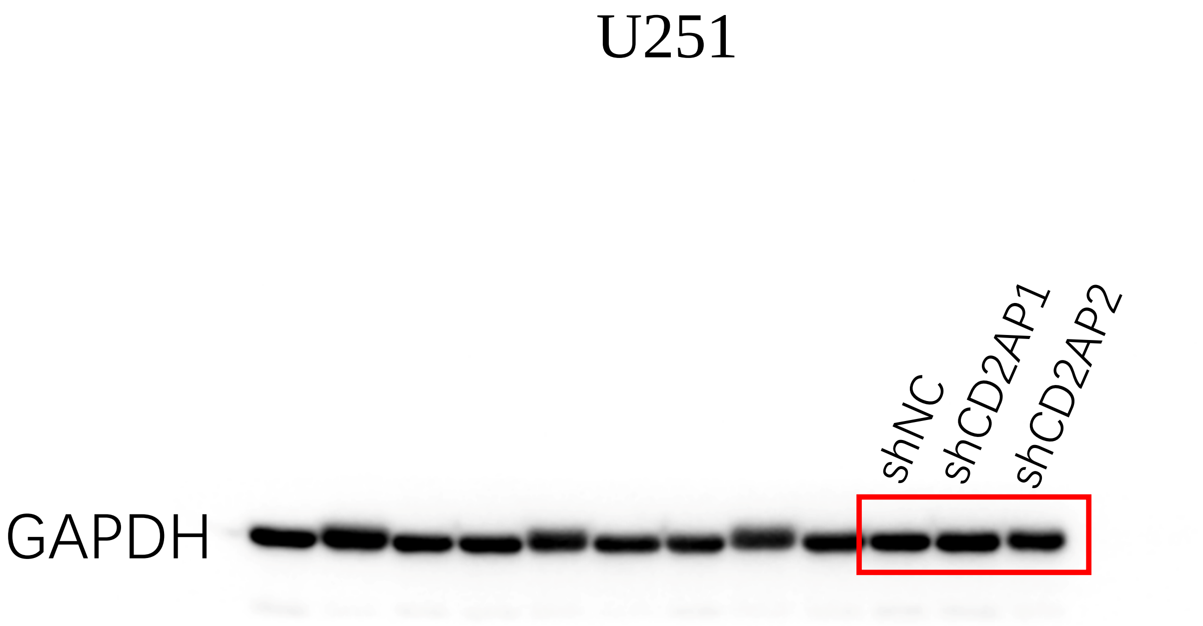

The membrane was imaged with Azure Biosystems 300

Full unedited gel for Figure 6F

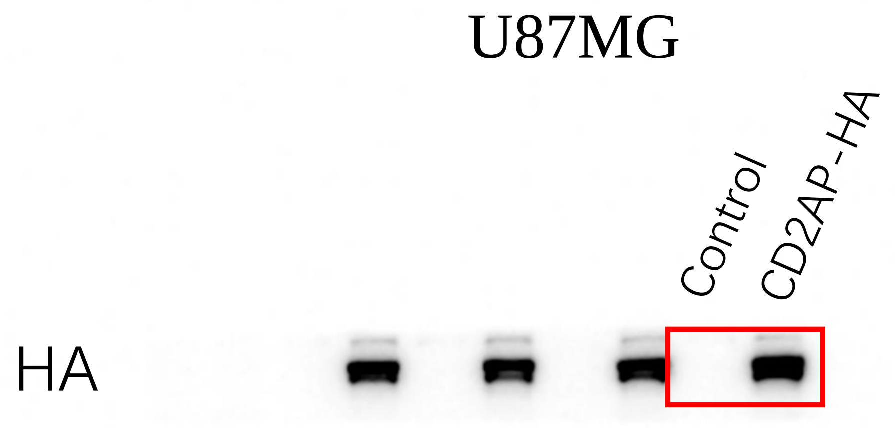

The membrane was imaged with Azure Biosystems 300

Full unedited gel for Figure 6F

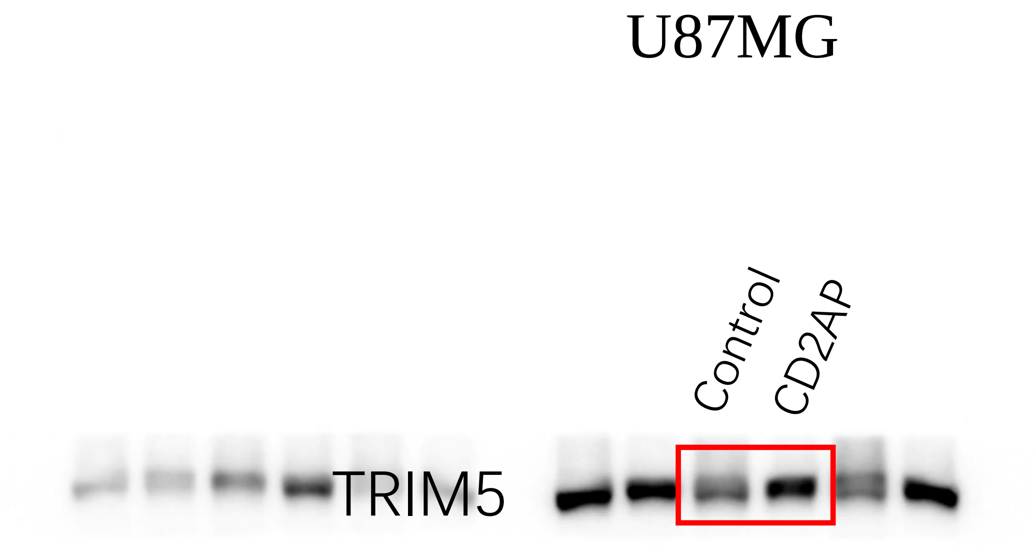

The membrane was imaged with Azure Biosystems 300

Full unedited gel for Figure 6F

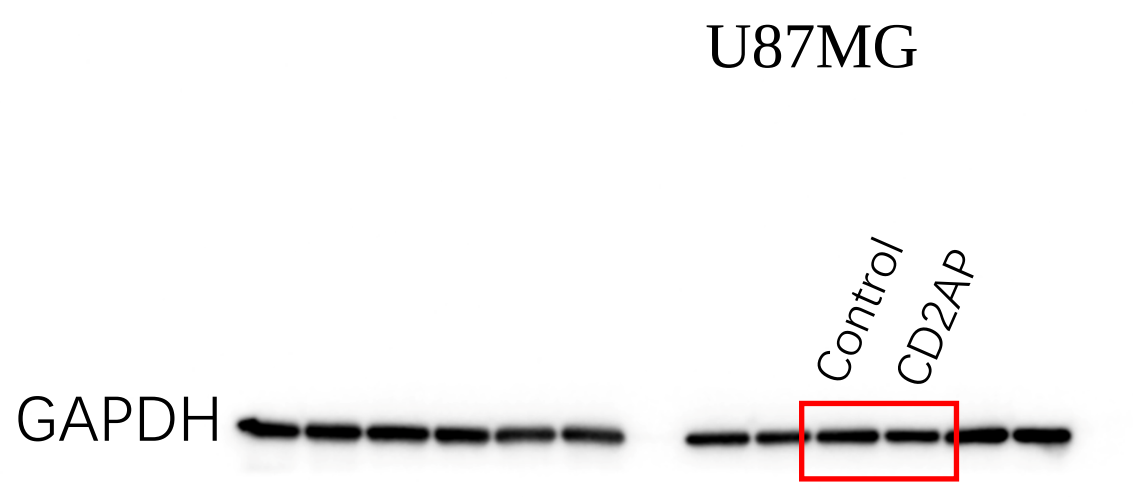

The membrane was imaged with Azure Biosystems 300

Full unedited gel for Figure 6F

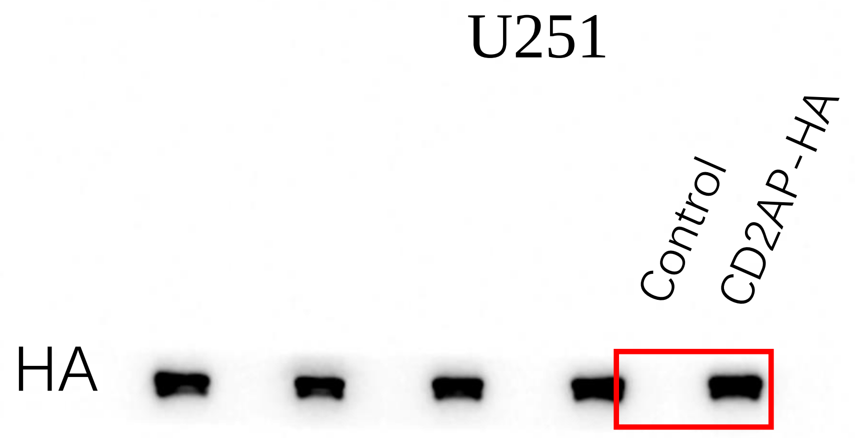

The membrane was imaged with Azure Biosystems 300

Full unedited gel for Figure 6F

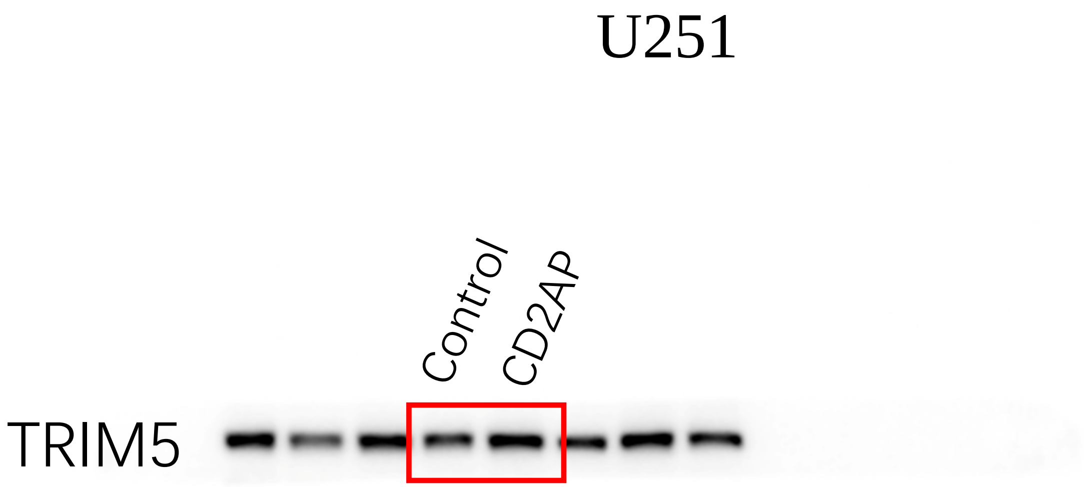

The membrane was imaged with Azure Biosystems 300

Full unedited gel for Figure 6F

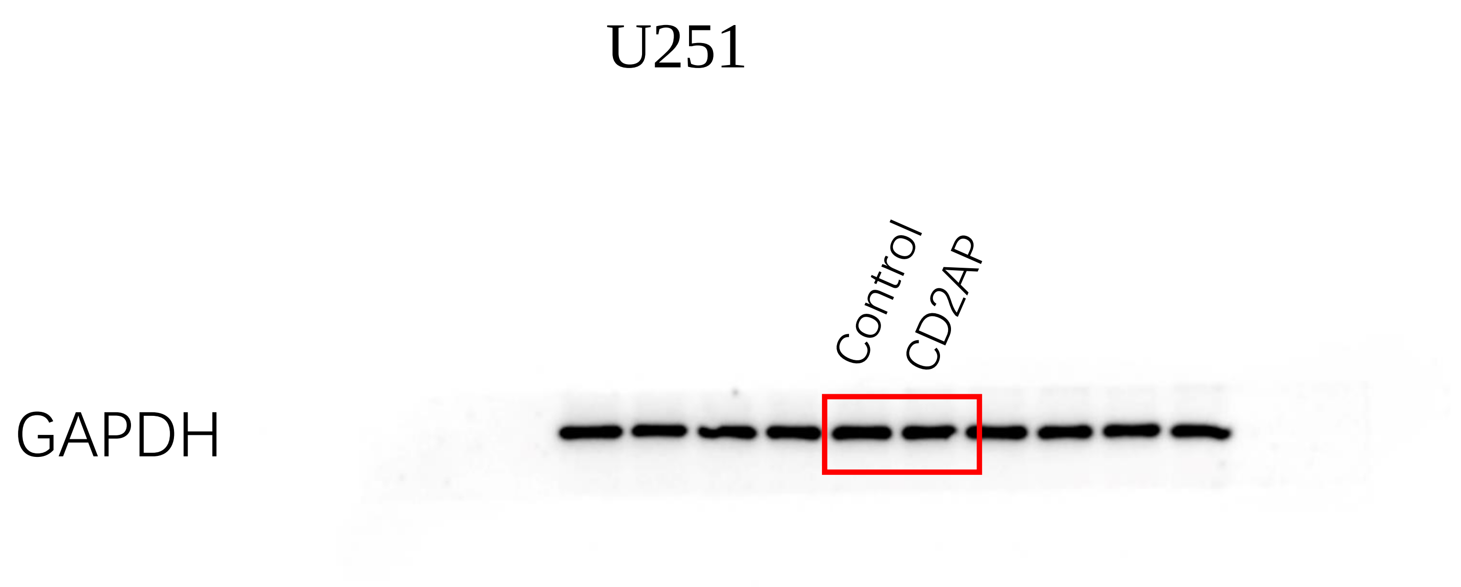

The membrane was imaged with Azure Biosystems 300

Full unedited gel for Figure 6H

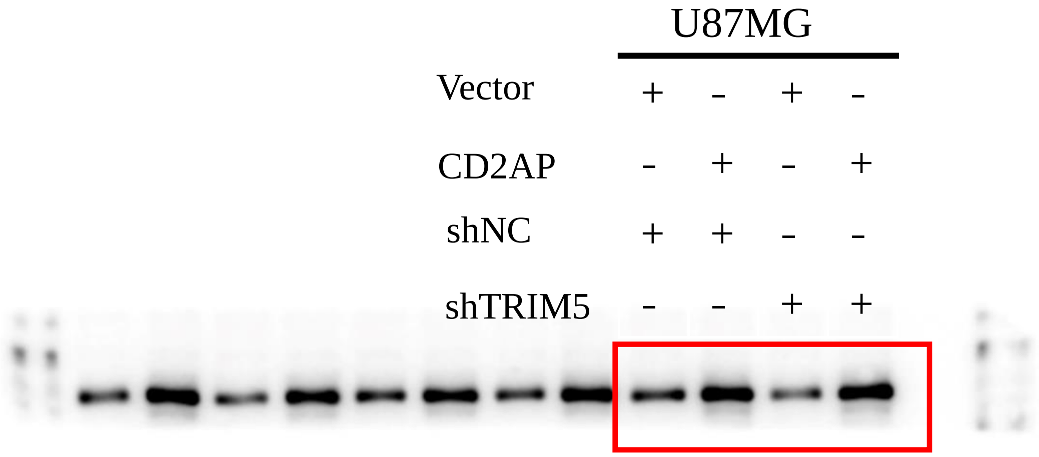

CD2AP

The membrane was imaged with Azure Biosystems 300

Full unedited gel for Figure 6H

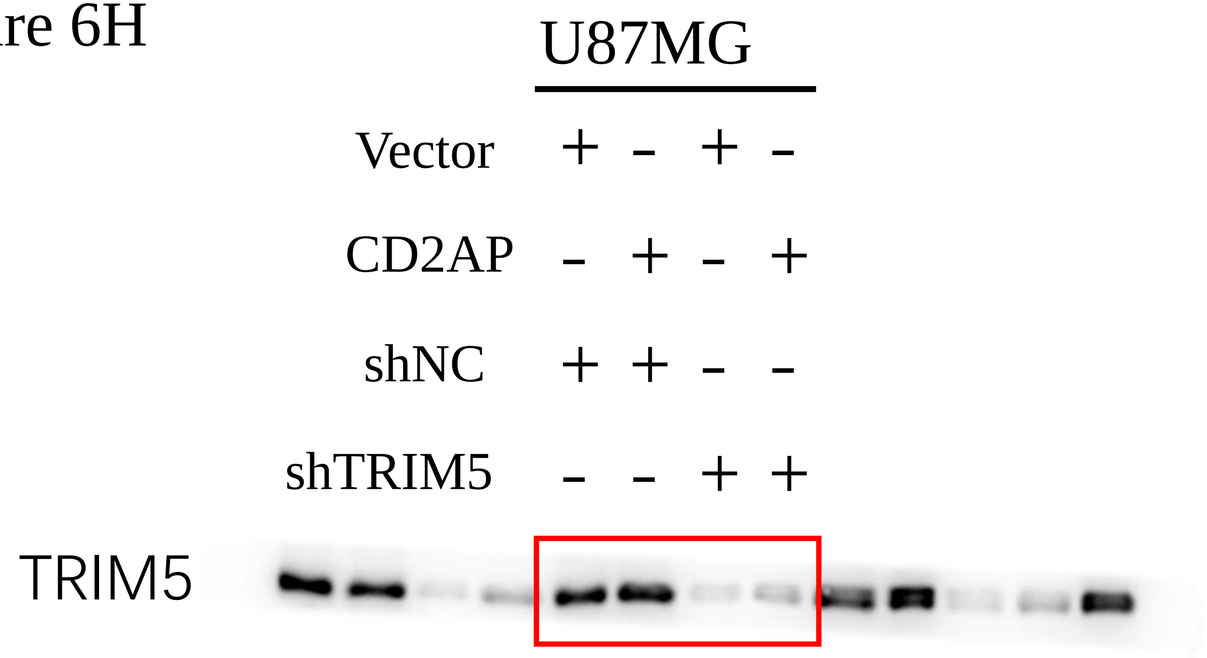

The membrane was imaged with Azure Biosystems 300

Full unedited gel for Figure 6H

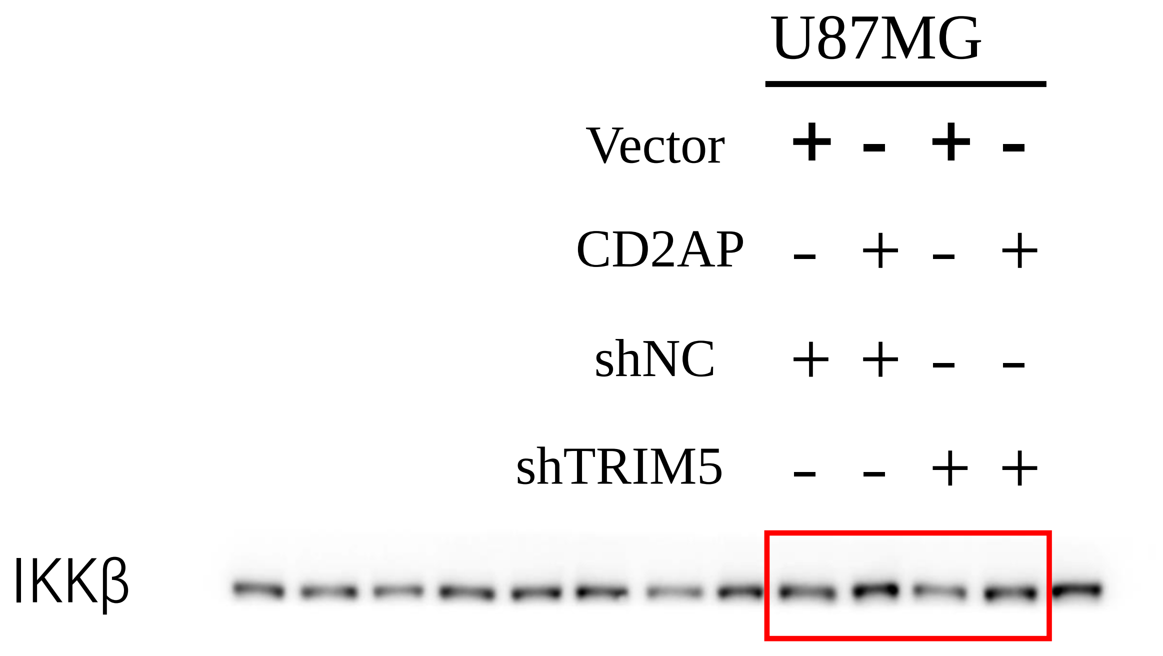

The membrane was imaged with Azure Biosystems 300

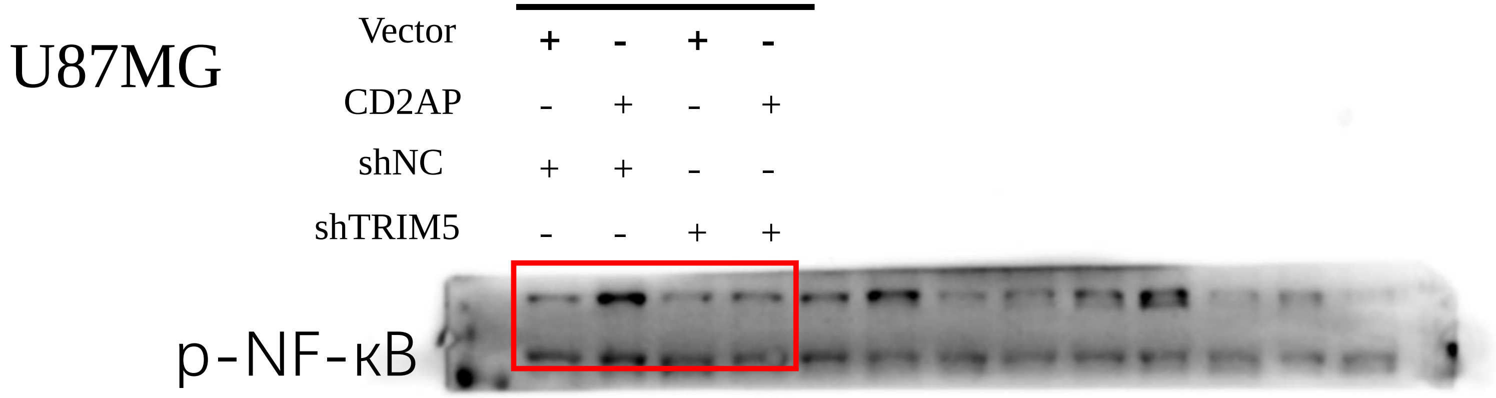

Full unedited gel for Figure 6H

The membrane was imaged with Azure Biosystems 300

Full unedited gel for Figure 6H

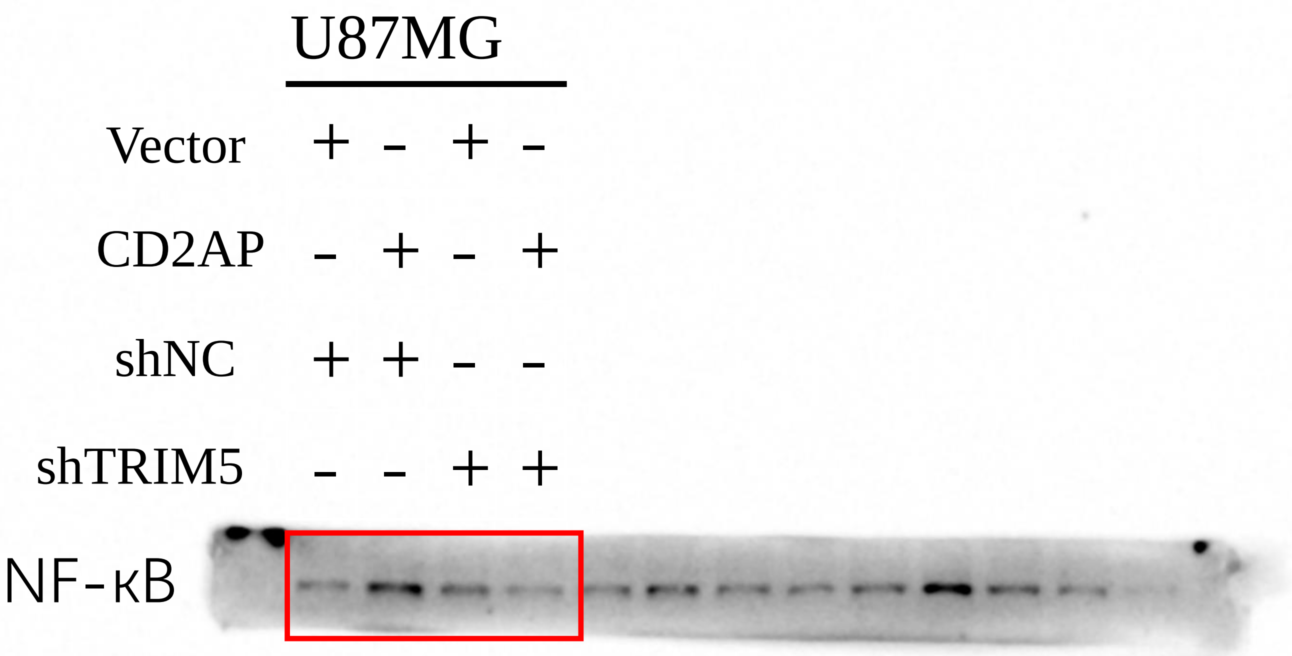

The membrane was imaged with Azure Biosystems 300

Full unedited gel for Figure 6H

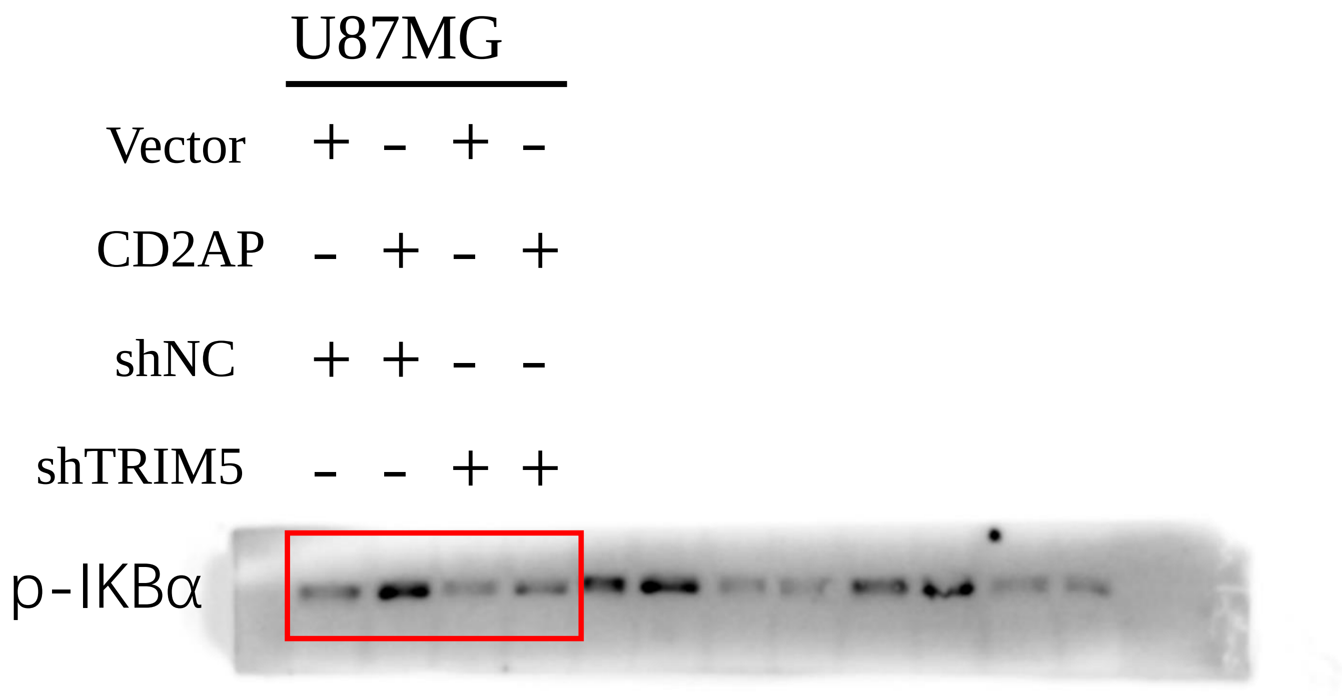

The membrane was imaged with Azure Biosystems 300

Full unedited gel for Figure 6H

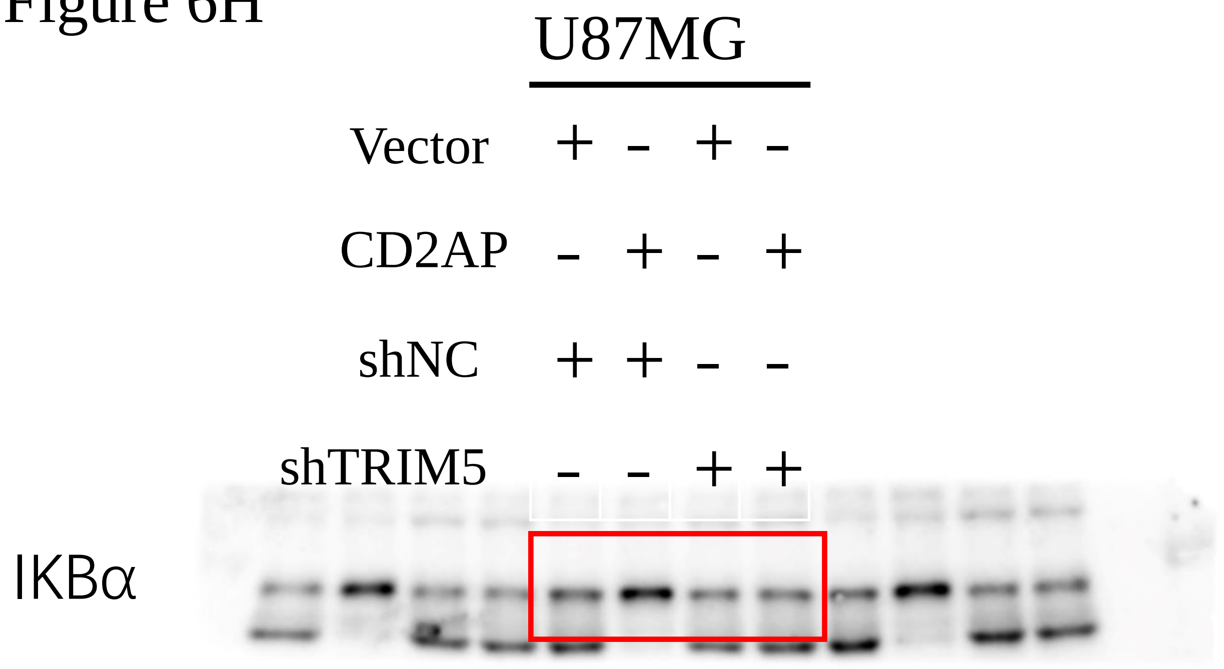

The membrane was imaged with Azure Biosystems 300

Full unedited gel for Figure 6H

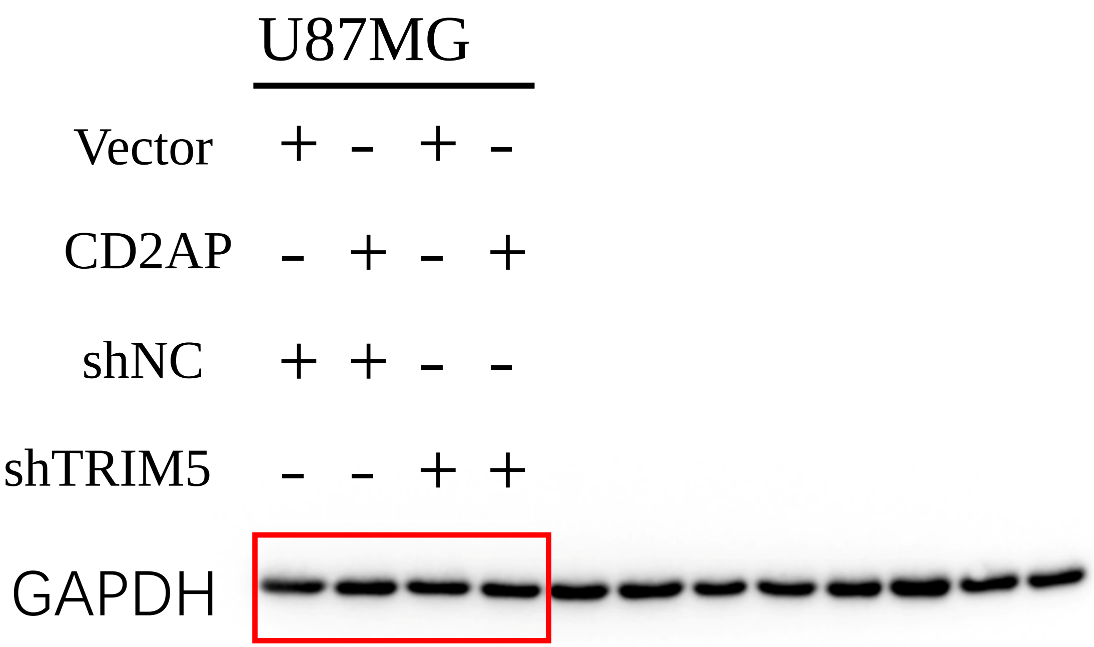

The membrane was imaged with Azure Biosystems 300

Full unedited gel for Figure 6H

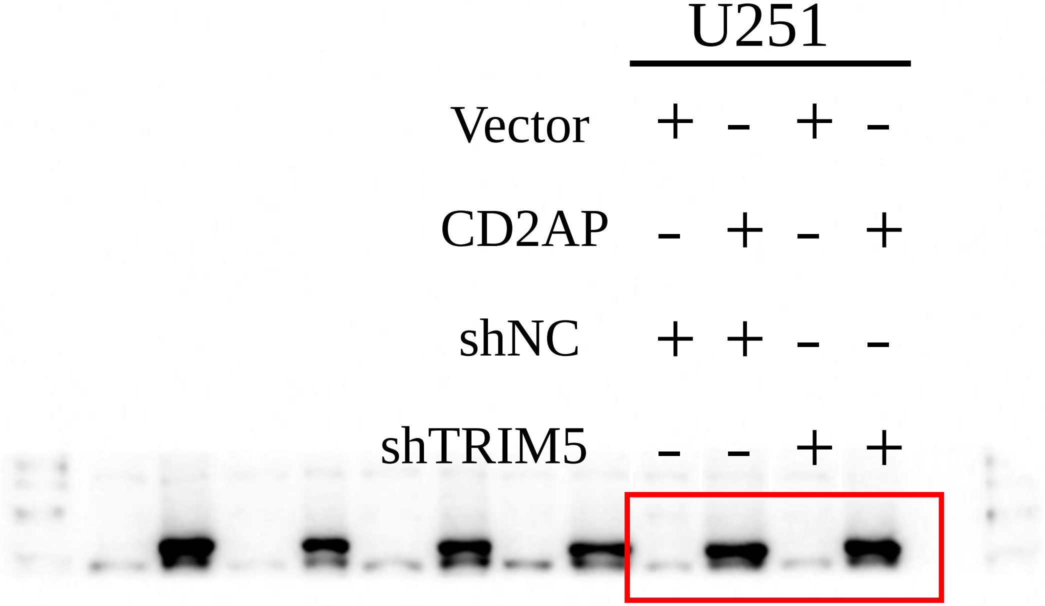

The membrane was imaged with Azure Biosystems 300

Full unedited gel for Figure 6H

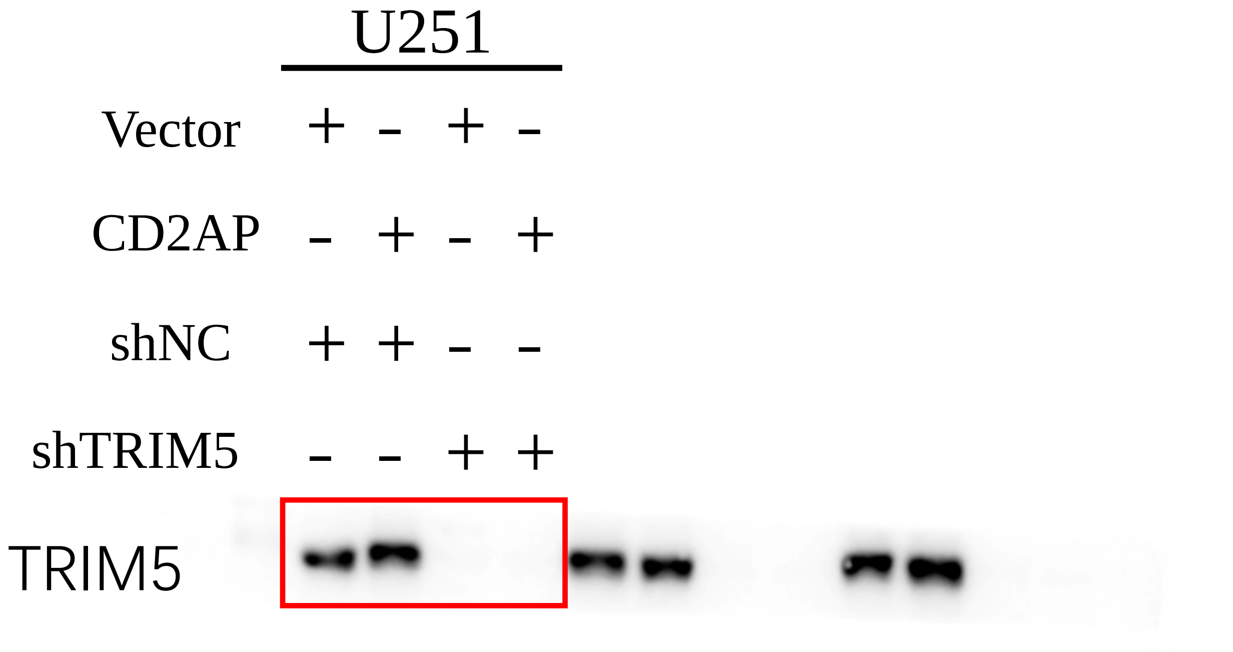

The membrane was imaged with Azure Biosystems 300

Full unedited gel for Figure 6H

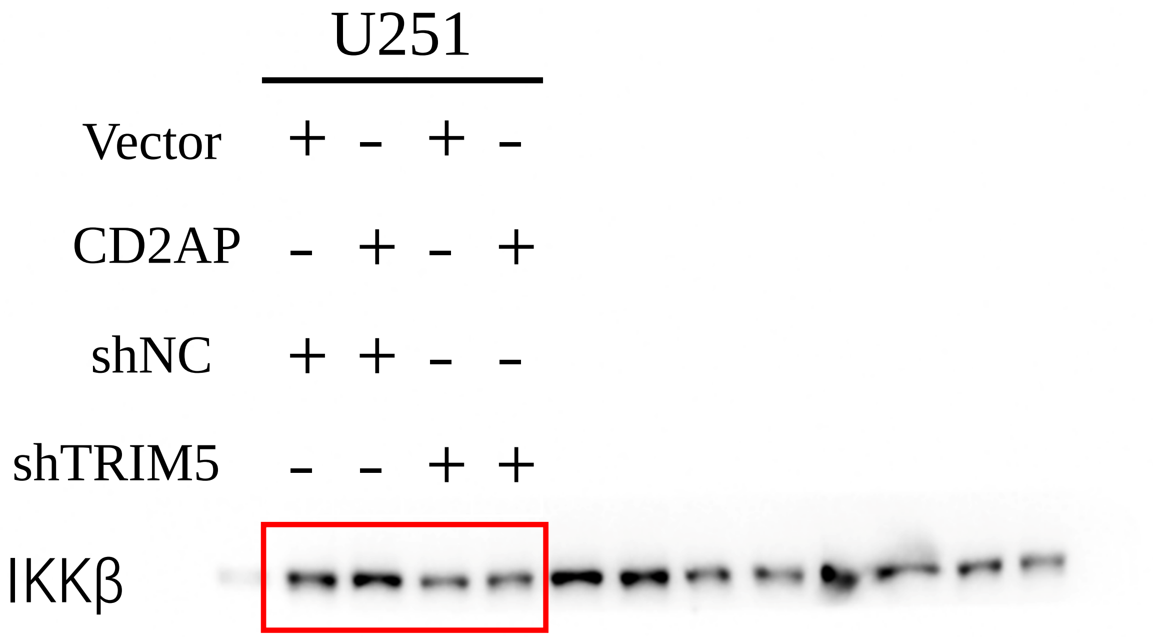

The membrane was imaged with Azure Biosystems 300

Full unedited gel for Figure 6H

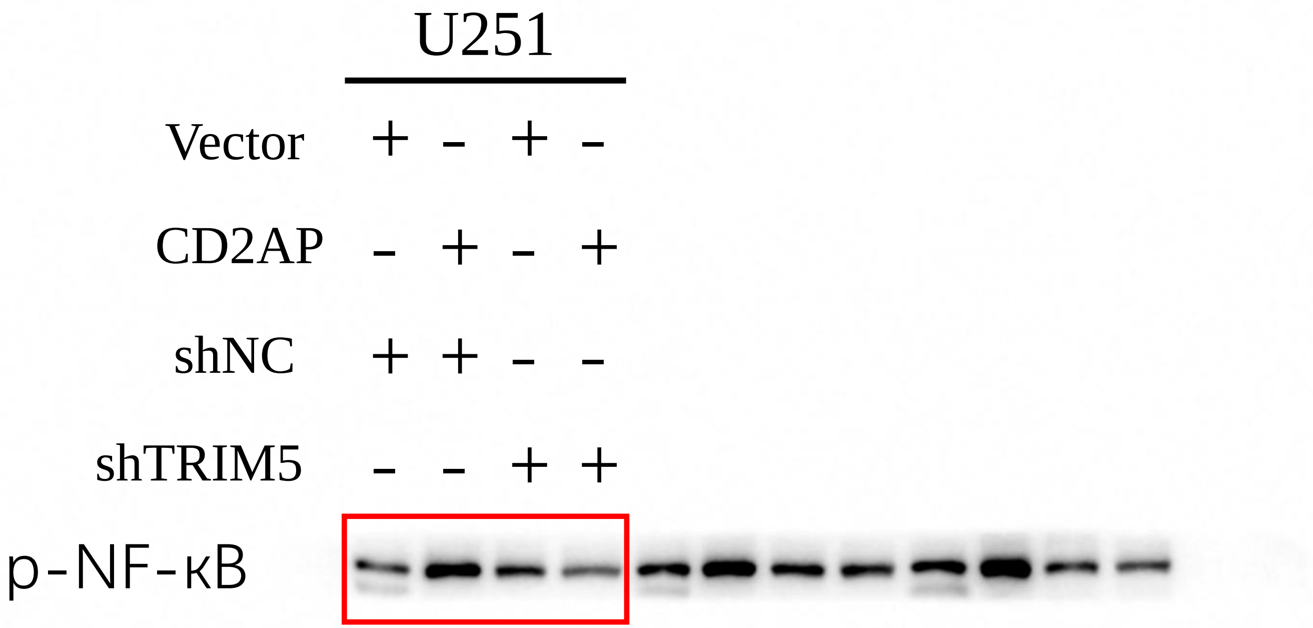

The membrane was imaged with Azure Biosystems 300

Full unedited gel for Figure 6H

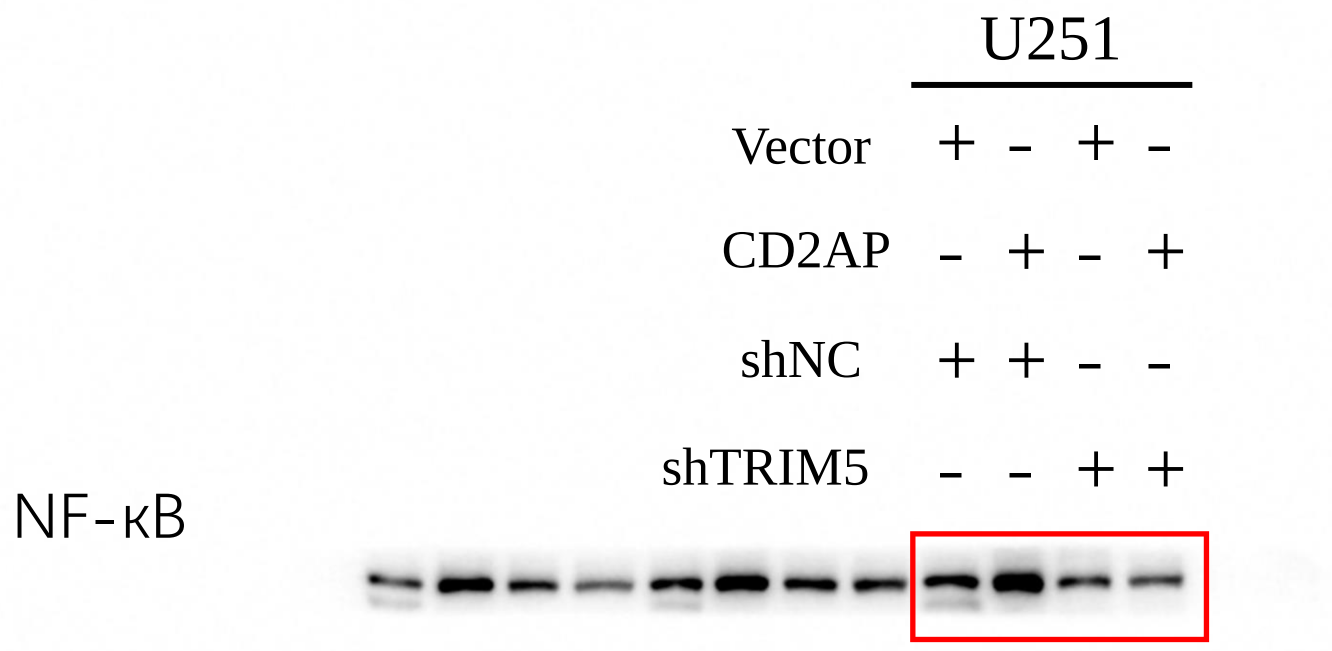

The membrane was imaged with Azure Biosystems 300

Full unedited gel for Figure 6H

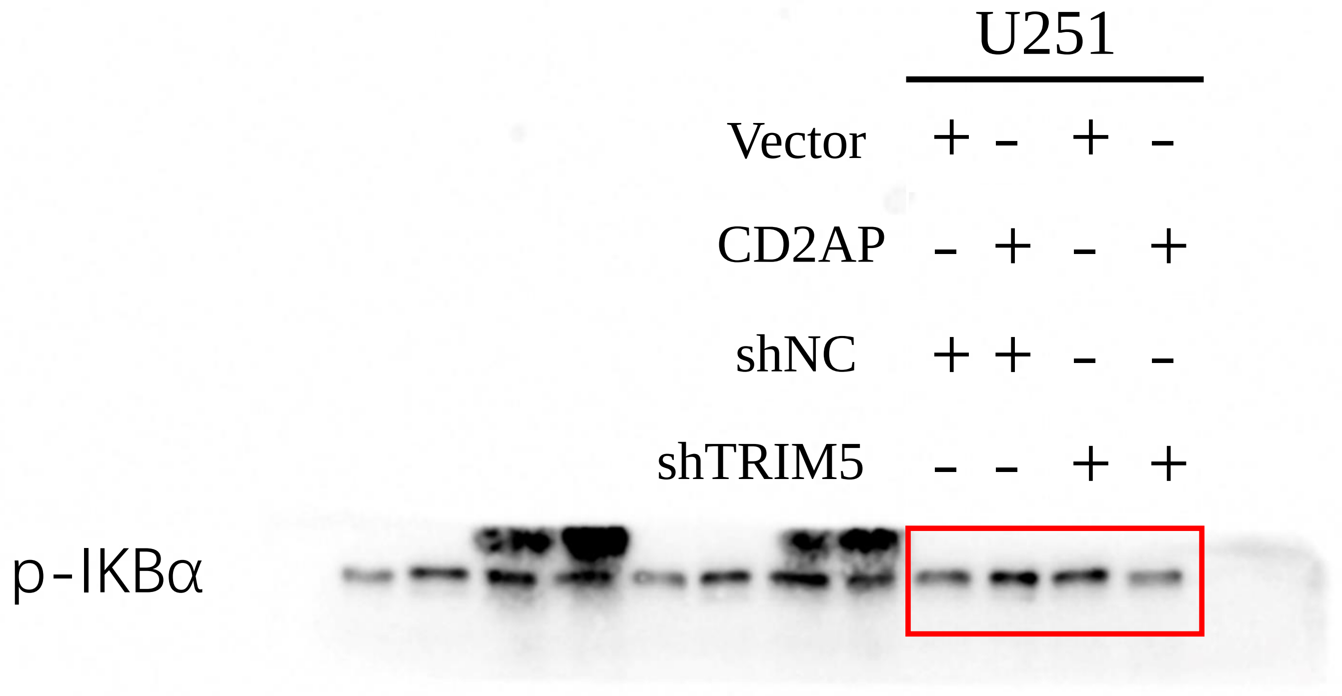

The membrane was imaged with Azure Biosystems 300

Full unedited gel for Figure 6H

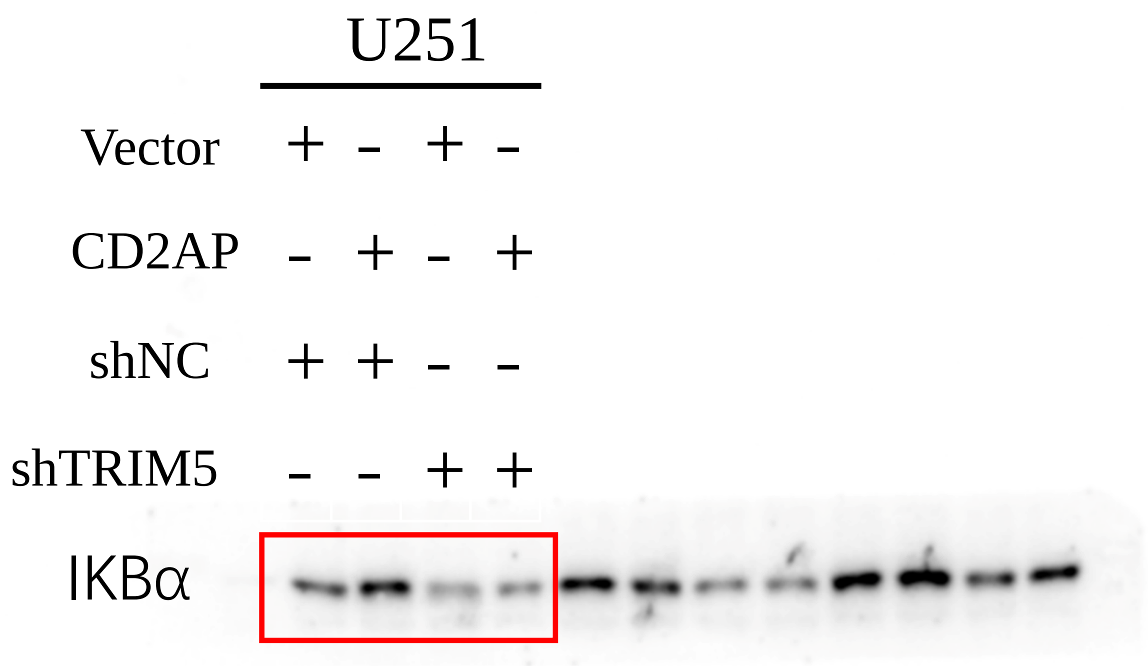

The membrane was imaged with Azure Biosystems 300

Full unedited gel for Figure 6H

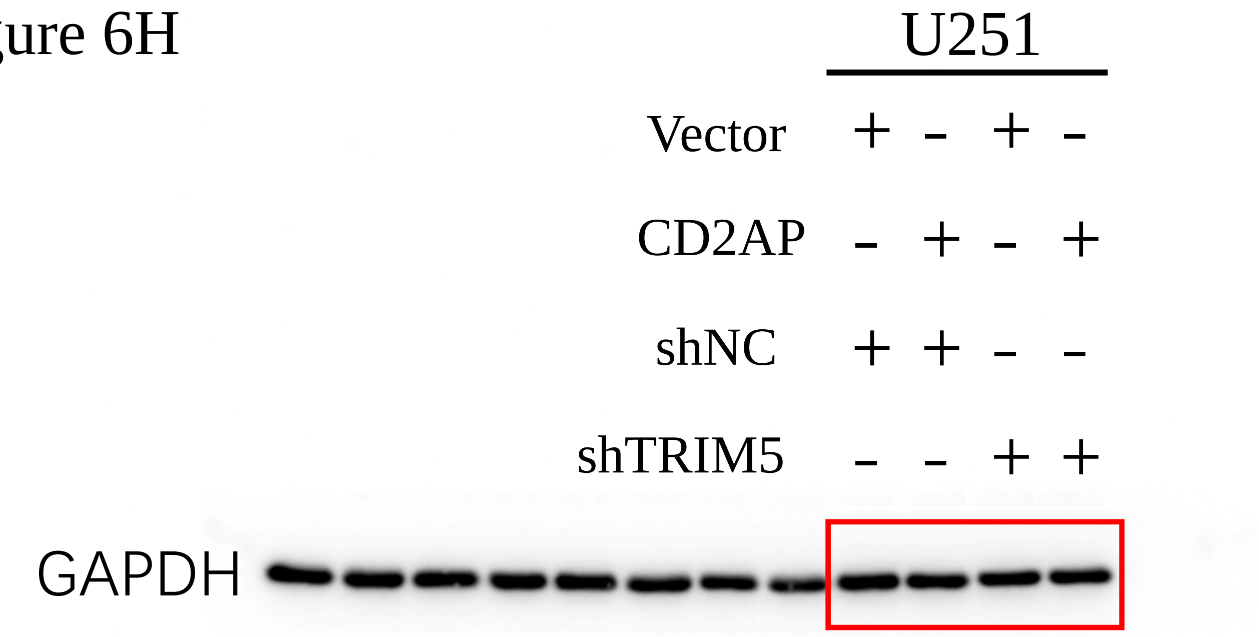

The membrane was imaged with Azure Biosystems 300

Full unedited gel for Figure Supplement 3C

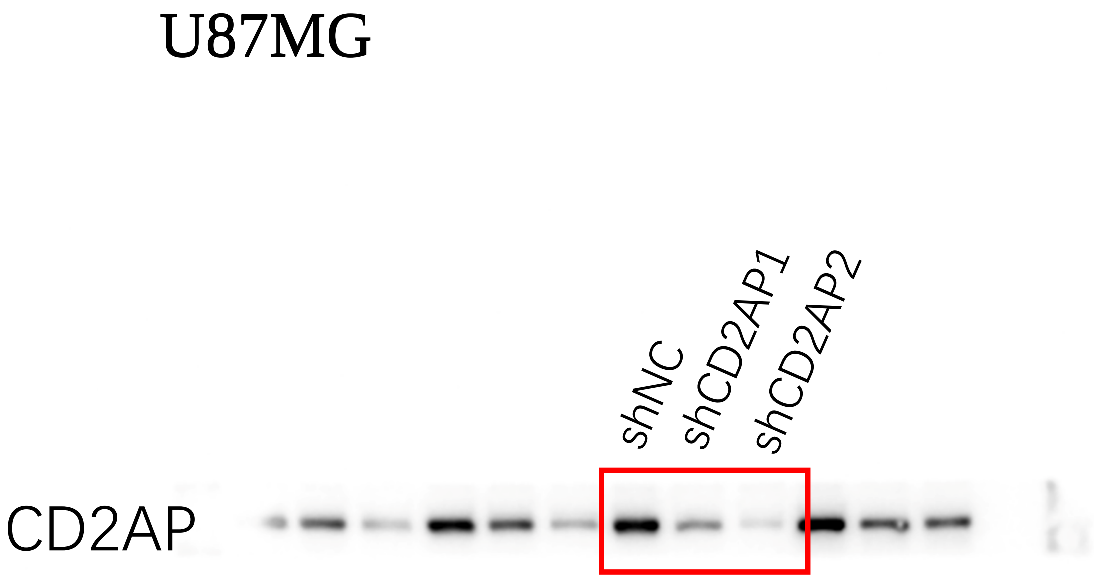

The membrane was imaged with Azure Biosystems 300

Full unedited gel for Figure Supplement 3C

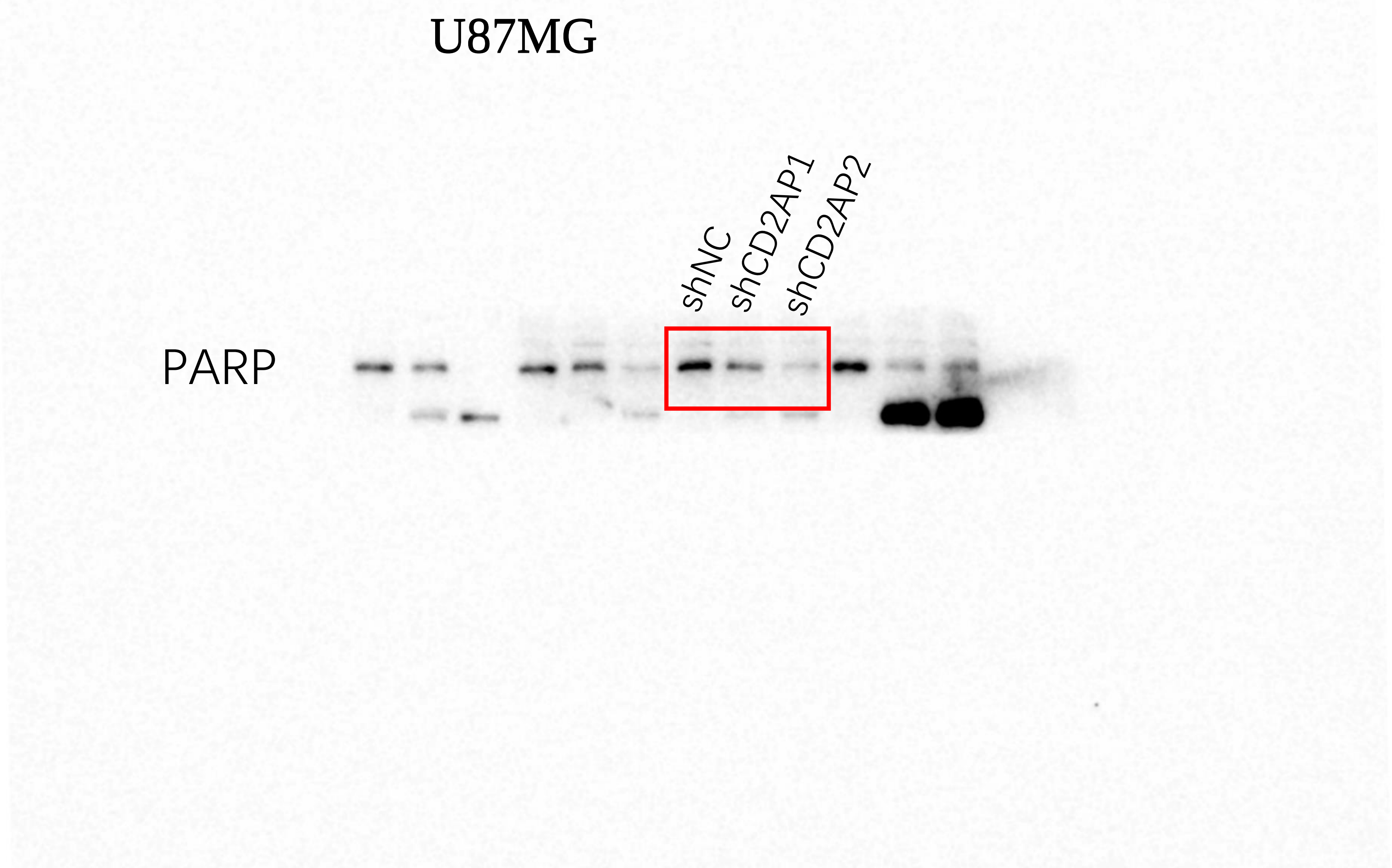

The membrane was imaged with Azure Biosystems 300

Full unedited gel for Figure Supplement 3C

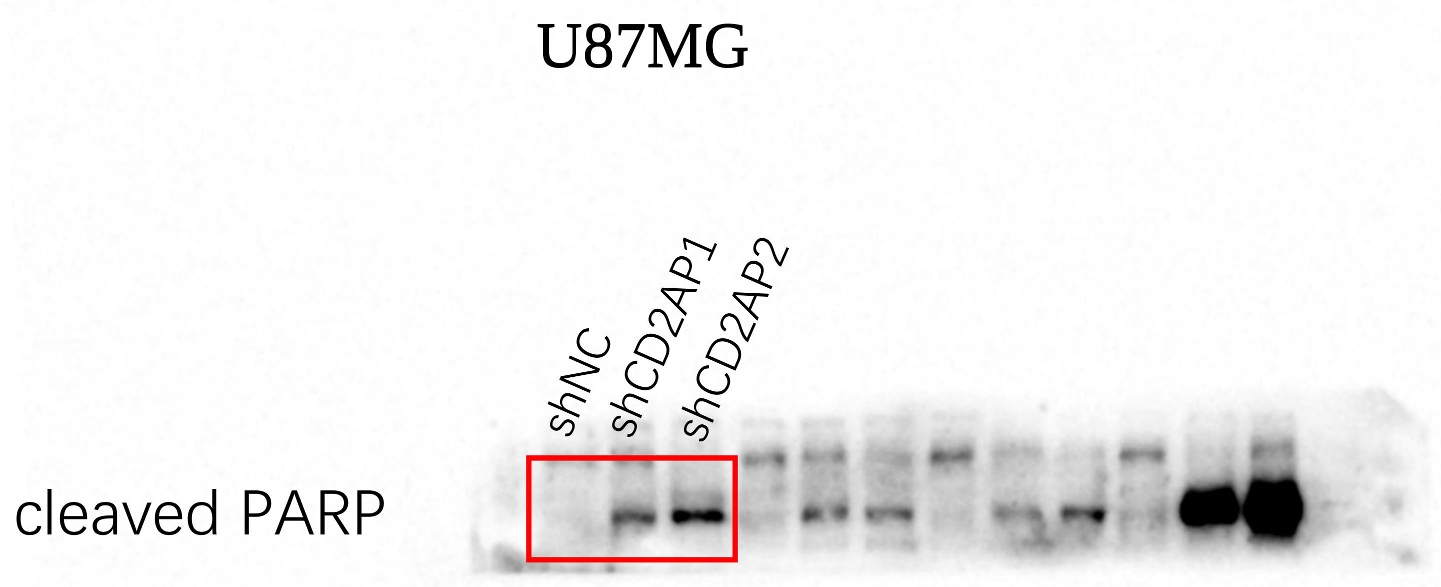

The membrane was imaged with Azure Biosystems 300

Full unedited gel for Figure Supplement 3C

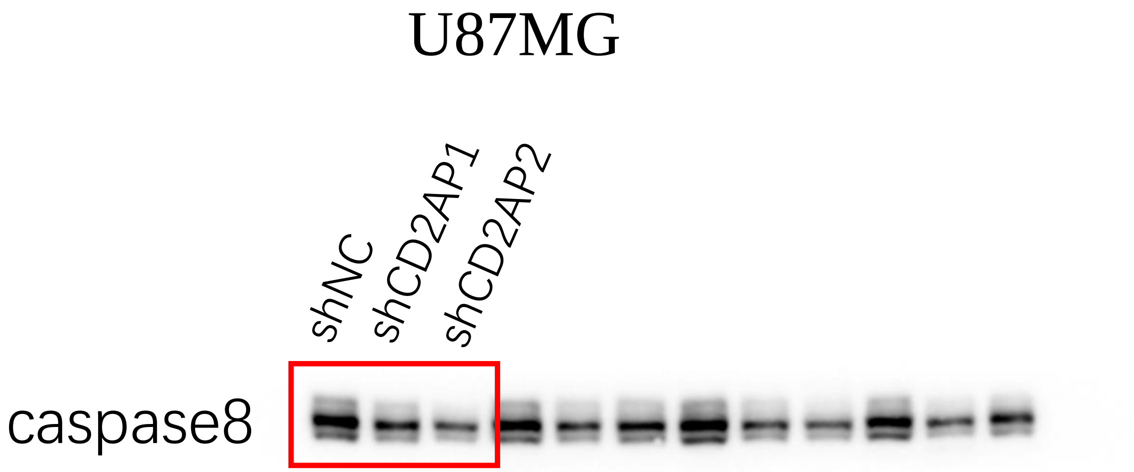

The membrane was imaged with Azure Biosystems 300

Full unedited gel for Figure Supplement 3C

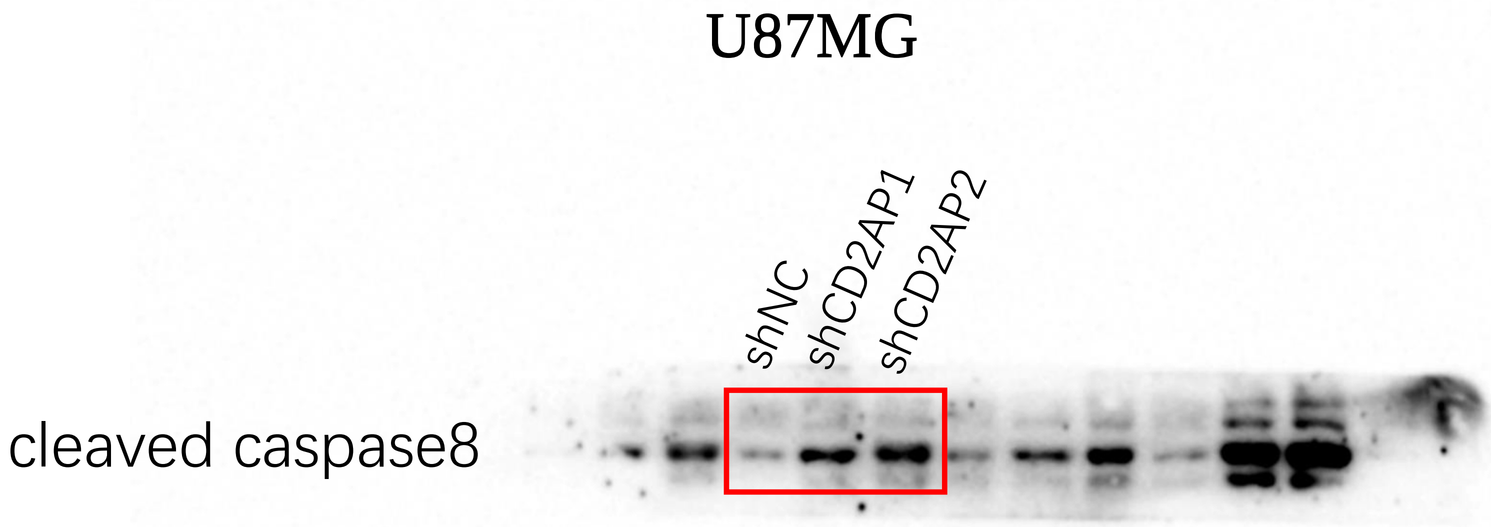

The membrane was imaged with Azure Biosystems 300

Full unedited gel for Figure Supplement 3C

U87MG

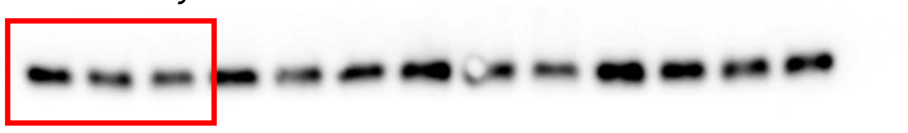

The membrane was imaged with Azure Biosystems 300

Full unedited gel for Figure Supplement 3C

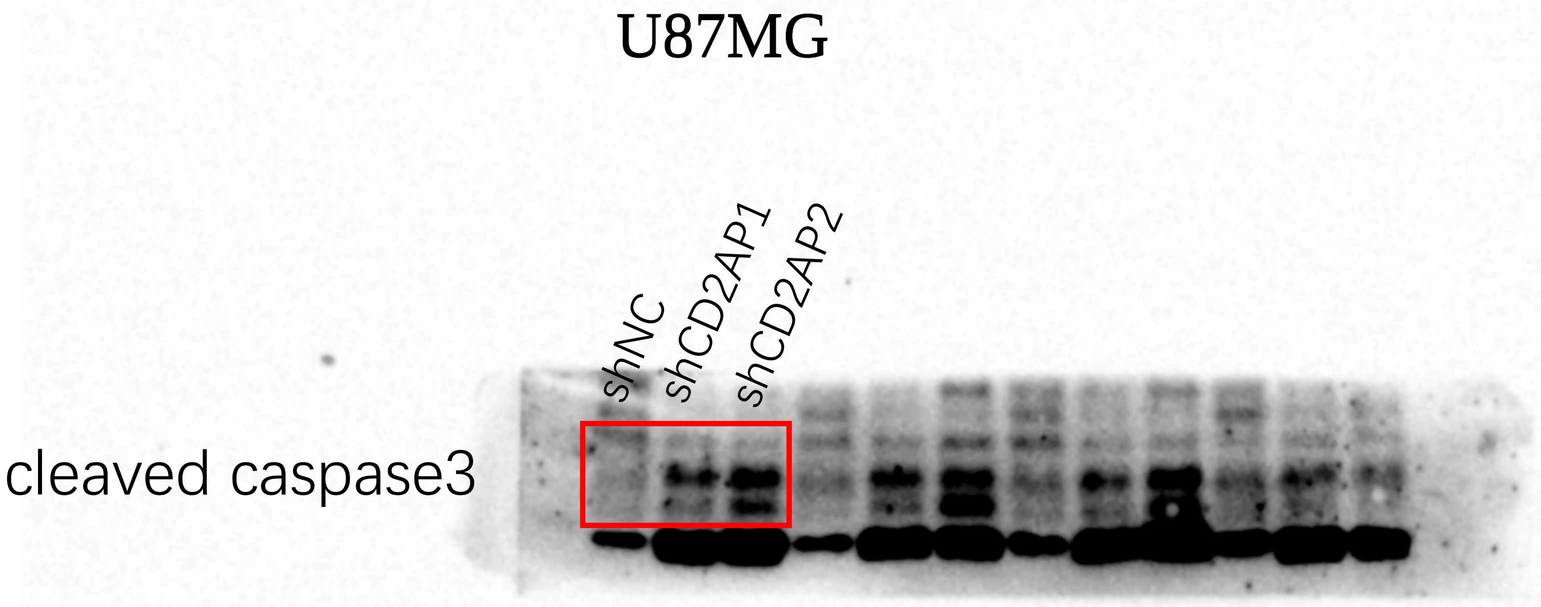

The membrane was imaged with Azure Biosystems 300

Full unedited gel for Figure Supplement 3C

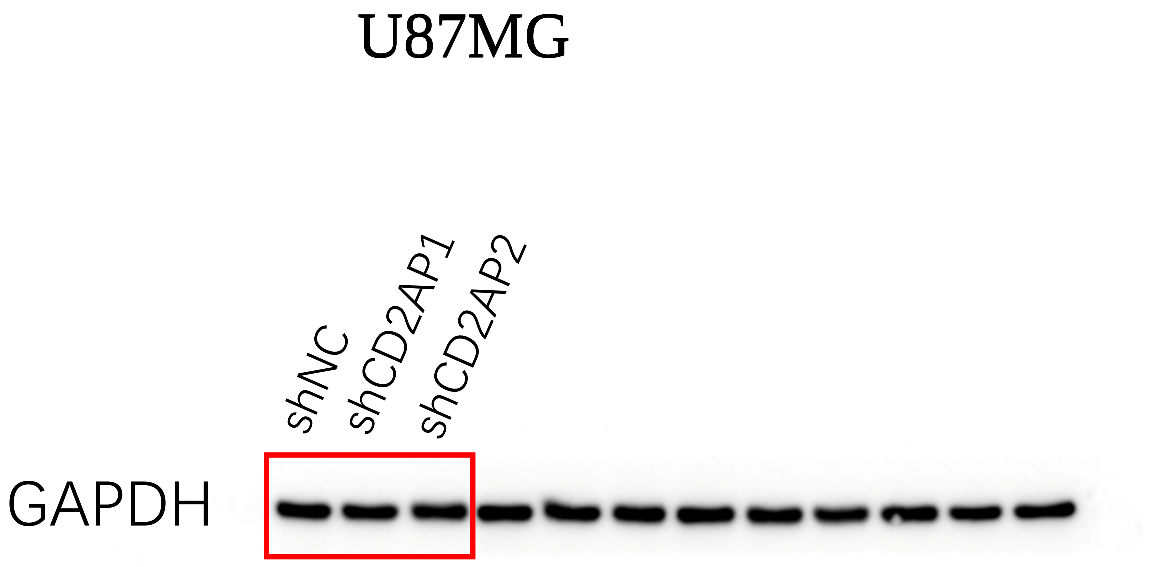

The membrane was imaged with Azure Biosystems 300

Full unedited gel for Figure Supplement 3C

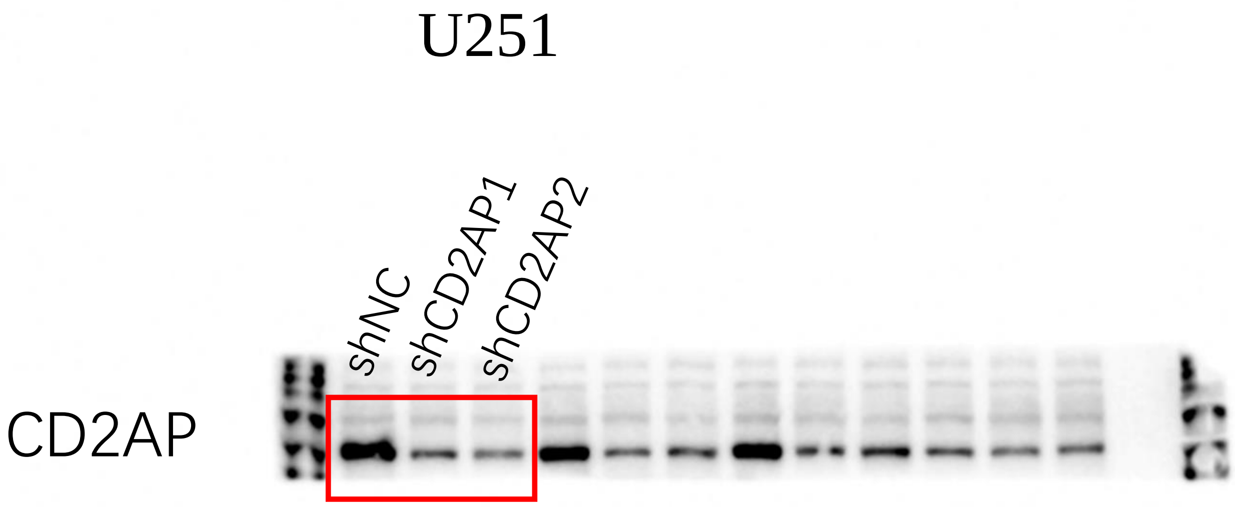

The membrane was imaged with Azure Biosystems 300

Full unedited gel for Figure Supplement 3C

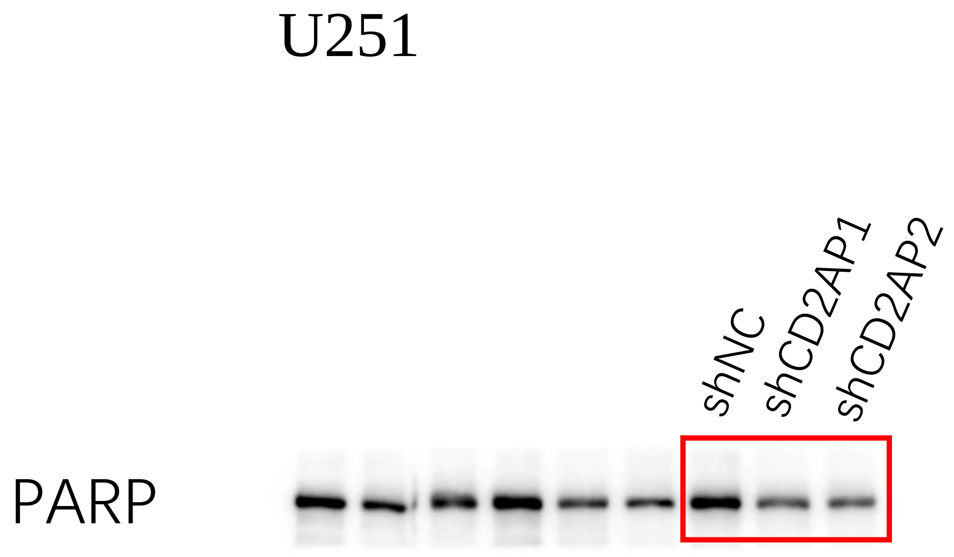

The membrane was imaged with Azure Biosystems 300

Full unedited gel for Figure Supplement 3C

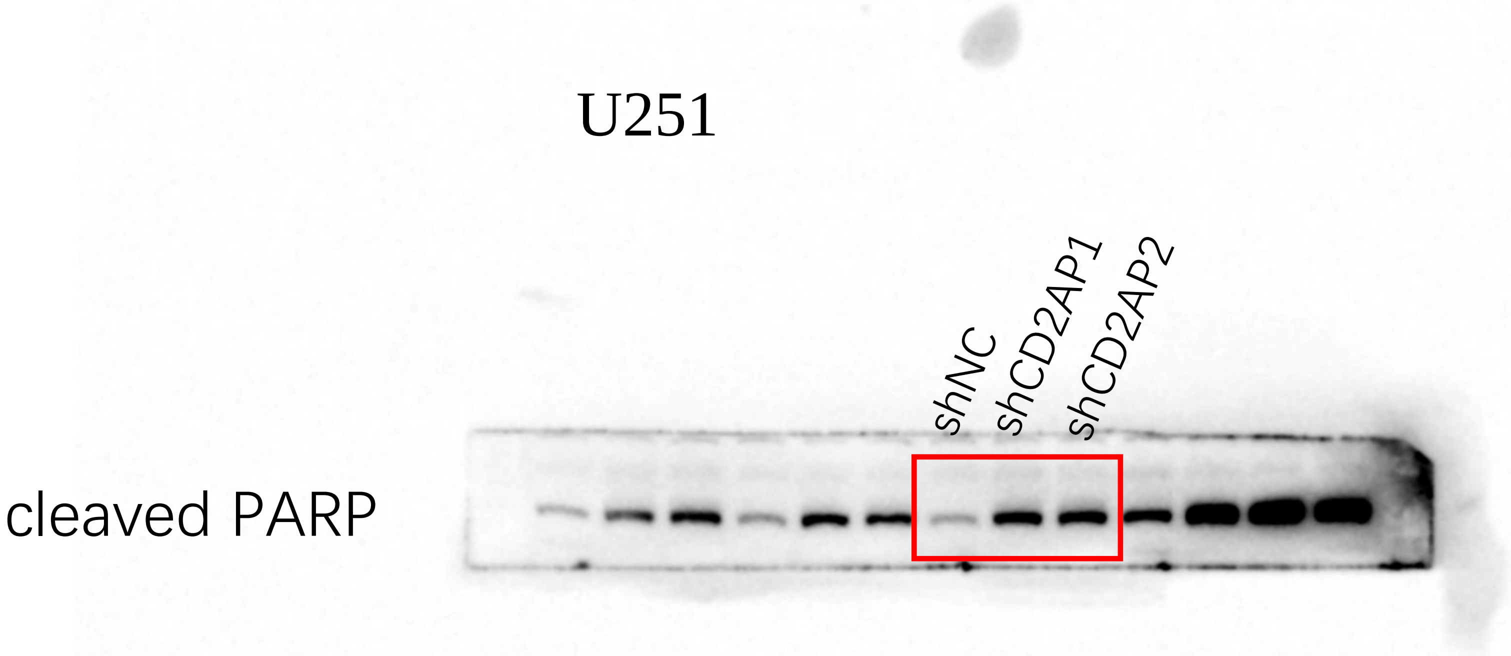

The membrane was imaged with Azure Biosystems 300

Full unedited gel for Figure Supplement 3C

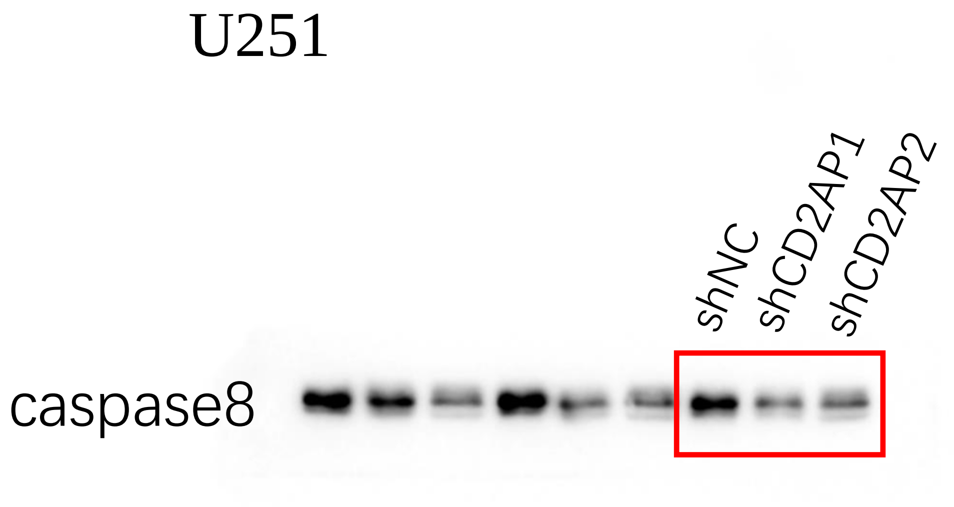

The membrane was imaged with Azure Biosystems 300

Full unedited gel for Figure Supplement 3C

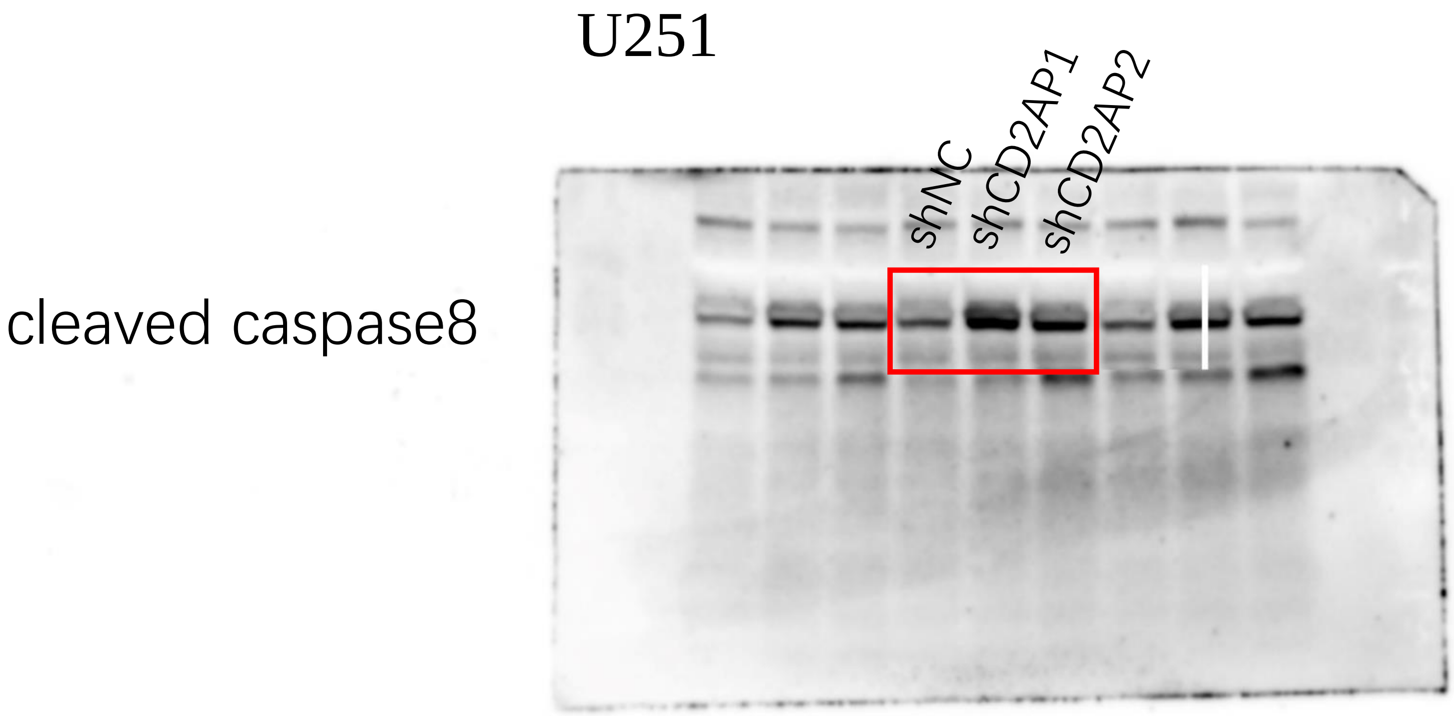

The membrane was imaged with Azure Biosystems 300

Full unedited gel for Figure Supplement 3C

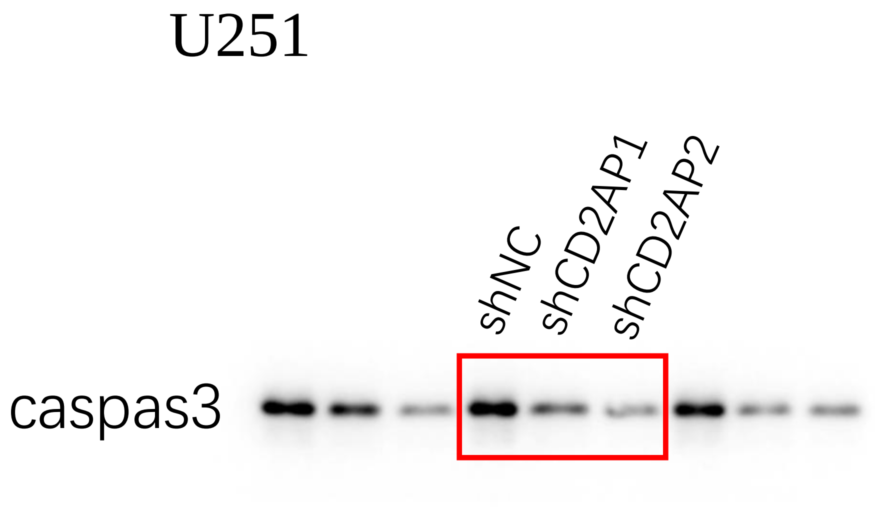

The membrane was imaged with Azure Biosystems 300

Full unedited gel for Figure Supplement 3C

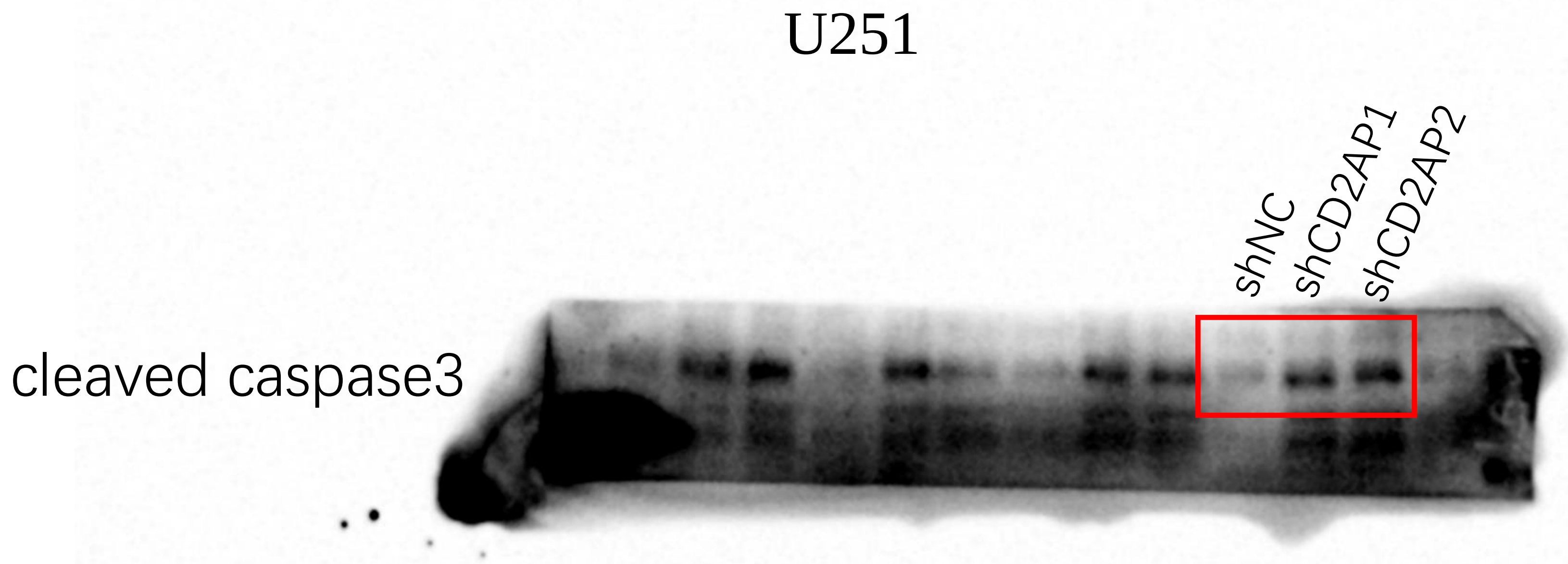

The membrane was imaged with Azure Biosystems 300

Full unedited gel for Figure Supplement 3C

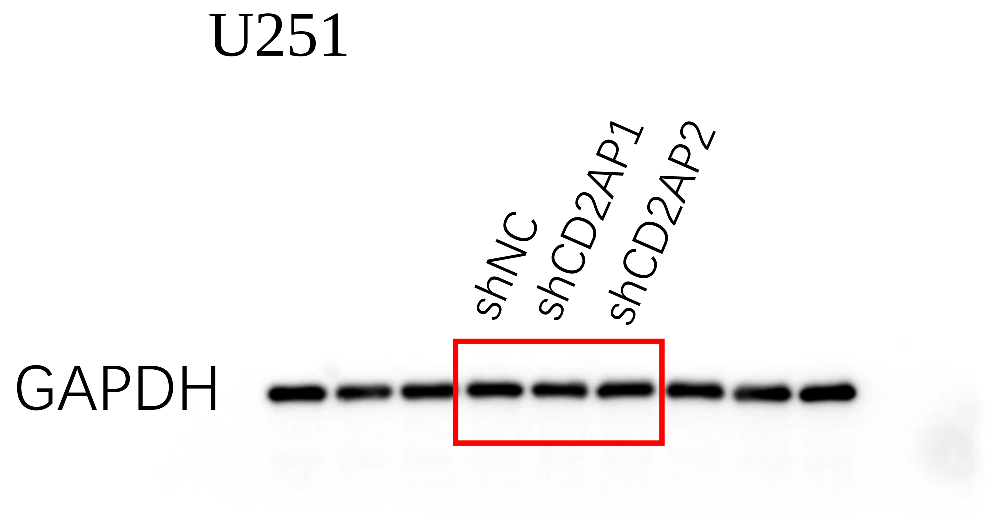

The membrane was imaged with Azure Biosystems 300

Full unedited gel for Figure Supplement 4A

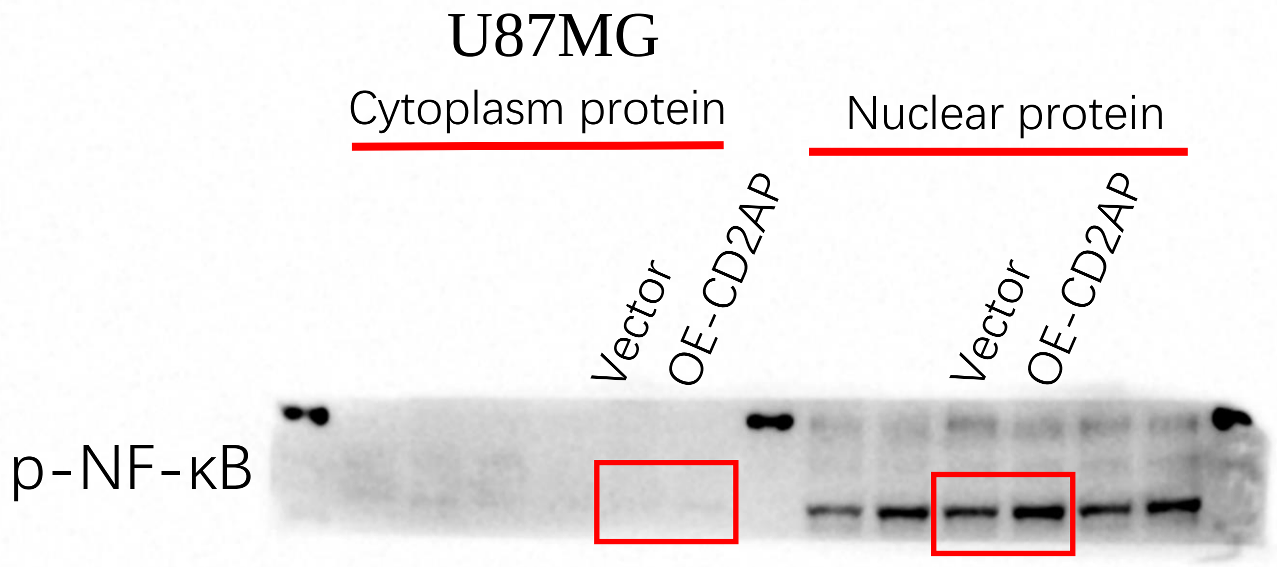

The membrane was imaged with Azure Biosystems 300

Full unedited gel for Figure Supplement 4A

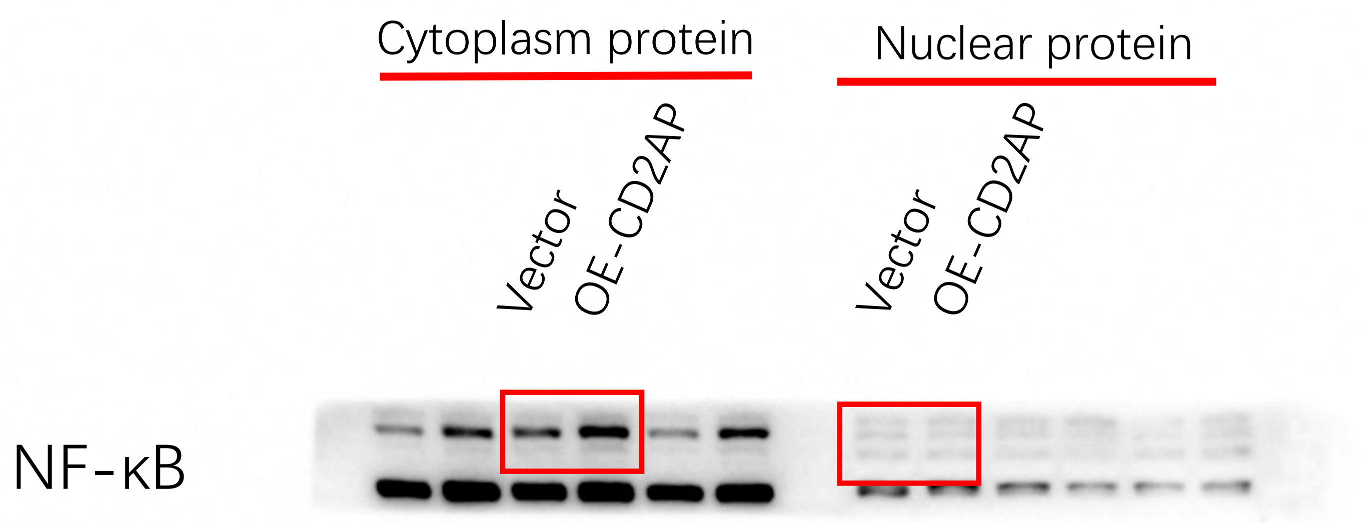

The membrane was imaged with Azure Biosystems 300

Full unedited gel for Figure Supplement 4A

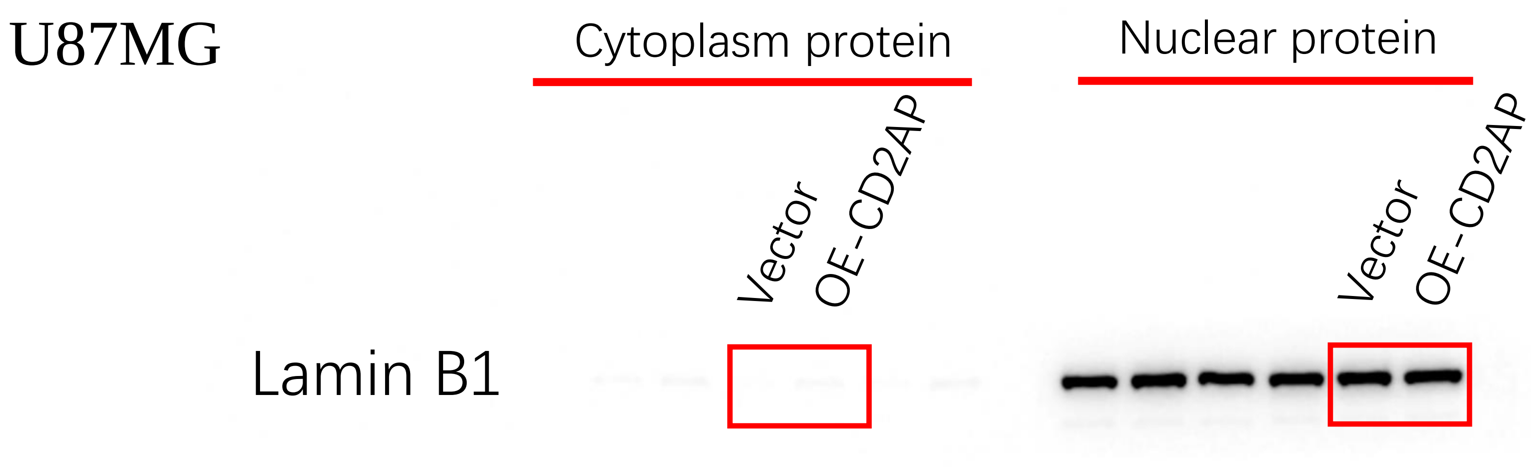

The membrane was imaged with Azure Biosystems 300

Full unedited gel for Figure Supplement 4A

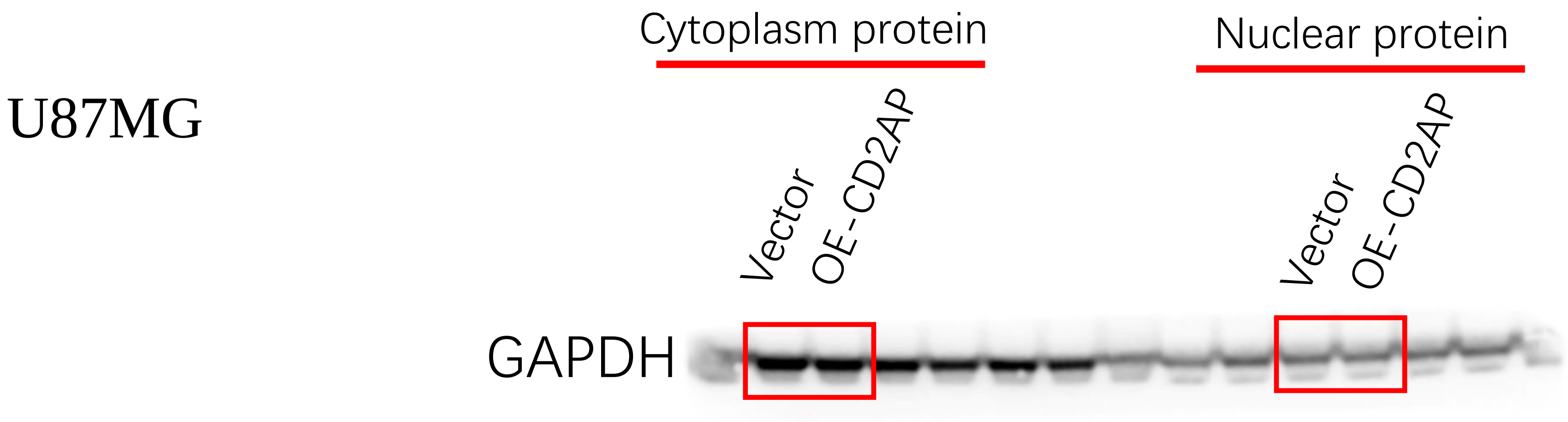

The membrane was imaged with Azure Biosystems 300

Full unedited gel for Figure Supplement 4A

U251

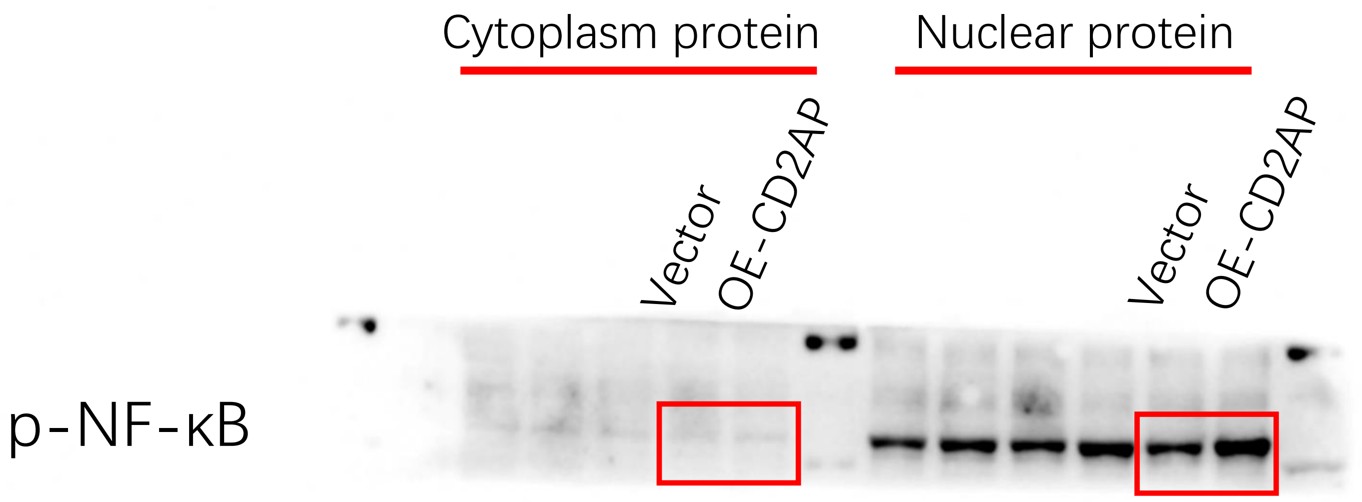

The membrane was imaged with Azure Biosystems 300

Full unedited gel for Figure Supplement 4A

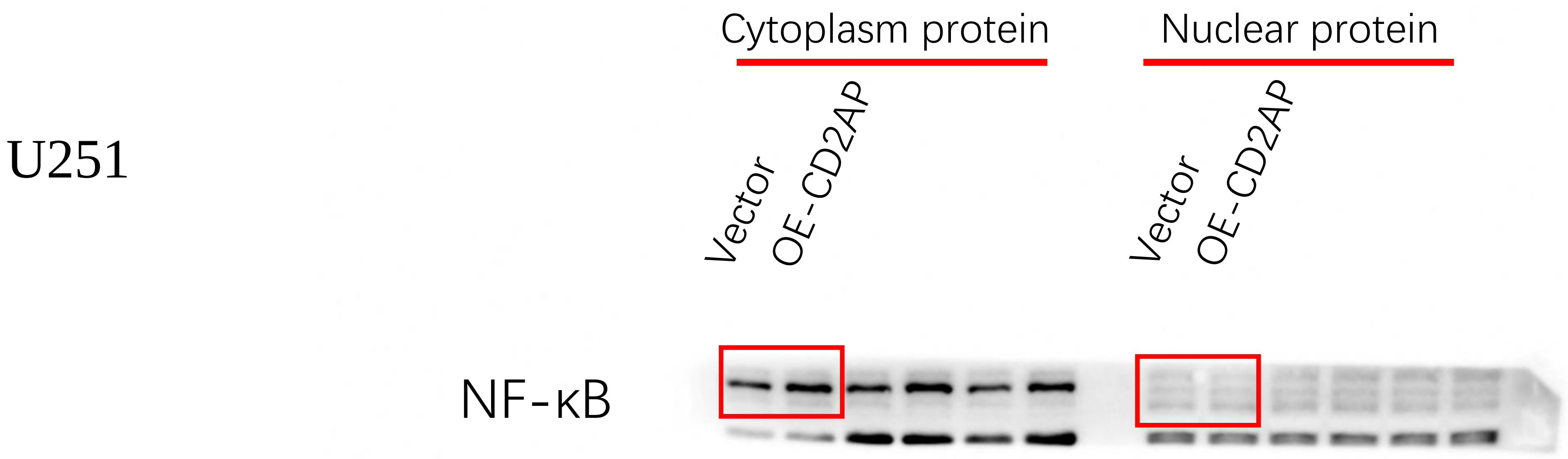

The membrane was imaged with Azure Biosystems 300

Full unedited gel for Figure Supplement 4A

U251

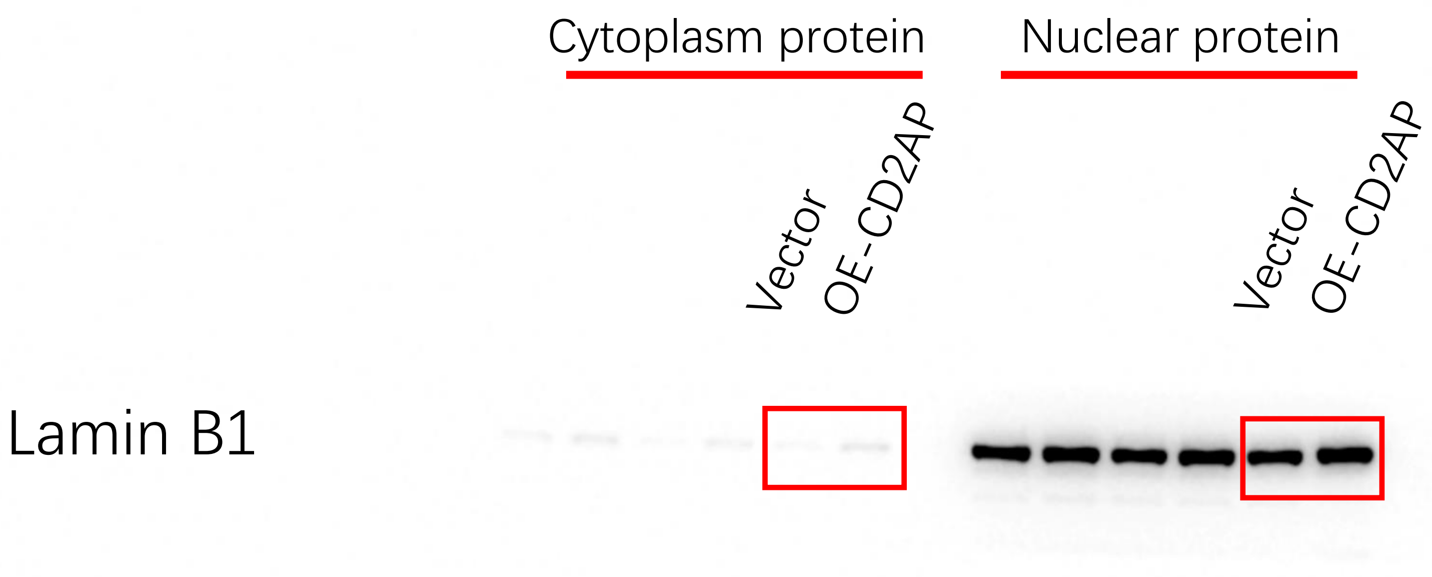

The membrane was imaged with Azure Biosystems 300

Full unedited gel for Figure Supplement 4A

U251

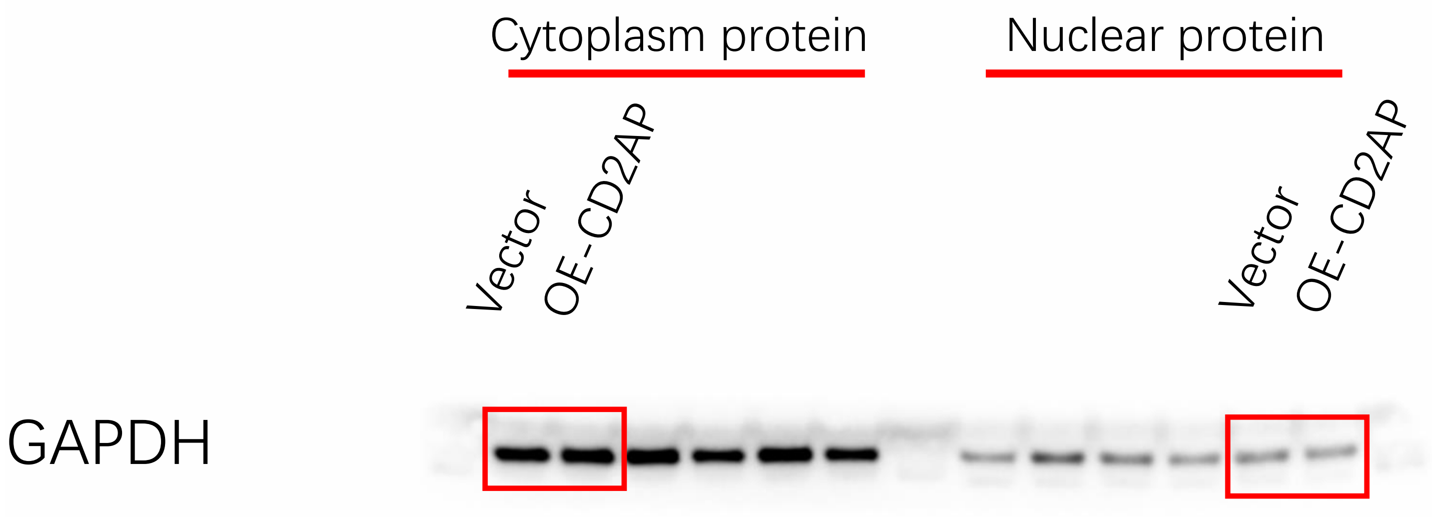

The membrane was imaged with Azure Biosystems 300

Full unedited gel for Figure Supplement 5G

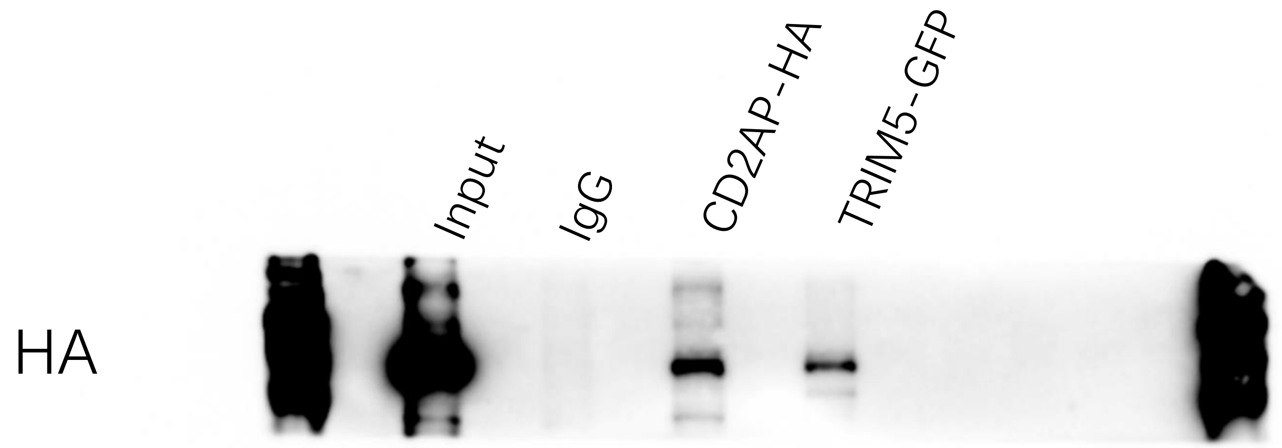

The membrane was imaged with Azure Biosystems 300

Full unedited gel for Figure Supplement 5G

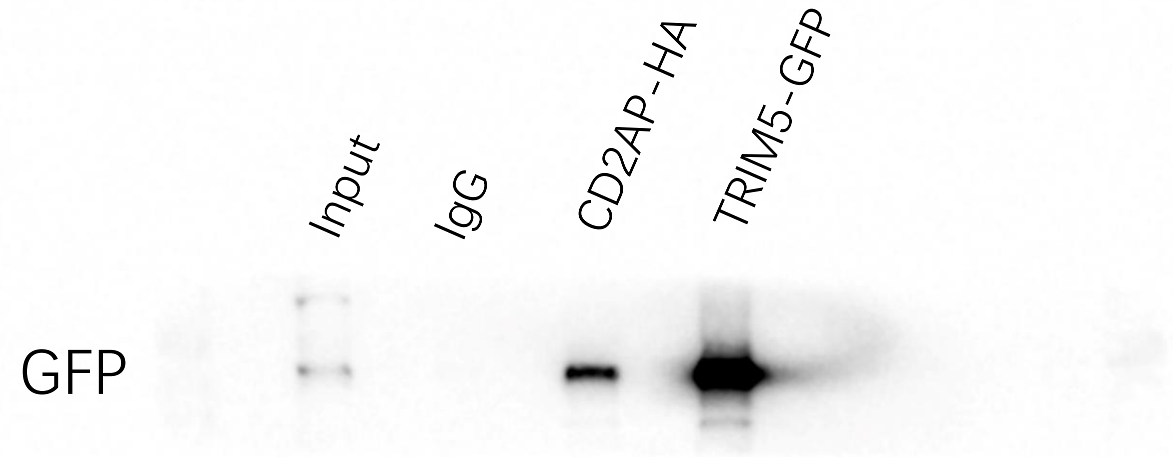

The membrane was imaged with Azure Biosystems 300

Full unedited gel for Figure Supplement 6A

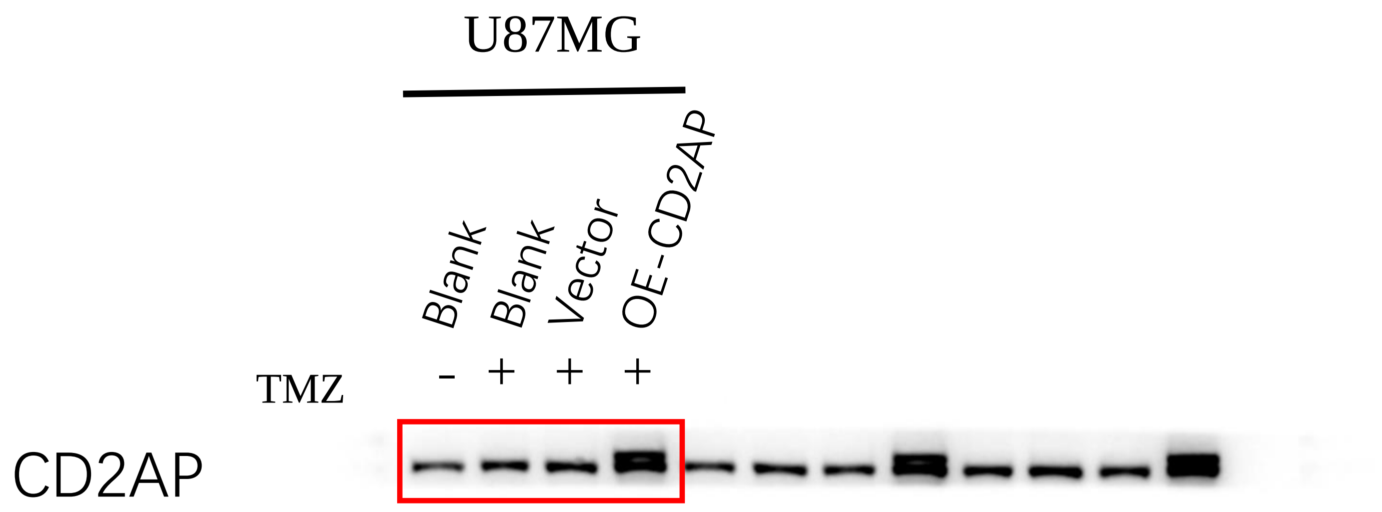

The membrane was imaged with Azure Biosystems 300

Full unedited gel for Figure Supplement 6A

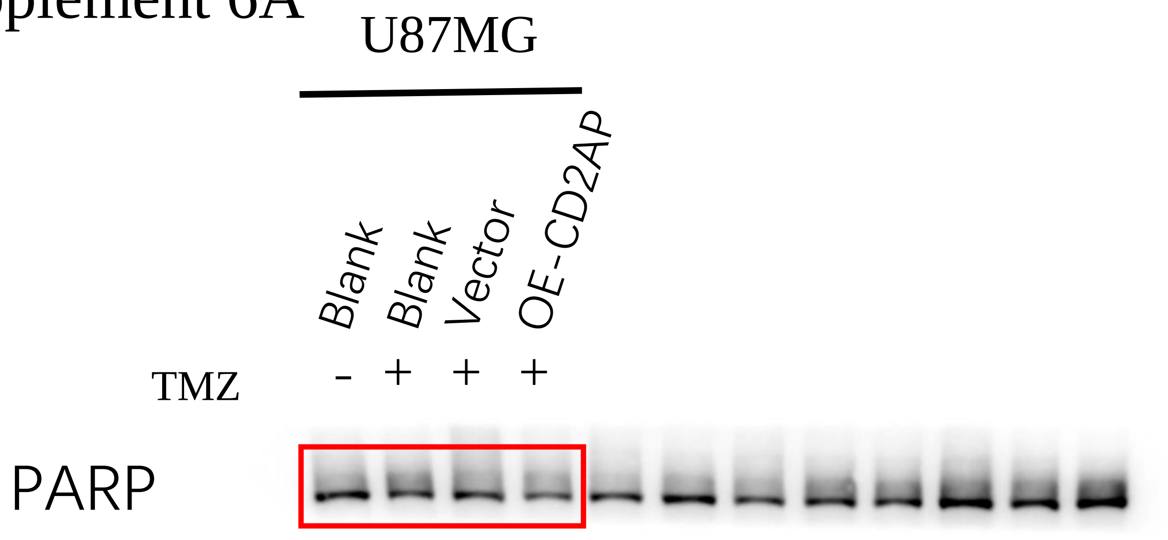

The membrane was imaged with Azure Biosystems 300

Full unedited gel for Figure Supplement 6A

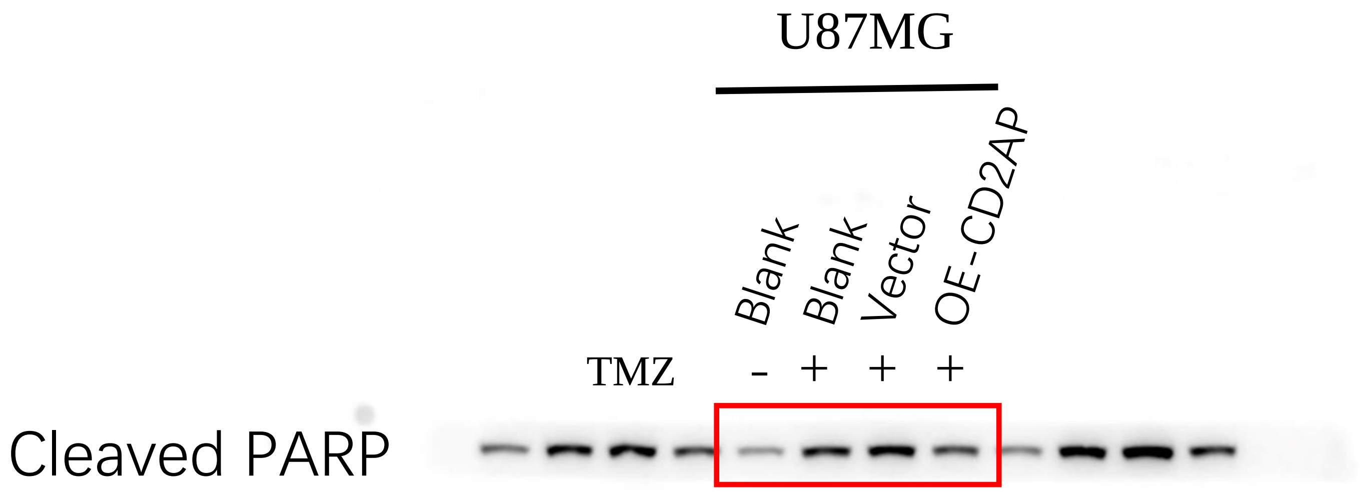

The membrane was imaged with Azure Biosystems 300

Full unedited gel for Figure Supplement 6A

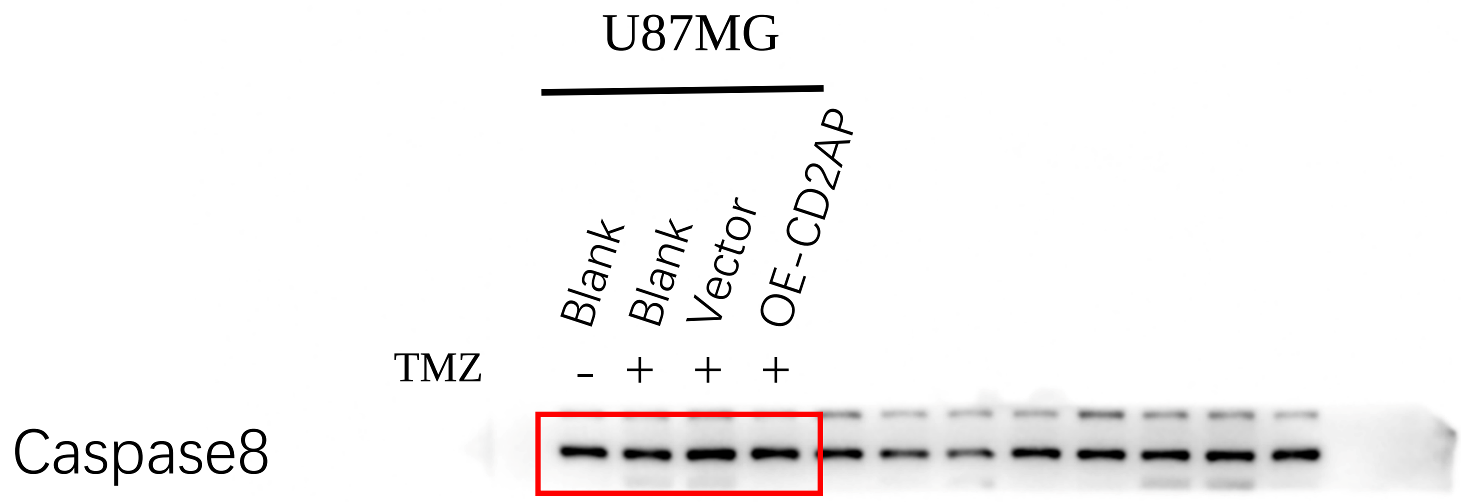

The membrane was imaged with Azure Biosystems 300

Full unedited gel for Figure Supplement 6A

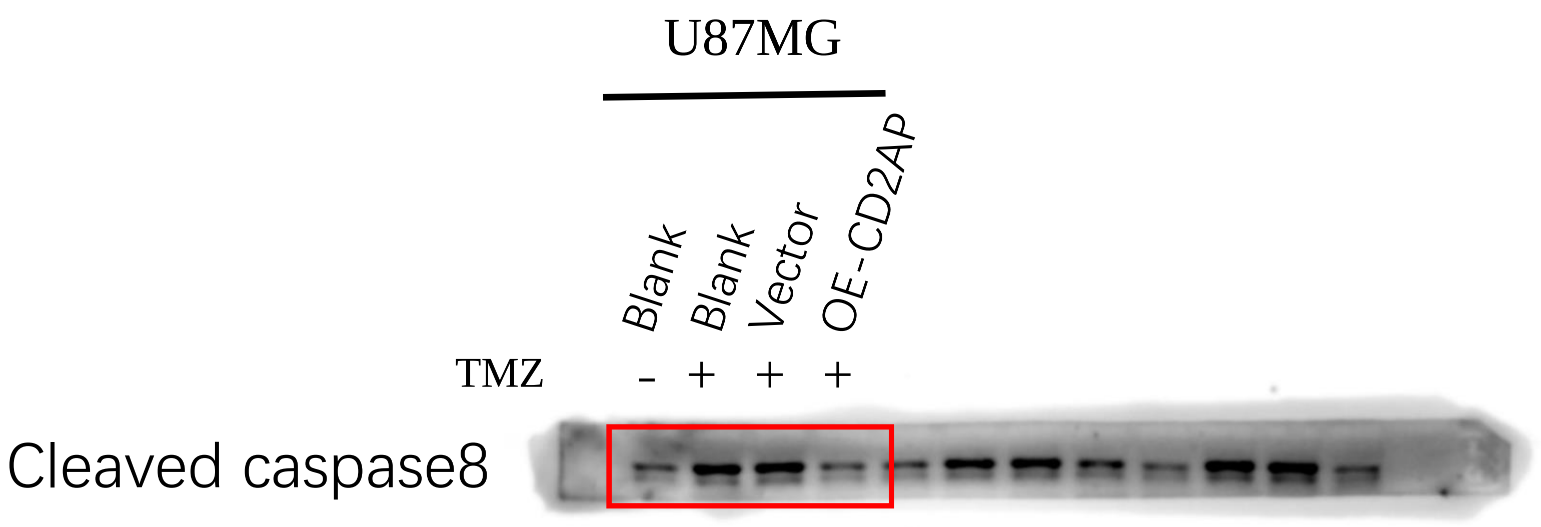

The membrane was imaged with Azure Biosystems 300

Full unedited gel for Figure Supplement 6A

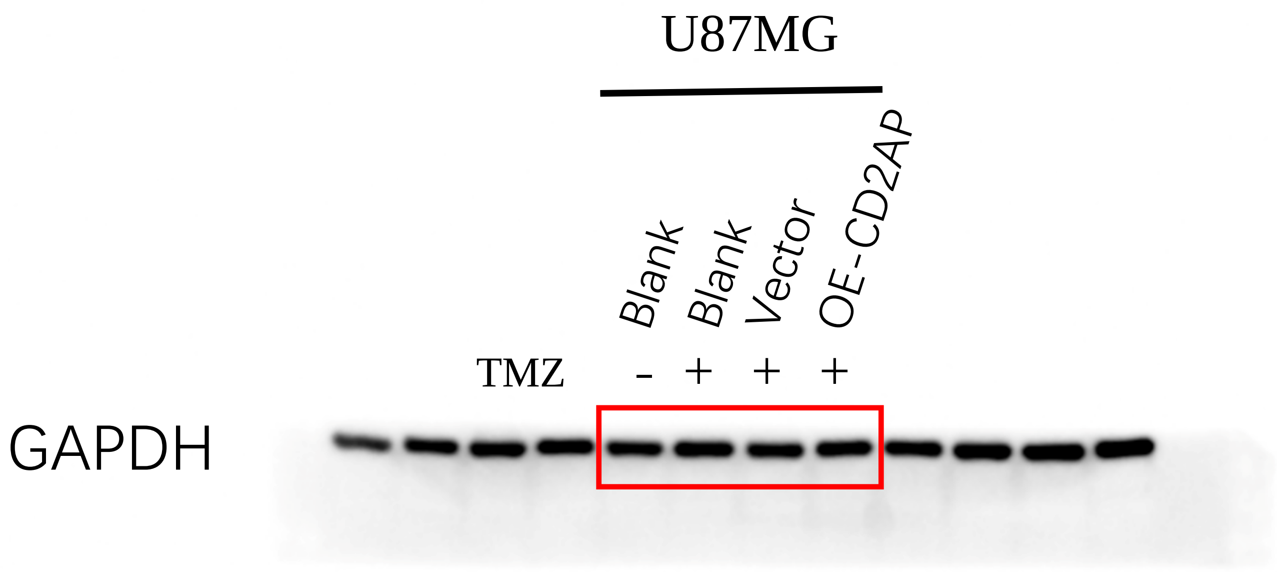

The membrane was imaged with Azure Biosystems 300

Full unedited gel for Figure Supplement 6A

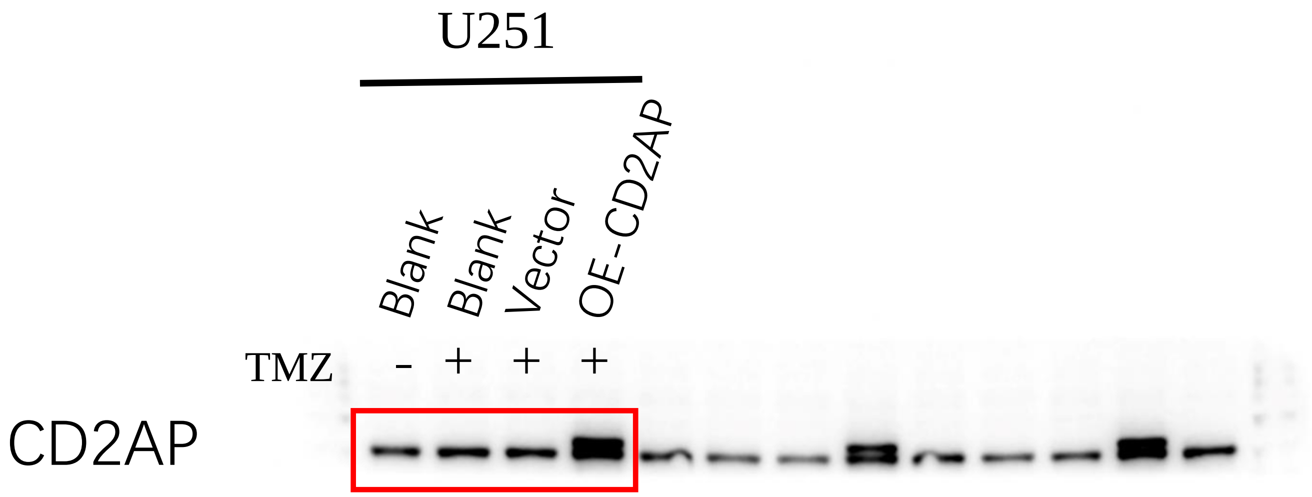

The membrane was imaged with Azure Biosystems 300

Full unedited gel for Figure Supplement 6A

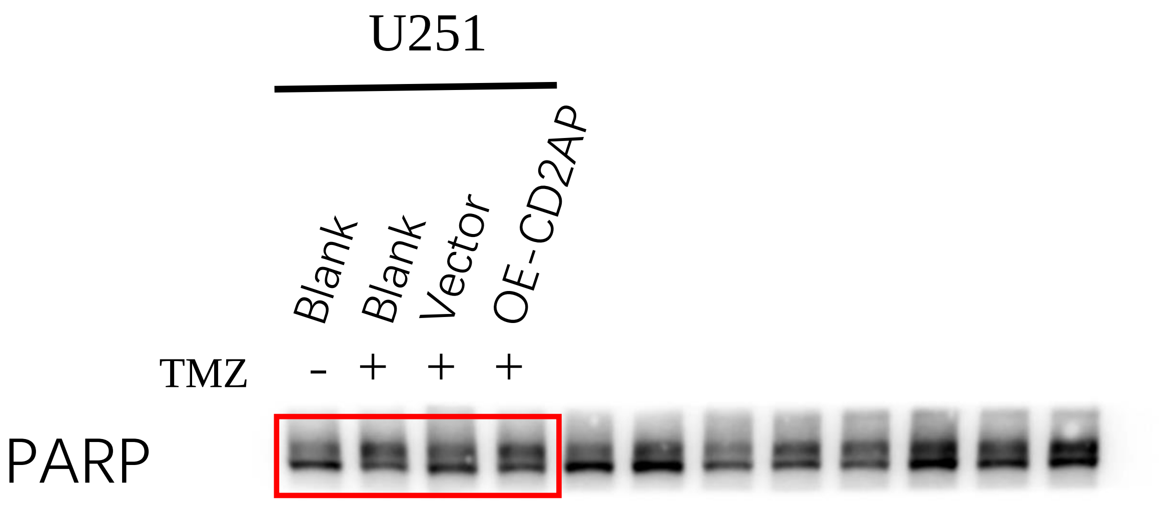

The membrane was imaged with Azure Biosystems 300

Full unedited gel for Figure Supplement 6A

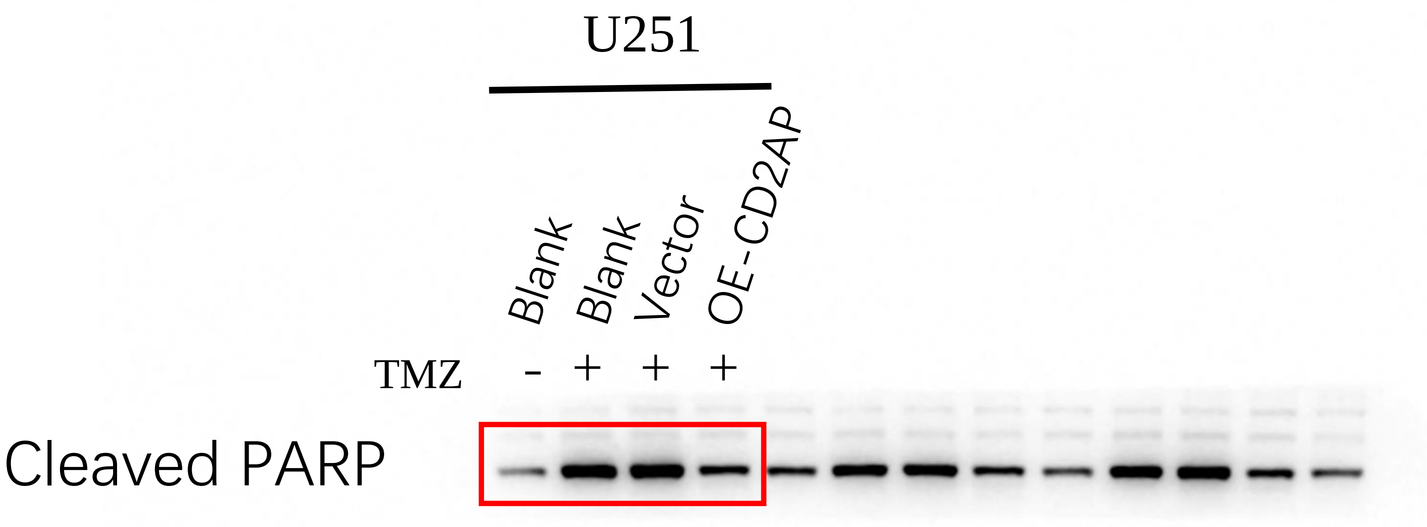

The membrane was imaged with Azure Biosystems 300

Full unedited gel for Figure Supplement 6A

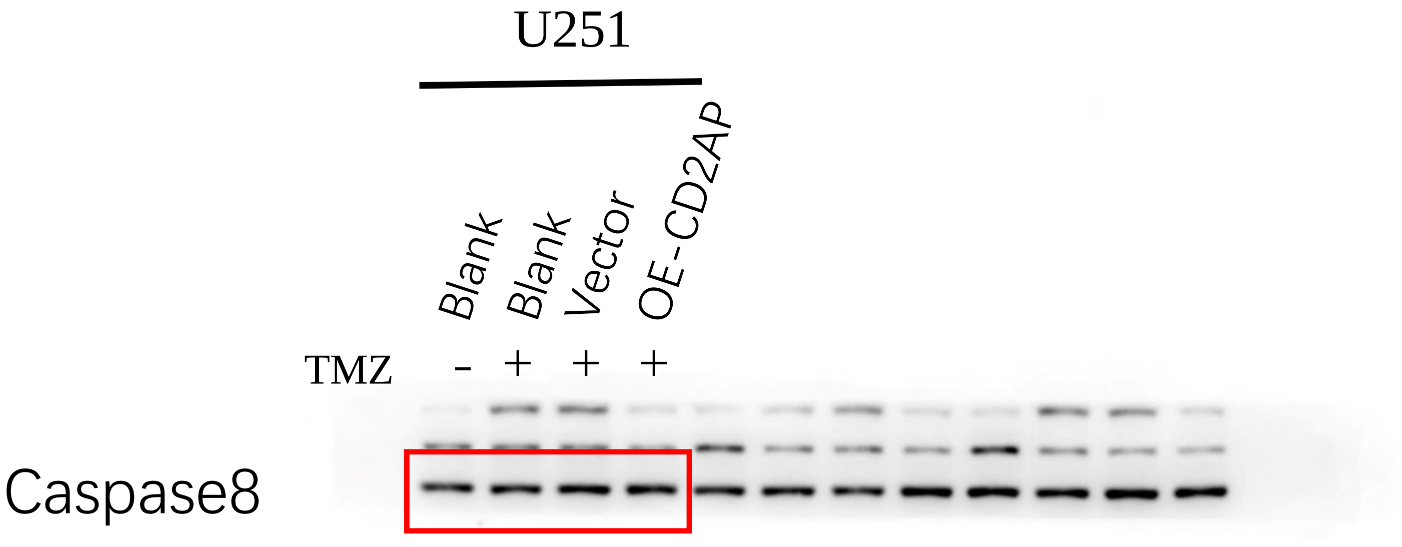

The membrane was imaged with Azure Biosystems 300

Full unedited gel for Figure Supplement 6A

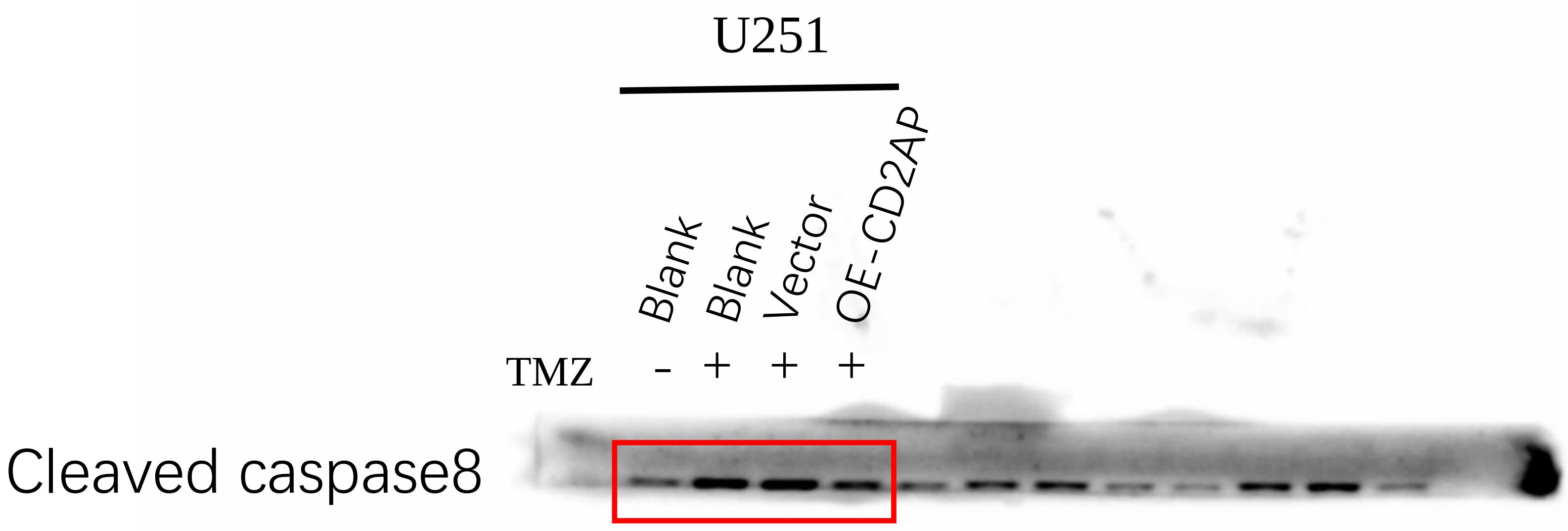

The membrane was imaged with Azure Biosystems 300

Full unedited gel for Figure Supplement 6A

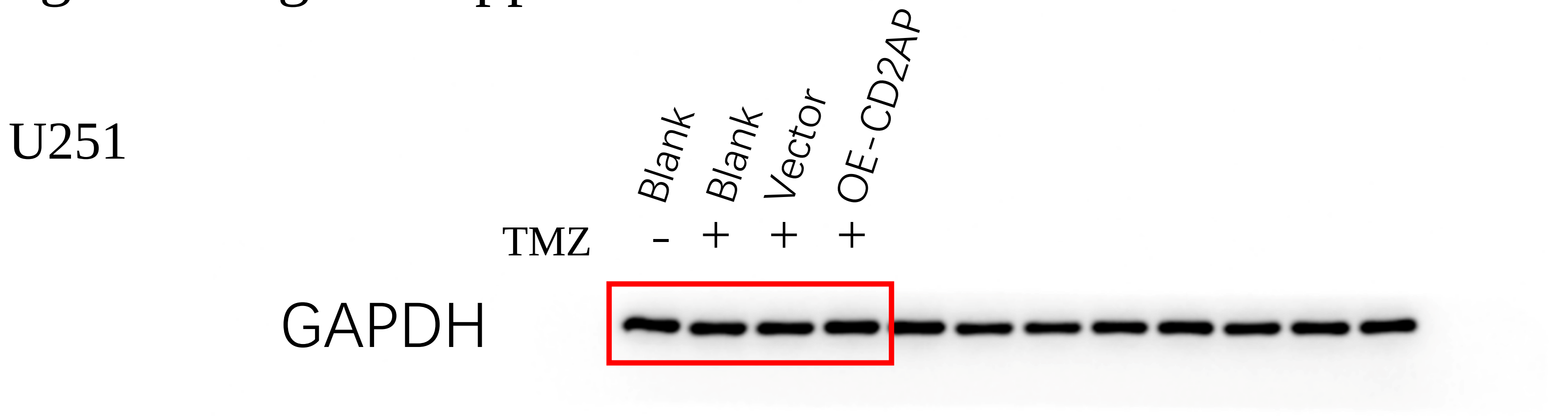

The membrane was imaged with Azure Biosystems 300

Full unedited gel for Figure Supplement 6D

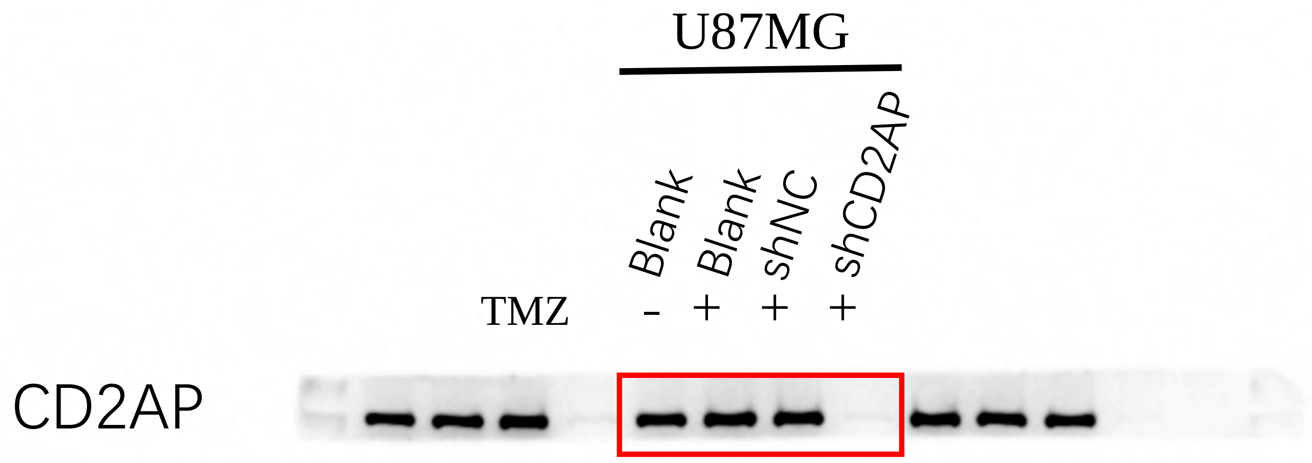

The membrane was imaged with Azure Biosystems 300

Full unedited gel for Figure Supplement 6D

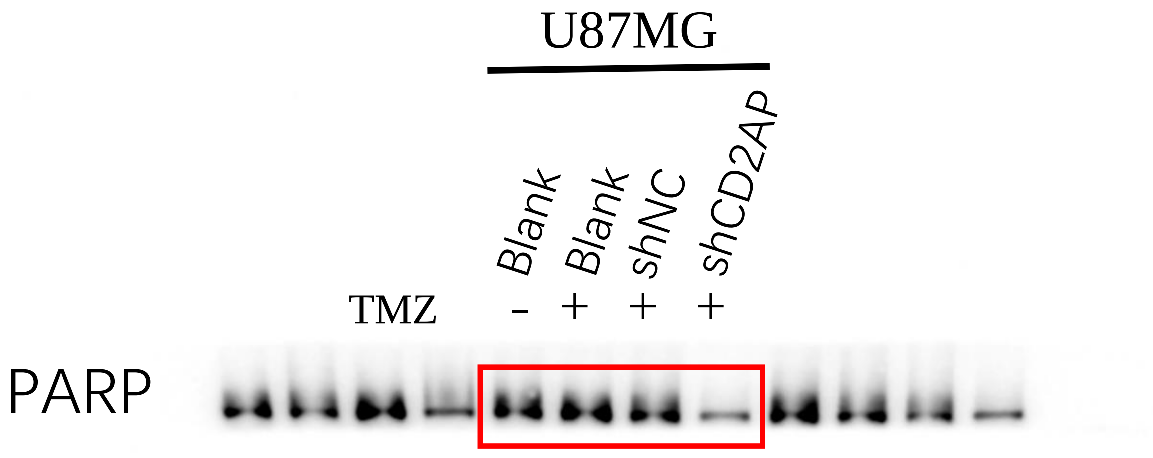

The membrane was imaged with Azure Biosystems 300

Full unedited gel for Figure Supplement 6D

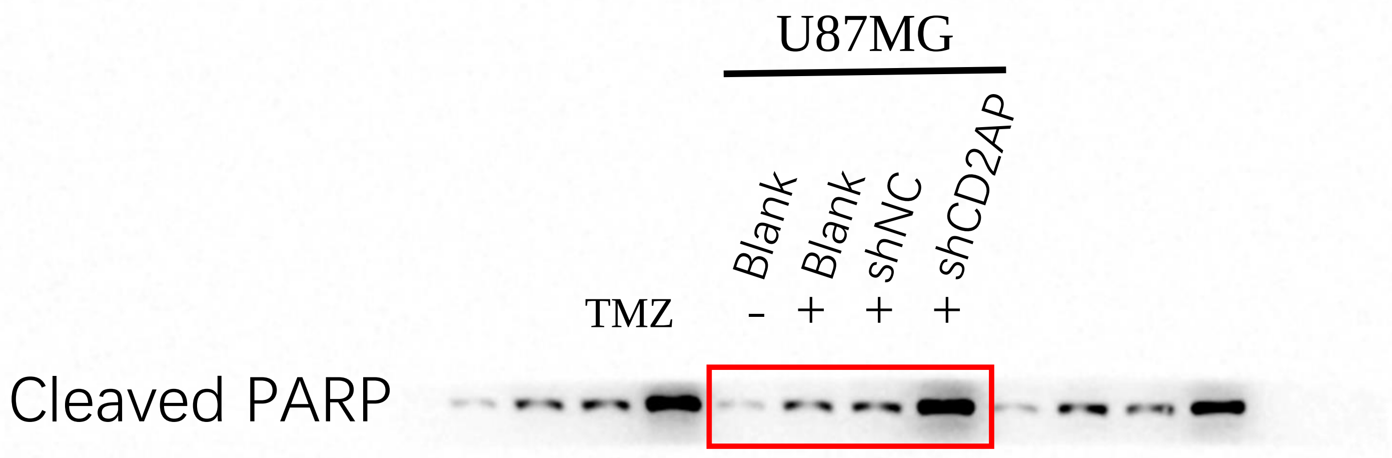

The membrane was imaged with Azure Biosystems 300

Full unedited gel for Figure Supplement 6D

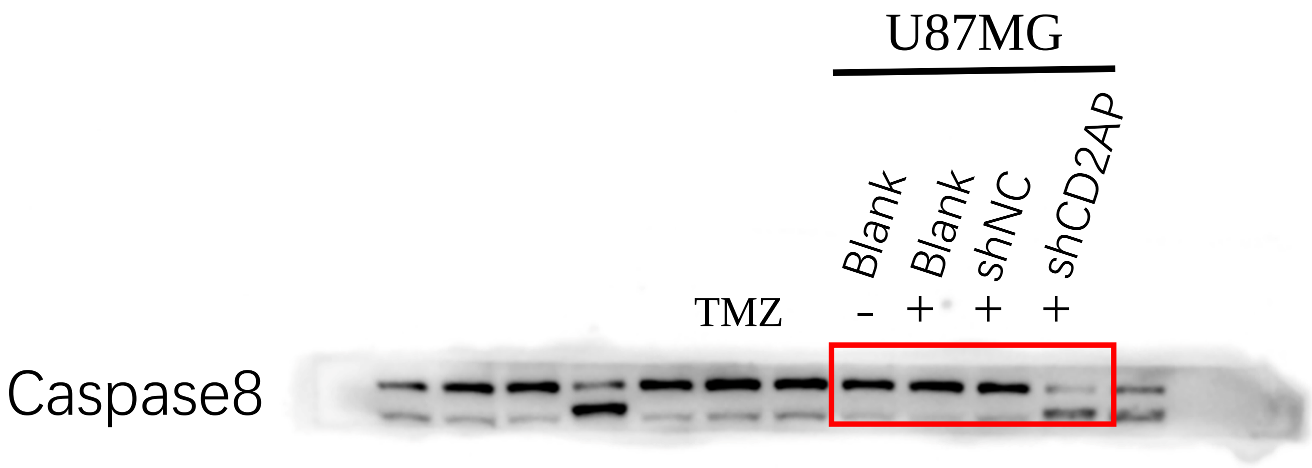

The membrane was imaged with Azure Biosystems 300

Full unedited gel for Figure Supplement 6D

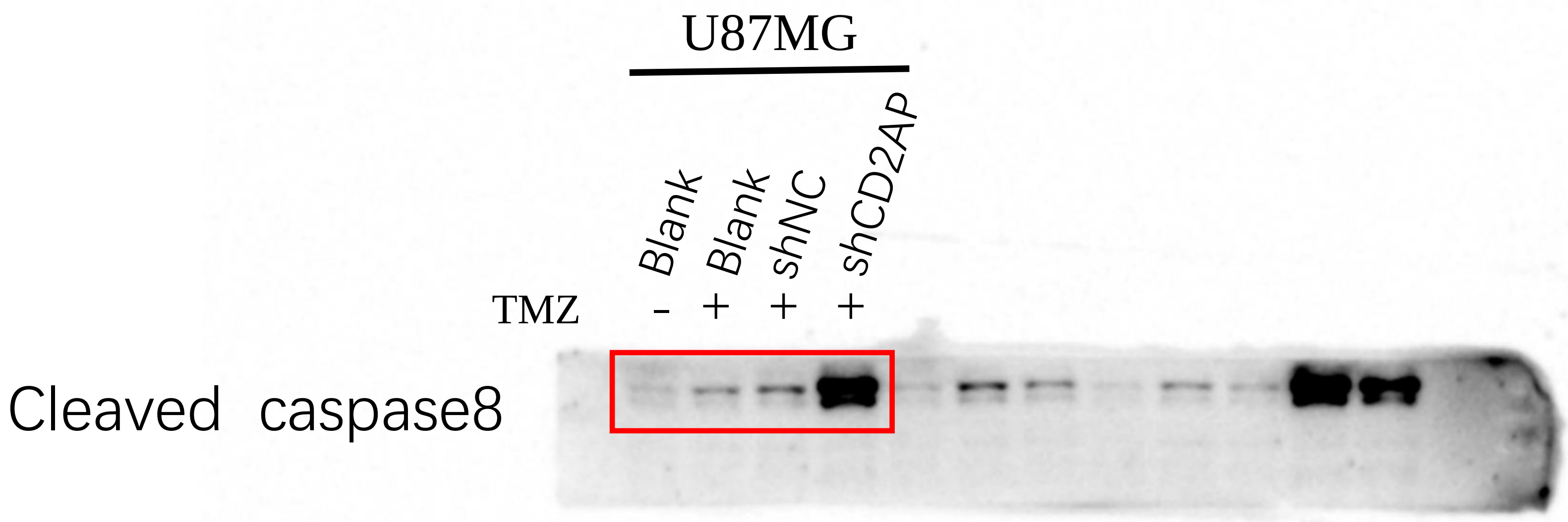

The membrane was imaged with Azure Biosystems 300

Full unedited gel for Figure Supplement 6D

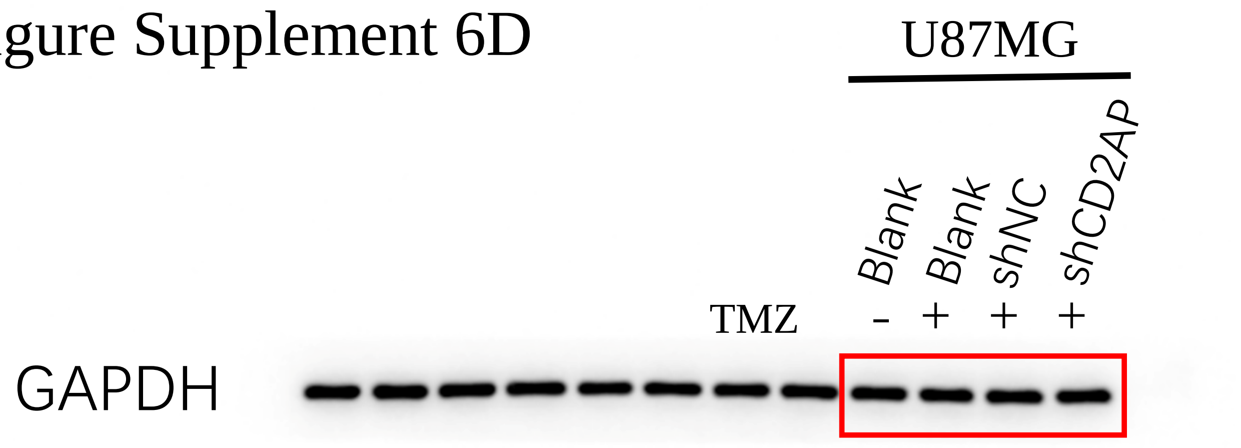

The membrane was imaged with Azure Biosystems 300

Full unedited gel for Figure Supplement 6D

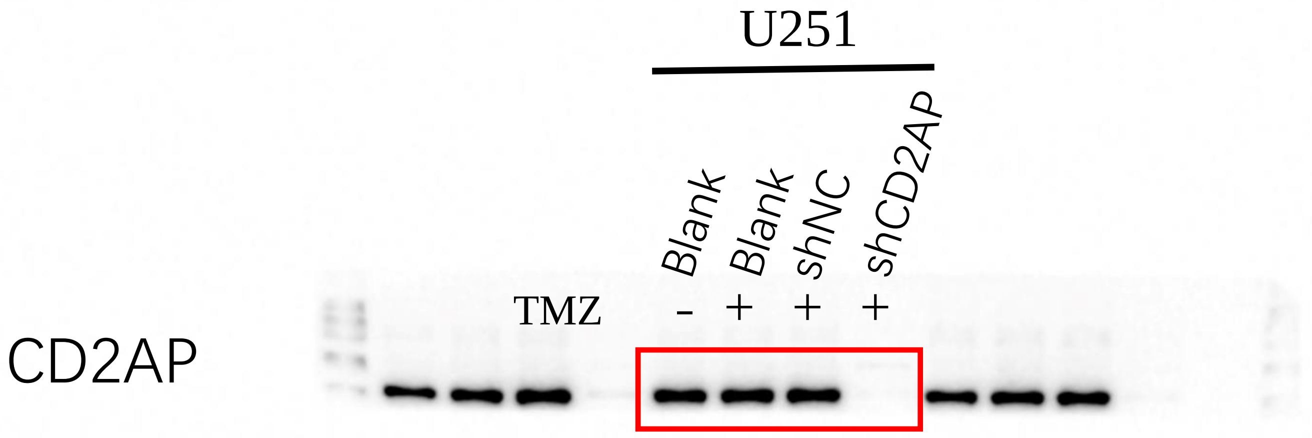

The membrane was imaged with Azure Biosystems 300

Full unedited gel for Figure Supplement 6D

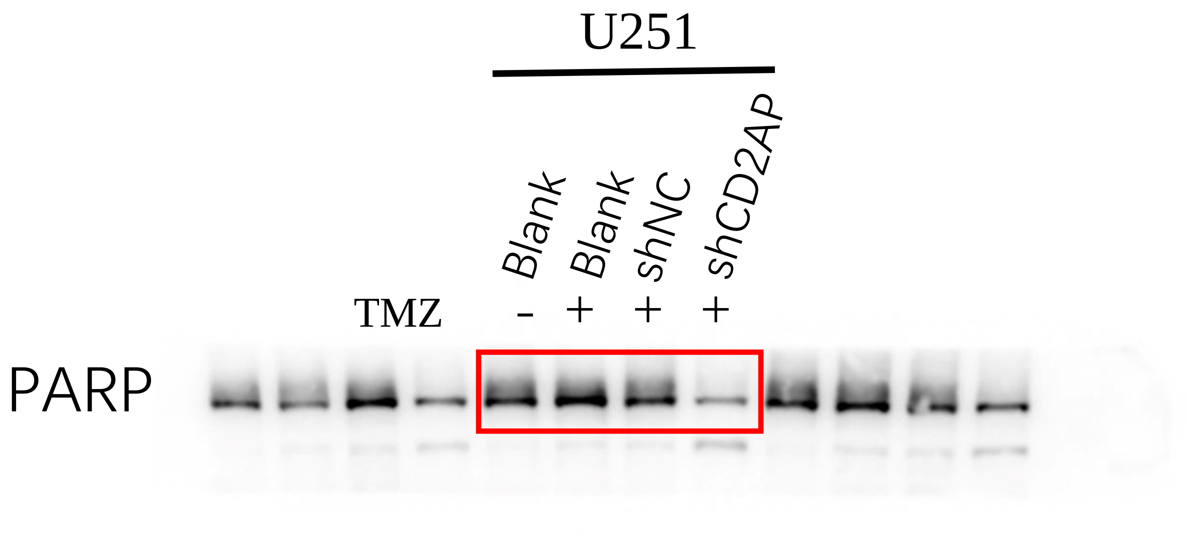

The membrane was imaged with Azure Biosystems 300

Full unedited gel for Figure Supplement 6D

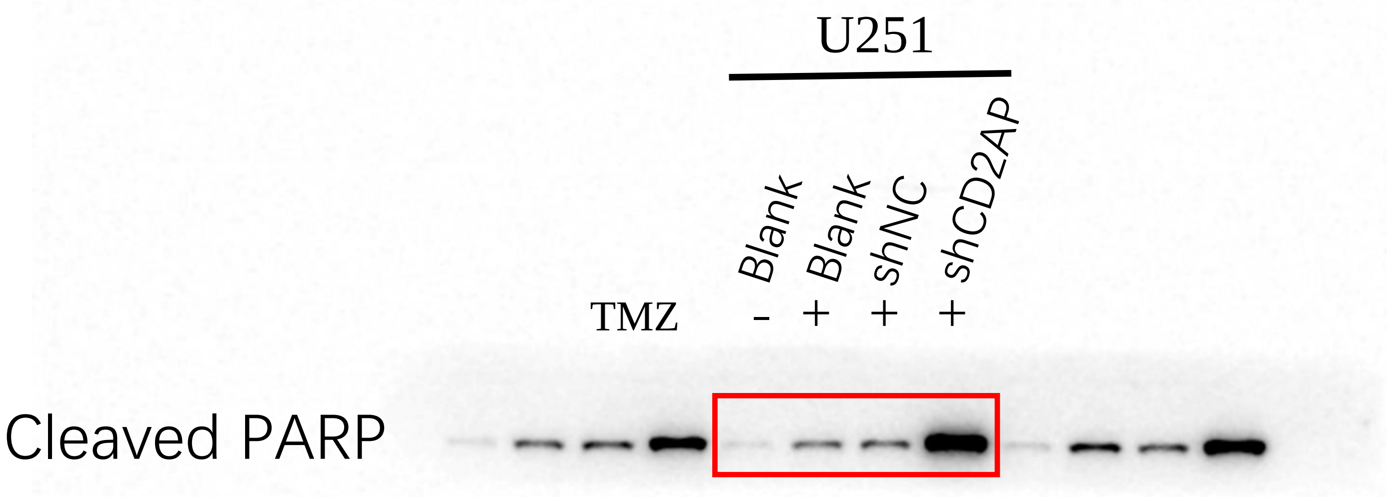

The membrane was imaged with Azure Biosystems 300

Full unedited gel for Figure Supplement 6D

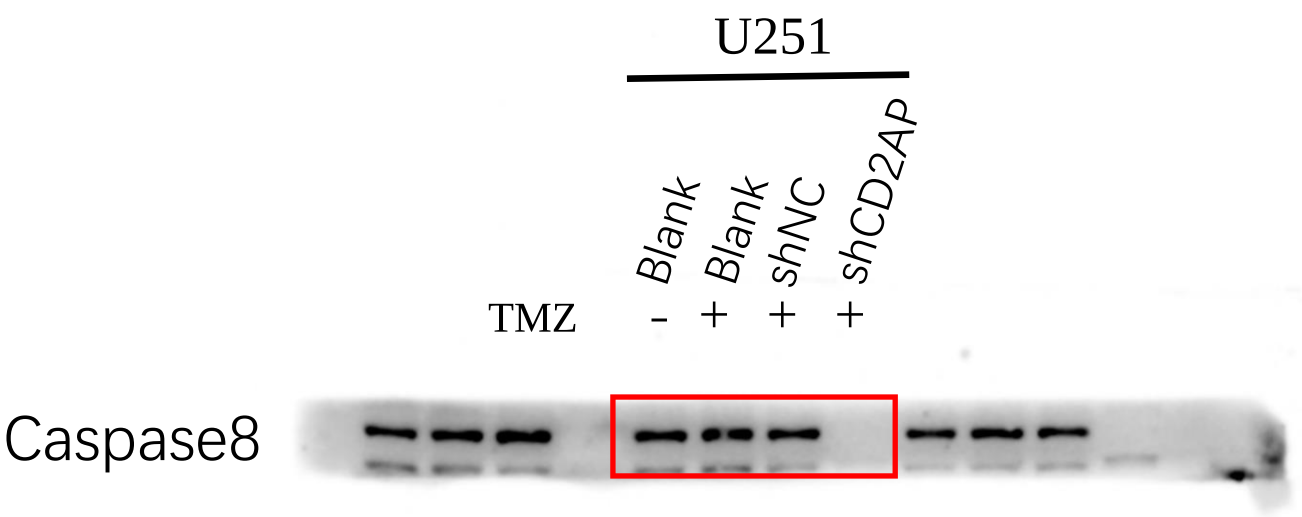

The membrane was imaged with Azure Biosystems 300

Full unedited gel for Figure Supplement 6D

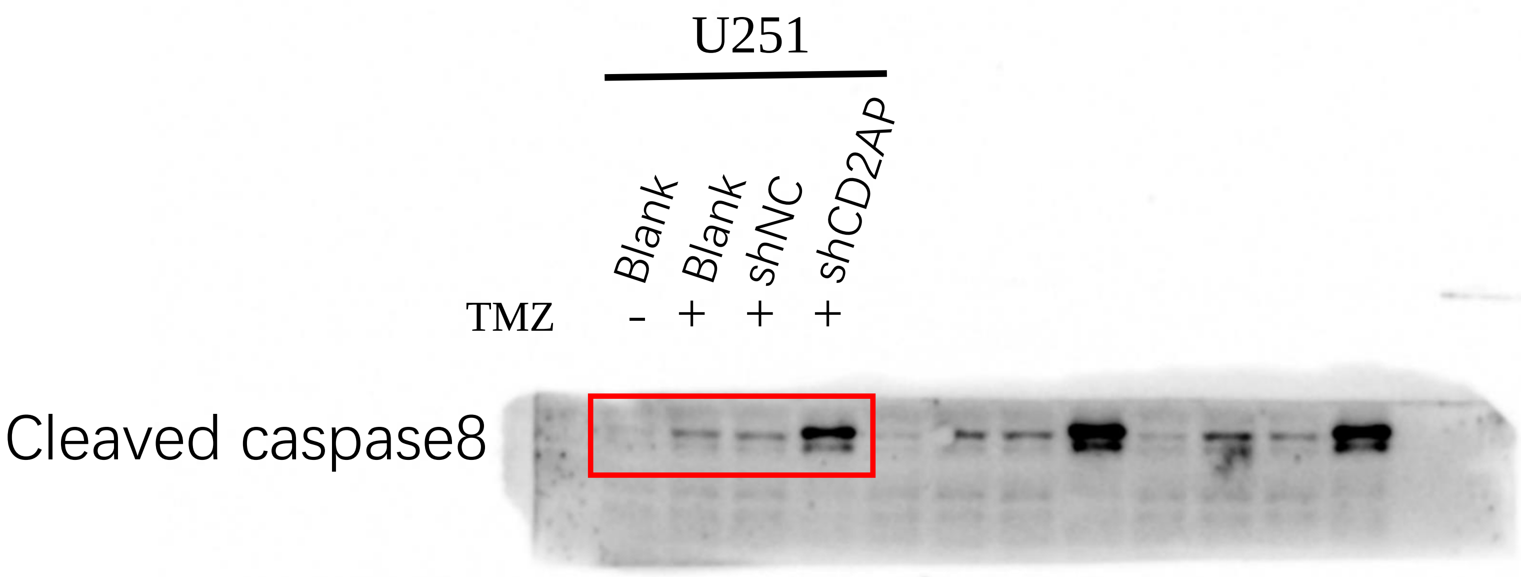

The membrane was imaged with Azure Biosystems 300

Full unedited gel for Figure Supplement 6D

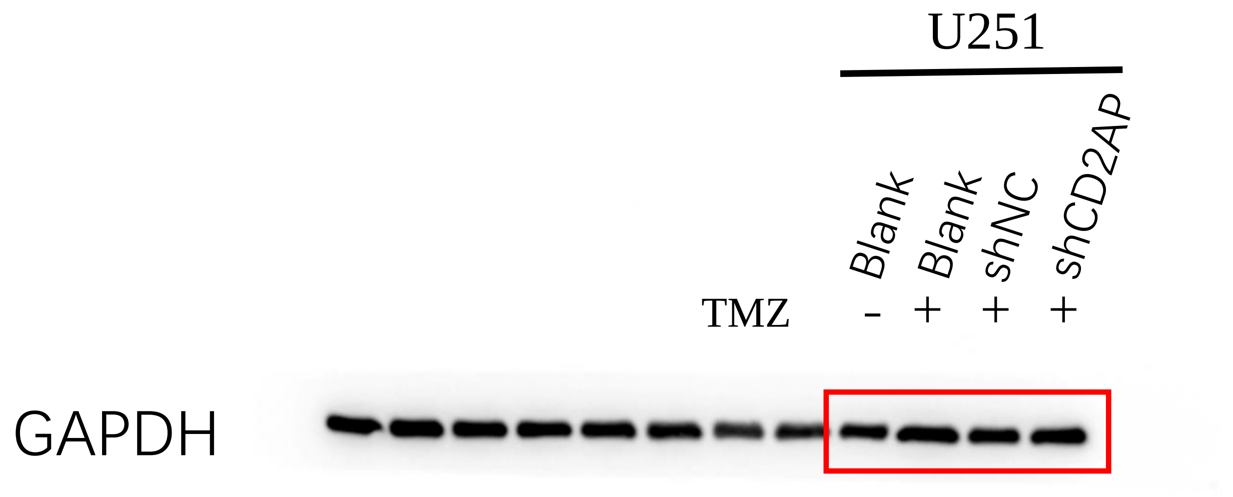

The membrane was imaged with Azure Biosystems 300
